# A Global Synthesis on Land‐Cover Changes in Watersheds Shaping Freshwater Detrital Food Webs

**DOI:** 10.1111/gcb.70380

**Published:** 2025-08-04

**Authors:** Rebecca Oester, François Keck, Marcelo S. Moretti, Florian Altermatt, Andreas Bruder, Verónica Ferreira

**Affiliations:** ^1^ Institute of Microbiology, University of Applied Sciences and Arts of Southern Switzerland Mendrisio Switzerland; ^2^ Department of Evolutionary Biology and Environmental Studies University of Zurich Zurich Switzerland; ^3^ Department of Aquatic Ecology Eawag: Swiss Federal Institute of Aquatic Science and Technology Dübendorf Switzerland; ^4^ Laboratory of Aquatic Insect Ecology Universidade Vila Velha Vila Velha ES Brazil; ^5^ MARE—Marine and Environmental Sciences Centre, ARNET—Aquatic Research Network, Department of Life Sciences University of Coimbra Coimbra Portugal

**Keywords:** allochthonous matter processing, aquatic‐terrestrial linkages, decomposers, decomposition, deforestation, detritivores, detritus, meta‐analysis, shredders, stream biodiversity

## Abstract

Anthropogenic land‐cover changes are among the most pressing global threats to both aquatic and terrestrial ecosystems, jeopardizing biodiversity and the critical connections between these systems. Resource flows and trophic interactions intricately link aquatic and terrestrial ecosystems, with terrestrial‐derived detritus playing a fundamental role in supporting aquatic food webs. These detrital inputs form essential cross‐ecosystem linkages, underpinning key ecological processes and providing vital resources for aquatic communities. Yet, little research has focused on how land‐cover changes cascade across this linkage. To better understand how land‐cover changes in the watershed influence freshwater detrital food webs, we conducted a meta‐analysis of field studies reporting the effects of vegetation changes on freshwater detrital consumers and organic matter decomposition. The results from 144 studies, reporting 1235 comparisons, showed that, overall, land‐cover changes in the watershed vegetation, especially through harvest and land‐use conversion, have negative effects on aquatic biodiversity and ecosystem processes. These vegetation changes reduced diversity, abundance, and biomass across multiple trophic levels in freshwater detrital food webs. Studies examining multiple organism groups most often observed negative responses across multiple trophic levels, suggesting that these land‐cover changes negatively affected multiple detrital food‐web components simultaneously. Our results also show that outcomes of restoration of watershed vegetation were context‐dependent, and no clear trend of improvement was visible. Therefore, conservation of natural riparian and catchment vegetation is key to maintaining freshwater ecosystem processes and aquatic biodiversity worldwide, and more efficient and evidence‐based restoration measures are urgently needed. As our global synthesis shows that direct human‐induced alterations of vegetation in watersheds have significant negative effects on freshwater detrital food webs, there is a pressing need to consider cross‐ecosystem consequences of land‐cover changes in conservation and ecosystem management.

## Graphical abstract

This study investigates the cascading effects of land‐cover changes on freshwater food webs and biodiversity by synthesizing data from 144 published studies. We found that human‐driven changes in watershed vegetation consistently reduce the diversity, abundance, and biomass of freshwater organisms across trophic levels. These findings highlight the urgent need to conserve natural riparian and catchment vegetation and to implement more effective, cross‐ecosystem restoration strategies to maintain freshwater biodiversity and ecosystem functioning globally.

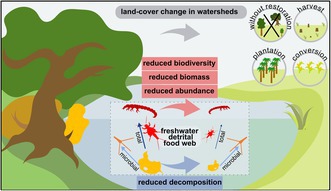

## 1 Introduction

Human‐driven land‐cover changes pose major threats to ecosystems worldwide, jeopardizing biodiversity, ecosystem processes, and cross‐ecosystem connections (IPBES 2019; Keck et al. 2025). These changes can trigger cascading effects on adjacent and downstream water bodies (England and Rosemond 2004; Hanna et al. 2020) reducing freshwater biodiversity, impairing ecosystem functions, and weakening or even disrupting terrestrial–aquatic linkages (Allan 2004; Cereghetti and Altermatt 2023; Little and Altermatt 2018; Oester et al. 2023). Land‐use/land‐cover change is, in fact, one of the main direct drivers of aquatic biodiversity loss (Allan 2004; Harrison et al. 2018; IPBES 2019; Tickner et al. 2020; Zhang et al. 2023). In Europe and North America alone, an estimated 80% of natural riparian vegetation has been lost over the past 200 years (Naiman et al. 1993). More broadly, vast areas of riparian and catchment forests have already disappeared across most biomes, with the remaining fragments continuing to degrade—especially in the tropics (Barnes et al. 2017; Dudgeon 2000, 2006; Reid et al. 2019; Warren et al. 2016). However, the extent of deforestation, shifts in vegetation composition, and biodiversity loss vary across regions, as do the underlying drivers of these changes (Boyero et al. 2012; Kominoski et al. 2013; Rudel et al. 2009).

Vegetation changes caused by tree harvesting, exotic timber plantations, and land conversion for agriculture can impact aquatic food webs by altering consumer abundance, biodiversity and biomass, and organic matter decomposition (Frainer and McKie 2021; Harding et al. 1998; Silva‐Araújo et al. 2020). For example, forest harvesting can reduce macroinvertebrate biodiversity (Hernandez et al. 2005; Reid et al. 2010), whereas *Eucalyptus* spp. plantations can slow leaf litter decomposition (Ferreira et al. 2016, 2019). Additionally, land‐use conversion can shift ecosystem metabolism from heterotrophy toward autotrophy (Hagen et al. 2010; Hebert et al. 2023), as the reduction in riparian vegetation decreases terrestrial organic matter inputs (England and Rosemond 2004; Hanna et al. 2020), while the associated loss of shading enhances in‐stream primary production (Jyväsjärvi et al. 2022). Human‐induced changes in watershed vegetation can also modify microclimate, sediment and nutrient retention, bank stability, and stream hydrology (Ferreira et al. 2023; Tolkkinen et al. 2020), sometimes resulting in nonlinear responses and long‐lasting legacy effects (Frainer and McKie 2021). Collectively, these shifts in land cover can affect not only the quantity of basal resources but also their quality and composition, with important implications for the trophic structure of freshwater detrital food webs (Campanyà‐Llovet et al. 2017; Larrañaga et al. 2023; Oelbermann and Gordon 2000). The spatial scale of land‐cover change, whether locally in the riparian zone or across the entire catchment, can have varying effects on streams. For example, exotic tree plantations negatively impact leaf litter decomposition more severely when present in both the catchment and the riparian zone than when only in the catchment (Ferreira et al. 2016). Given the rapid and widespread land‐cover changes, it is necessary to assess their consequences across ecosystem boundaries to “bend the curve of global freshwater biodiversity loss” (Tickner et al. 2020).

Aquatic and terrestrial ecosystems are closely linked through resource flows and trophic interactions at their interfaces (Baxter et al. 2005; Gounand et al. 2018). An important terrestrial–aquatic linkage is based on the influx, transport, deposition, and decomposition of organic matter from the riparian vegetation in water bodies (Elosegi et al. 2007; England and Rosemond 2004; Scherer‐Lorenzen et al. 2022; Stoler and Relyea 2020). These cross‐ecosystem resource flows shape communities (Oester et al. 2023; Silva‐Araújo et al. 2020) and energy pathways in the aquatic food web (Ho et al. 2023; Jacquet et al. 2022; Oester et al. 2024). In many landscapes, native trees dominate the riparian and catchment vegetation, particularly in headwater regions (Tonello et al. 2021; Västilä and Järvelä 2018). Detritus, including leaf litter, branches, or twigs that enter the water body, fuel the detrital food web and support aquatic consumers (Baxter et al. 2005; Díez et al. 2002; Gessner et al. 2010; Nakano and Murakami 2001).

When terrestrial detritus enters a water body, a suite of microbial decomposers, detritivorous macroinvertebrates, and other consumers feed on and break down this allochthonous resource (Gessner et al. 2010; Menninger and Palmer 2007). Microbes, including aquatic fungi and heterotrophic bacteria, consume detritus themselves and enhance its palatability for higher level consumers through nutrient immobilization and maceration (García‐Palacios et al. 2017; Tolkkinen et al. 2015, 2020). Detritivorous macroinvertebrates, particularly the functional feeding group of shredders (*sensu* Moog 1995), preferentially feed on microbially conditioned detritus (Arsuffi and Suberkropp 1985; Graça and Cressa 2010) and further fragment this material (Graça 2001). Omnivores, like other macroinvertebrates, fish, and crayfish, opportunistically use terrestrial detritus as a resource (Allen et al. 2024; Dudley et al. 2021; Evangelista et al. 2014). Biodiversity at multiple trophic levels in the freshwater detrital food web can accelerate the decomposition process (Gessner et al. 2010; Handa et al. 2014). Hence, decomposition is a key ecosystem function that integrates feeding activity, trophic relationships, and nutrient cycling in the detrital food web, yet responds sensitively to anthropogenic change (Hebert et al. 2023). To fully understand the freshwater food‐web dynamics and their responses to environmental change, it is essential to assess the components of the detrital food web—both terrestrial resources and aquatic consumers—and the decomposition processes that link them.

Here, we report a meta‐analysis of 144 field studies examining the effects of direct human‐induced watershed vegetation change (i.e., restoration, plantation, harvest, and land‐use conversion) on freshwater detrital food webs and published between 1993 and 2023. Using a systematic approach, we (i) determined the significance, magnitude, and direction of the mean effect of vegetation change on freshwater detrital communities and organic matter decomposition, (ii) assessed which moderators (i.e., explanatory variables) influence the magnitude and direction of the overall effects and the effects for each trophic level separately, and (iii) assessed whether studies observe similar directions of effect sizes across trophic levels. We expected to find (i) an overall negative effect of vegetation change on freshwater detrital food webs, (ii) especially in the tropics and subtropics (as the climatic regions undergoing currently the most drastic land‐cover changes), where the watershed vegetation was completely converted to other land‐use types, where watershed vegetation is entirely (i.e., in the riparian zone and in the catchment) altered, for diversity and biomass metrics and for microbe and shredders (as the most detritus‐dependent groups), and (iii) that these effects are simultaneously detectable at multiple trophic levels of freshwater detrital food webs (Barnes et al. 2017; Burwood et al. 2021; Casotti et al. 2015; Cornejo et al. 2020).

## 2 Materials and Methods

### 2.1 Literature Search and Study Selection

**FIGURE 1:**
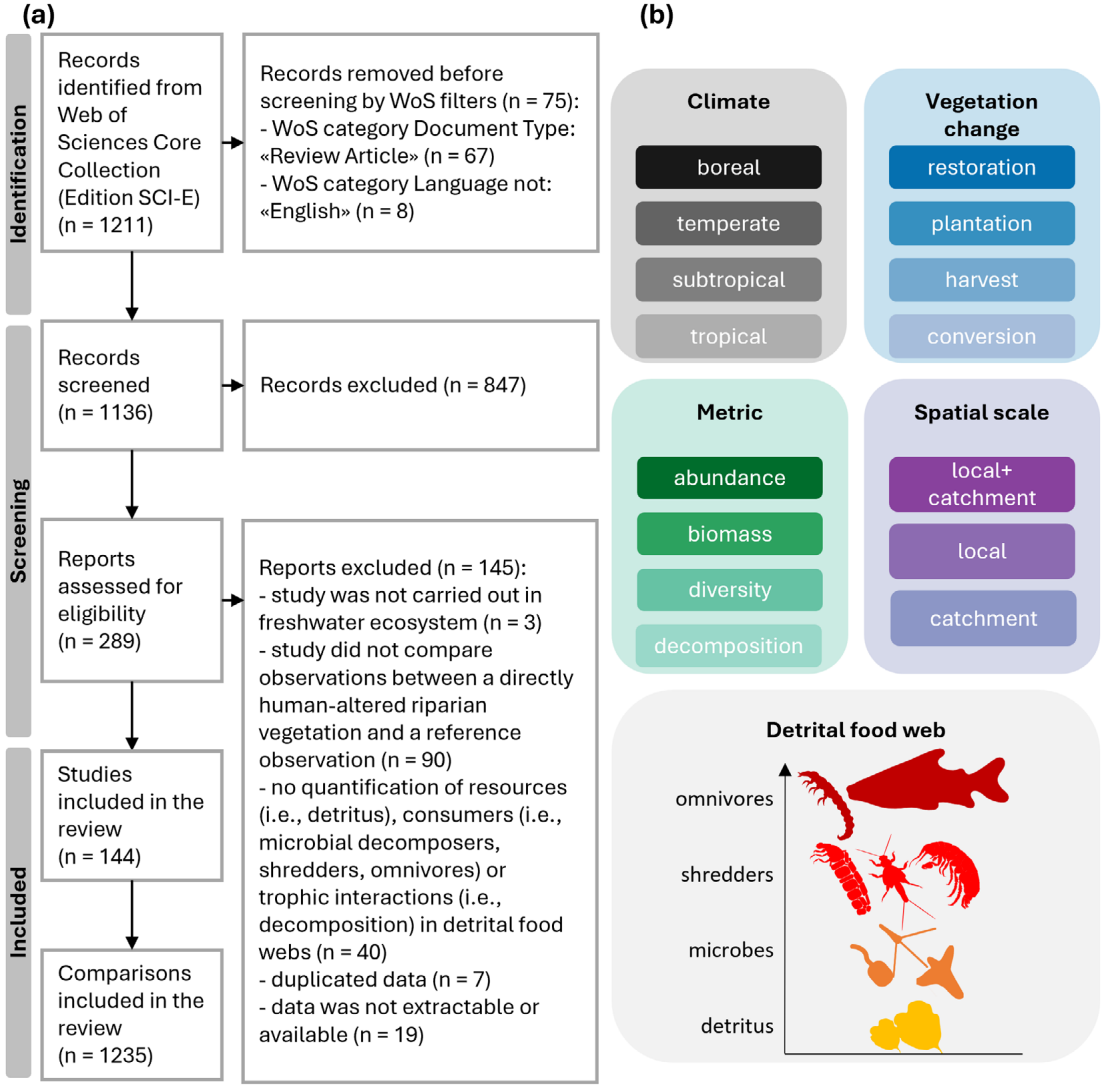
Overview of methods used in this review. (a) PRISMA flow diagram with the number of studies located in the literature search and study selection and (b) Main moderators extracted for the assessment of the effects of vegetation change on freshwater detrital food webs.

We conducted a systematic literature search using the ISI Web of Science (WoS) Core Collection (Edition: Science Citation Index Expanded) to identify records examining the consequences of direct human alterations in land cover in the riparian zone and catchment on elements of freshwater detrital food webs (i.e., detritus, microbial decomposers, shredders, and omnivores; Figure 1a). Specifically, we searched for empirical studies comparing community metrics (i.e., α‐diversity, abundance, biomass) or organic matter decomposition in freshwater detrital food webs with a before‐after or a control‐impact design. Reference conditions are defined as near‐pristine or natural watershed vegetation such as native riparian forests, whereas direct human‐induced alterations in the vegetation could consist of plantations, vegetation restoration activities, land conversion (e.g., from forest to pasture), or forest harvest, but not damages caused by storms, wildfires, or exotic species. We also a priori excluded comparisons between reference and urban sites, as the vegetation in urban sites is often replaced by impervious surfaces (Maloney and Weller 2011).

We used the following search string in the search field “topic”, which searches title, abstract, and keywords: (stream OR river OR lake OR pond OR freshwater) AND (“land use” OR “land cover” OR deforestation OR logging OR fragmentation OR “forest composition” OR plantation OR forestry OR restoration OR conservation OR revitalization OR revitalisation OR clear* OR harvest* OR removal) AND (riparian OR forest OR buffer) AND (decomposition OR processing OR breakdown OR decay OR brown) AND (“food web” OR trophic OR detritivor* OR shredder OR *invertebrate OR decomposer OR hyphomycete OR fung* OR bacteri* OR microb*). We searched for records published before 16 April 2024, which yielded 1211 records. We then applied the WoS filters of Document Type to exclude reviews (*n* = 67) and Non‐English Records (*n* = 8) (Figure 1a).

Next, we screened titles and abstracts of 1136 records to determine whether the record had potential to contain suitable data, using the following criteria: (1) the study was carried out in a freshwater ecosystem; (2) the study compared field observations or experimental outcomes between a directly human‐altered watershed or riparian vegetation and a reference condition; and (3) the study quantified resources (i.e., detritus), detritus‐consuming communities (i.e., microbial decomposers, shredders, omnivores at least partially consuming detritus) or trophic interactions (i.e., decomposition). We excluded records that did not meet these three criteria (*n* = 847). In a next step, we read the full text of the 289 remaining records and excluded 145 additional records that did not fulfill the three abovementioned criteria. We also excluded records that reported duplicate data (*n* = 7) or where data was not extractable or available (*n* = 19). The final set of records included 144 studies. For further details on the identification and screening process according to the Preferred Reporting Items for Systematic reviews and Meta‐Analyses (PRISMA; O'Dea et al. 2021; Page et al. 2021) see Figure 1a.

### 2.2 Extraction of Primary Data

A single study could report several comparisons between reference and human‐altered conditions if using, for example, different litter species (e.g., Cornejo et al. 2020; Masese et al. 2014), consumer groups (e.g., Encalada et al. 2010), community metrics (e.g., Oester et al. 2023), or regions (e.g., Ferreira et al. 2019; Riipinen et al. 2009), resulting in a total of 1235 comparisons (Figure 1a). We did not extract data that were not unambiguously comparable. These cases include: (1) different levels of the same type of vegetation change across a gradient (e.g., “low,” “medium,” and “high” logging intensity), in which case we compared the two extreme ends of the gradient; (2) different taxonomic levels (e.g., family, genus, species) of the same community, in which case we took the finest available level (i.e., species if possible); (3) different calculations for similar diversity metrics (e.g., Shannon index, richness, evenness), in which case we extracted richness if provided, or different decomposition quantifications (e.g., % mass loss, decomposition rates), in which case we extracted decomposition rates in degree days if provided. We recorded the mean, variation (i.e., standard deviation (SD), standard error (SE), or confidence interval (CI)), and number of replications for each metric in the reference and altered condition being compared.

We extracted data from text, tables, figures, or requested them from authors. We used WebPlotDigitizer v.5.0 (https://automeris.io/WebPlotDigitizer/), in manual mode, to extract values from figures, which corresponded to 37.8% of all comparisons. If variation in the primary studies was reported or provided by the authors as SE or CI, we converted it into SD. However, no measure of variation was available in 7.7% of cases, and we estimated missing SD values by imputation based on the cases in the dataset that reported SD values associated with the same metric and trophic level (Lajeunesse 2013). Extracting data from figures and imputing missing variation values can potentially introduce a bias in the dataset. Consequently, we assessed the possible bias introduced by these potentially low‐quality data through sensitivity analyses (see below).

### 2.3 Extraction of Moderators

Several biotic and abiotic explanatory variables (i.e., moderators) may affect the magnitude and direction of the response of elements of freshwater detrital food webs to human‐induced changes in the watershed vegetation. Based on our hypotheses, we included five general moderators (Figure 1b, Table S1): climate (Ferreira et al. 2019), type of vegetation change (Boyero et al. 2011; Goodman et al. 2006; Inoue et al. 2012), spatial scale of vegetation change (Ferreira et al. 2016), community or decomposition metric (Iñiguez‐Armijos et al. 2018; Oester et al. 2023), and trophic level (Hebert et al. 2023). We also recorded type of water body (i.e., lake, pond, river, stream, and bromeliad), but as more than 99% of all comparisons were based on streams (Table S1), we did not consider this moderator for further analyses.

We extracted watershed vegetation type of reference and altered sites as well as the main human‐induced change in the altered sites. We later coded the different types of changes into the following four categories: restoration, plantation, harvest, and conversion (Figure 1b, Table S1). For restoration activities, we used the restored site as the reference condition, which allowed us to align it with other types of changes in watershed vegetation. By reversing this comparison, we interpreted the direction of change as indicative of what would have happened without restoration measures. Land‐use conversions mostly included changes from a native forest to pasture, grassland, or other agricultural land uses.

For each study, we also collected the location (latitude and longitude) and climate (i.e., boreal, temperate, subtropical, and tropical; Figure 1b, Table S1). If the exact latitude and longitude were not reported, we extracted locality information (e.g., country, state, and catchment) from manuscript figures, tables, text, or requested it from authors. We also extracted the spatial scale of vegetation change either including both the local riparian vegetation and the catchment (local + catchment), only the local riparian vegetation (local) or only the catchment (catchment) according to the information in the publication or provided by authors (Figure 1b, Table S1).

To quantify the effects of vegetation change on the freshwater detrital food web, we extracted the metric of the food‐web element assessed (i.e., α‐diversity, abundance, biomass, and decomposition) and the trophic group (i.e., detritus, microbes, shredders, and omnivores) (Figure 1b, Table S1). In cases where changes in detritus were assessed, we extracted the organic matter type (i.e., leaf, wood, cotton strip, reproductive material or mixed matter) and collected the taxon name if available and applicable. If both single and mixed species treatments were assessed, we included only single species treatments to avoid mixing effects. We extracted the broad taxonomic group for microbial communities (i.e., microbe, bacteria, and fungi), and for secondary consumer communities (i.e., shredders, macroinvertebrates, and fish + crayfish). We also extracted the microhabitat where the communities were collected (i.e., detritus, benthic, and water column). The three community metrics could be measured at all trophic levels, whereas the decomposition metrics (i.e., microbial decomposition, shredder‐mediated decomposition, and total decomposition) were assigned to detritus only.

### 2.4 Effect Size

We calculated the effect size of human‐induced vegetation change on freshwater detrital food webs as standardized mean difference “Hedges' *g*”: (m1_i_ − m2_i_)/pSD_i_, where the mean difference between altered (m1) and reference (m2) conditions is divided by the pooled standard deviation (pSD) (Hedges 1981). Hedges' *g* provides a standardized effect size that accounts for variability within the groups and corrects for bias in small samples, making it useful for comparing studies with different scales or measures (Hedges 1981). Effect sizes of 0 indicate no difference between altered and reference conditions, whereas negative or positive effect sizes indicate that the altered condition shows lower or higher values than the reference condition, respectively. Absolute effect sizes of around 0.2 indicate small effects, 0.5 indicate moderate effects, and 0.8 or higher indicate large effects (Cohen 1988).

### 2.5 Data Analyses

We used mixed‐effect models in R v. 4.4.1 (R Core Team 2024) to test the effect of different climate, type and scale of vegetation change, trophic level, and metric on freshwater detrital food webs. All data and code to reproduce the analysis can be found on Dryad (https://doi.org/10.5061/dryad.c866t1ghg) and Zenodo (https://doi.org/10.5281/zenodo.14289859). To account for heterogeneity within and between studies, we constructed linear random/mixed‐effects models using the variances of each mean as weighing factors and study identity as a random effect (intercept). These models were built using the default settings of the restricted maximum likelihood method in the metafor package (Viechtbauer 2024). Accordingly, we used this type of model to assess the overall mean using the entire dataset and including moderators as fixed effects. We included all moderators separately, analyzing only one moderator at a time to provide an overview of the individual effects of each.

Based on the high heterogeneity level of the dataset, we then split the dataset according to trophic level (i.e., detritus, microbes, shredders, and omnivores) to analyze the interactive effects of moderators and components of the detrital food web separately. For each trophic level, we tested the effects of climate, type and spatial scale of vegetation change, and metric.

We show results as effect size plots using ggplot2 (Wickham et al. 2024). Negative and positive effect sizes indicate lower and higher values in the altered compared to the reference conditions, respectively, with significant effects occurring when 95% CIs do not overlap 0. We calculated the percentage of total variation explained by between‐study variation (*I* ^2^) for the grand mean and separate outcomes for each moderator level for our mixed‐effect models according to Jackson et al. (2012).

Lastly, to assess whether changes in the watershed vegetation influenced multiple trophic levels simultaneously, we analyzed effect sizes of studies with comparisons of at least two trophic levels. We calculated linear regressions and “Spearman” rank correlations using a simple linear model with the median effect size of one trophic level against another trophic level for each study. We show these results as quadrant graphs with studies that provided data that fall within either quadrant of: −/−, +/−, −/+ or +/+ effect size space, with negative and positive values indicating negative and positive effects of change in the vegetation on the median effect size value of a trophic level, respectively.

### 2.6 Publication Bias and Sensitivity Analyses

We assessed publication bias by visually inspecting funnel plots for potential asymmetry driven by small studies (Møller and Jennions 2001). Additionally, we performed a File Drawer Analysis (Rosenberg 2005) to further confirm the robustness of our findings against selective publication. Rosenberg's fail‐safe number (*N*_fs_) provides the number of missing effect sizes showing a nonsignificant effect that would be needed to nullify the mean effect size, with *N*_fs_ > 5 × *n* + 10 (*n* = number of effect sizes) indicating that the dataset can be considered robust to publication bias. Additionally, we applied a newly developed two‐step approach (Yang et al. 2024) for mixed‐effect models to assess publication bias and nonindependence using the orchard (Nakagawa et al. 2023) and clubSandwich packages (Pustejovsky 2024). The results of these analyses (Table S2, Figures S1–S5) suggest that our findings are robust and not unduly influenced by publication bias. Only the subset for omnivores was not robust to publication bias (*N*_fs_ = 1936, *n* = 389). In fact, the bias robust estimate was in the opposite direction to that found based on the dataset (Figure S5), which indicates missing effect sizes to the right of the mean effect size possibly due to the lack of unpublished studies with significantly positive results.

To assess the sensitivity of the results to the inclusion of estimated and imputed data, we repeated the analyses including only reported comparisons. Reasons for concern about the inclusion of data of potentially less quality (i.e., estimated and imputed data) in the analyses would occur if the interpretations of results differed when considering all data or only reported comparisons. We report the sensitivity analysis in the Supplement (Figures S6 and S7, Tables S3 and S4).

## 3 Results

### 3.1 Overview of Studies

**FIGURE 2:**
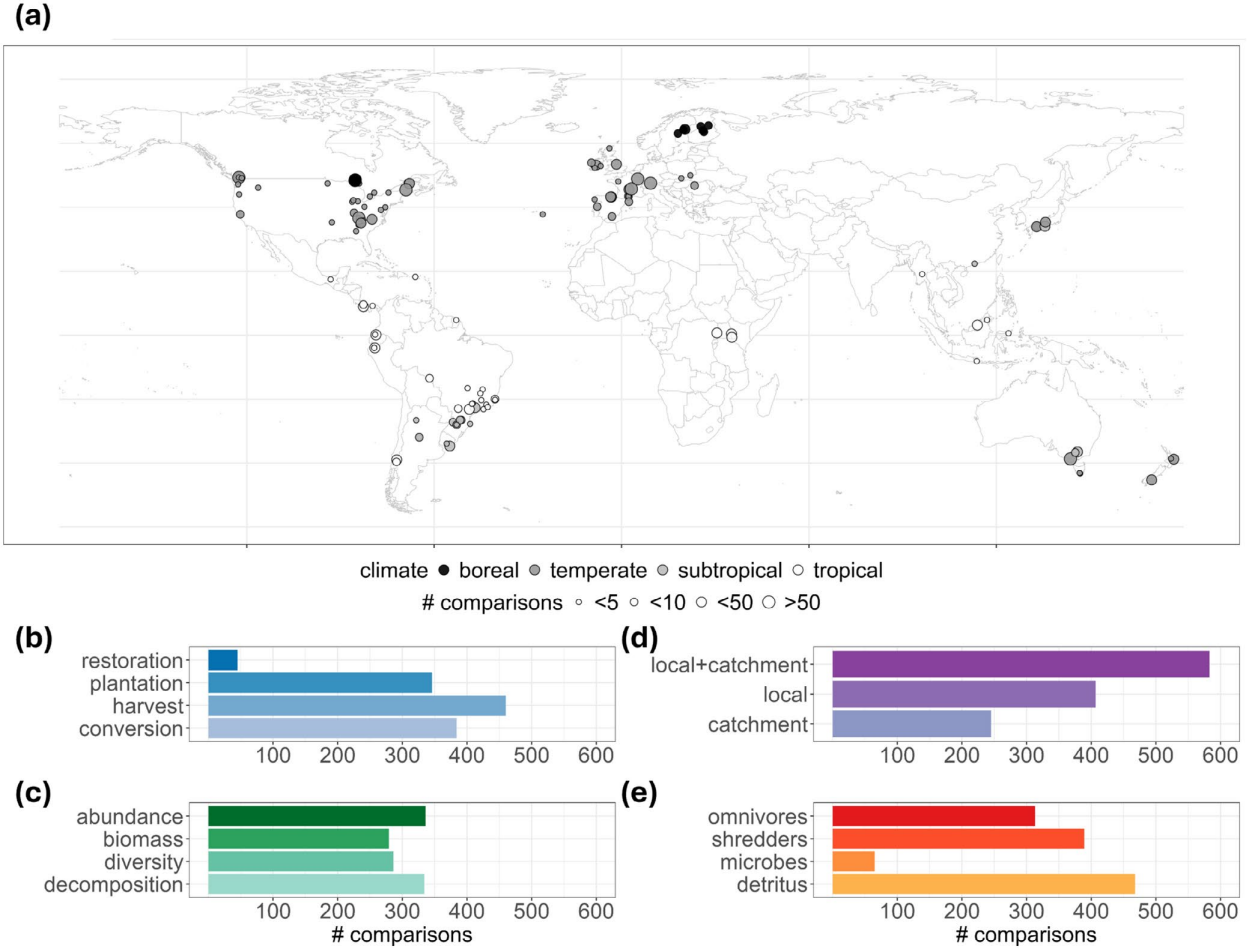
Geographic distribution of studies included in this review and histograms of data distribution of different moderators. (a) World map showing the study locations with each circle representing a study. Map lines delineate study areas and do not necessarily depict accepted national boundaries. Panels (b–e) represent histograms for each level in the moderators: (b) type of vegetation change, (c) spatial scale of vegetation change, (d) metric, and (e) trophic level.

The 144 studies comprising 1235 comparisons covered a broad range of latitudes (−45.29 to 65.57; Figure 2a), types of vegetation changes (Figure 2b) and spatial scale of these changes (Figure 2c), community metrics and decomposition (Figure 2d), and trophic levels (Figure 2e). The studies spanned a 30‐year period (1993–2023; Figure S8), with, on average, 8.6 comparisons per study (Figure S9). Most studies (*n* = 80) and comparisons (*n* = 753) were conducted in temperate zones, followed by studies from tropical (38 studies), subtropical (16) and boreal zones (10), and only one study reported comparisons of multiple climate zones (Ferreira et al. 2019) (Table S5). We found large spatial gaps in data coverage in Africa and Asia (Figure 2a). The most common comparisons examined effects of plantations, harvest, and conversion in temperate zones (*n* = 313, 294, 117), conversion in the tropics and subtropics (*n* = 197, 70), and harvest in boreal zones (*n* = 74) (Figure S10). Most comparisons spanned both local and catchment scales (*n* = 583), whereas 407 focused on local and 245 on catchment scales only (Figure 2d). The most common trophic level and metric combinations were detritus decomposition (*n* = 334 comparisons), omnivore abundance (*n* = 175) and diversity (*n* = 159), and shredder abundance (*n* = 151).

### 3.2 Overall Effects of Changes in the Watershed Vegetation on Freshwater Detrital Food Webs

**FIGURE 3:**
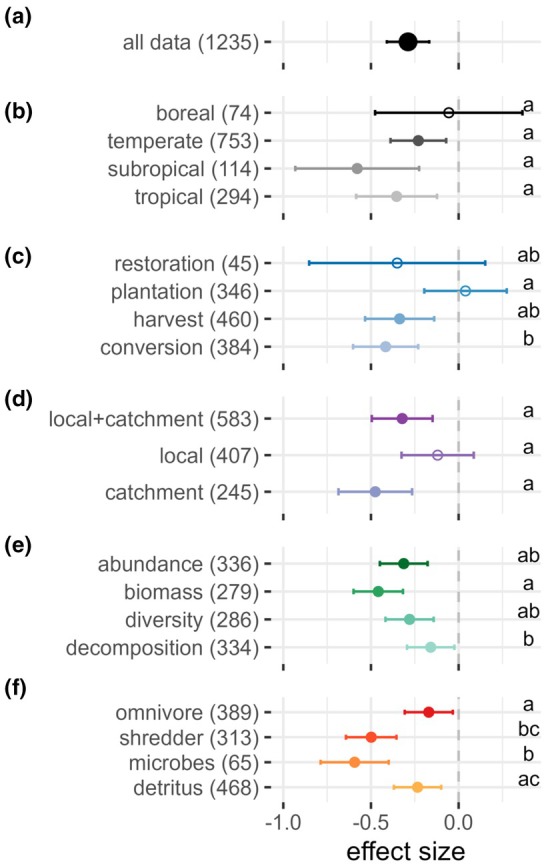
Impacts of human‐induced alterations in the watershed vegetation on freshwater detrital food webs. The global response (all data) is shown on the first row (a) and is separated by moderator level in the following rows for (b) climate, (c) type of vegetation change, (d) spatial scale of vegetation change, (e) metric, and (f) trophic level. The numbers in brackets after each moderator level represent the number of comparisons. For each moderator level, the circle represents the mean effect size (standardised mean differences Hedges' *g* between altered and reference vegetation conditions) with 95% confidence intervals (CIs) computed from the random effects model. Filled circles indicate statistically significant effects, whereas empty circles have CIs that cross the 0‐line and are thus statistically non‐significant. Negative effects sizes (Hedges' *g* < 0) indicate that the values in the altered conditions were lower compared to the reference conditions. Moderator levels sharing the same letter do not significantly differ as their CIs overlap.

The majority (58%) of individual effect sizes were negative, with a large portion (37%) being moderately to strongly negative (effect size < −0.5), which resulted in a grand mean effect size of −0.29 (95% CI = [−0.41, −0.17]; Figure 3a), indicating a significant reduction in the components and processes in the freshwater detrital food web as a consequence of direct human‐induced changes in the vegetation. Even after correction for publication bias, the bias‐robust estimate remained significantly negative (−0.06 [−0.11, −0.01]; Figure S1). There was, nevertheless, high between‐study heterogeneity contributing to total heterogeneity (*I* ^2^ = 93.83).

### 3.3 Effects of Main Moderators

The magnitude of the effect of human‐induced vegetation change on freshwater detrital food webs was driven by the type of vegetation change, spatial scale, trophic level, and metric, but not by climate (Figure 3, Table 1). While effects did not significantly differ across climate zones (Table 1), they were significantly negative in temperate, subtropical, and tropical climates but nonsignificant in boreal regions (Figure 3b). Land‐use conversions had stronger effects than plantations (Figure 3c). We found negative effects at local + catchment and catchment scales but not at local scales (Figure 3d). Among metrics, biomass was more affected than decomposition (Figure 3e). At the trophic level, effects were strongest for shredders (vs. omnivores) and microbes (vs. omnivores and detritus) (Figure 3f).

**TABLE 1:** Effects of moderators tested in the analyses based on the entire dataset (*n* = 1235 comparisons from 144 studies). Test for heterogeneity among levels within moderators (Q_M_), degrees of freedom (df) and *p* values (significant differences among moderator levels exist if *p*‐values < 0.05; see Figure 3) are shown. Rosenberg's fail‐safe number (*N*_fs_) was 6892, indicating the robustness (*N*_fs_ > 5 × *n* + 10, *n* = 1235) of our dataset to publication bias.

Moderator	Levels	Q_M_	df	*p*
All data		4225.18	1234	< 0.01
Climate	4 levels: boreal, temperate, subtropical, tropical	4.62	3	0.20
Type of vegetation change	4 levels: restoration, plantation, harvest, conversion	10.31	3	0.02
Spatial scale	3 levels: local+catchment, local, catchment	6.50	2	0.04
Metric	4 levels: abundance, biomass, diversity, decomposition	32.97	3	< 0.01
Trophic level	4 levels: omnivores, shredders, microbes, detritus	64.63	3	< 0.01

### 3.4 Effects of Moderators Within Trophic Levels

**FIGURE 4:**
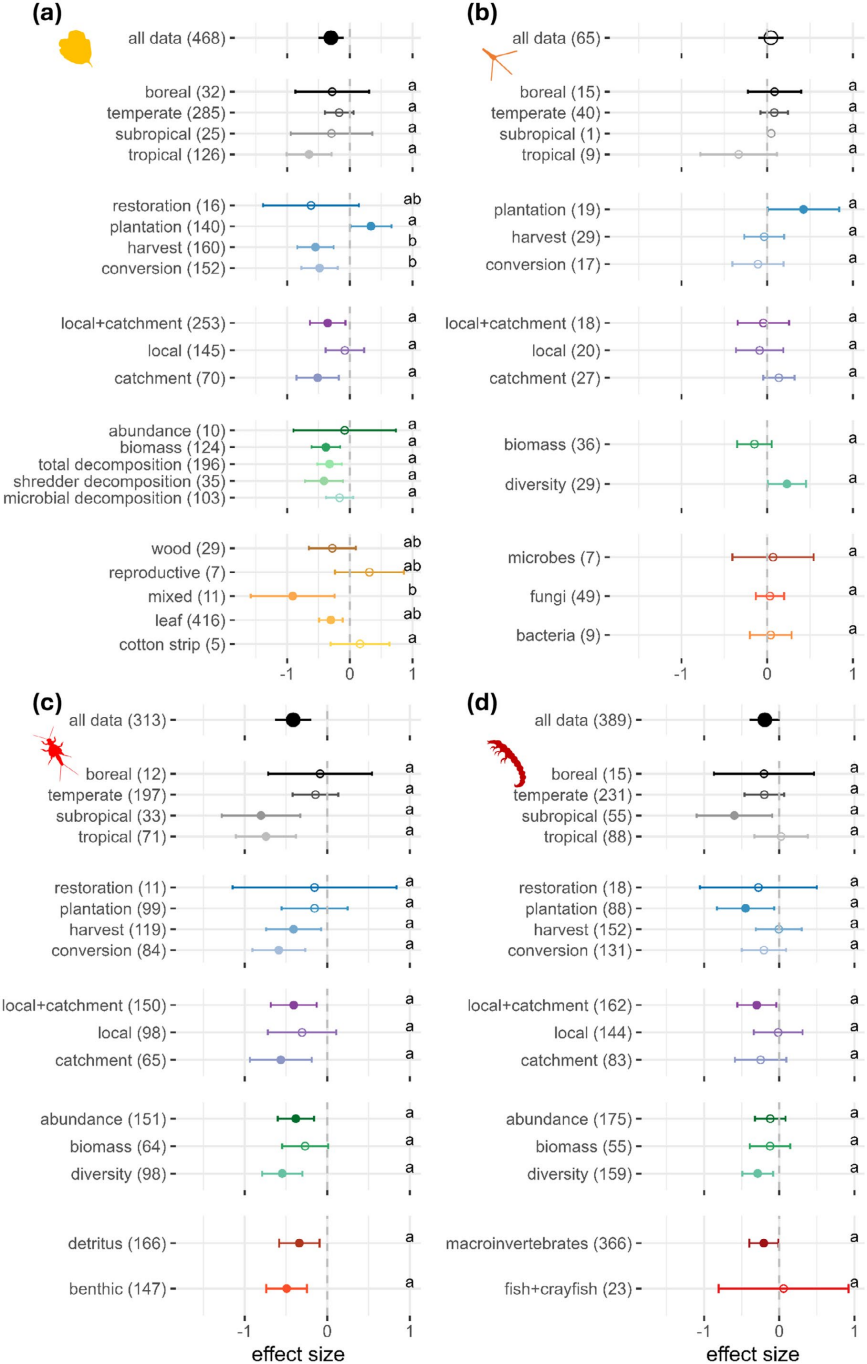
Moderator analyses separated for the subsets of each trophic level with (a) detritus, (b) microbes, (c) shredders, and (d) omnivores. For each moderator level, the circle represents the mean effect size (standardised mean differences Hedges' *g* between altered and reference vegetation conditions) with 95% confidence intervals (CIs) computed from the random effects model. Filled circles indicate statistically significant effects, whereas empty circles have CIs that cross the 0‐line and are thus statistically non‐significant. Negative effects sizes (Hedges' *g* < 0) indicate that the values in the altered conditions were lower compared to the reference conditions. Moderator levels sharing the same letter do not significantly differ as their CIs overlap.

To examine interactions between trophic levels and other moderators, we analyzed their effects across trophic levels and found varying responses to climate, type and spatial scale of vegetation change, and metric (Figure 4, Table 2, Table S2).

**TABLE 2:** Effects of moderators tested for different trophic levels. Test for heterogeneity among levels within moderators (Q_M_), degrees of freedom (df) and *p* values (significant differences among moderator levels exist if *p*‐values < 0.05; see Figure 4) are shown. Rosenberg's fail‐safe number (*N*_fs_) for the datasets of detritus (*N*_fs_ = 4675, *n* = 468) and shredders (*N*_fs_ = 4661, *n* = 313) indicate the robustness (*N*_fs_ > 5 × *n* + 10) of the datasets to publication bias; for omnivores (*N*_fs_ = 1936, *n* = 389), results need to be interpreted with caution as the *N*_fs_ is lower than the threshold for considering the dataset robust to publication bias (see also Figure S5); the effect of forest change on the microbial dataset was nonsignificant (Figure 4b) and hence there is no *N*_fs_.

Moderators	Levels	Q_M_	df	*p*
A. Detritus				
All		2046.00	467	< 0.01
Climate	4 levels: boreal, temperate, subtropical, tropical	5.27	3	0.15
Type of vegetation change	4 levels: restoration, plantation, harvest, conversion	21.77	3	< 0.01
Spatial scale	3 levels: local+catchment, local, catchment	5.16	2	0.08
Metric	5 levels: abundance, biomass, total decomposition, shredder decomposition, microbial decomposition	8.74	4	0.07
Type of detritus	5 levels: wood, reproductive, mixed, leaf, cotton strip	13.22	4	0.01
B. Microbes				
All		148.67	64	< 0.01
Climate	4 levels: boreal, temperate, subtropical, tropical	3.02	3	0.39
Type of vegetation change	3 levels: restoration, plantation, harvest	4.50	2	0.11
Spatial scale	3 levels: local+catchment, local, catchment	2.20	2	0.33
Metric	2 levels: biomass, diversity	8.43	1	< 0.01
Type of microbes	3 levels: microbes, fungi, bacteria	0.02	2	0.99
C. Shredders				
All		747.45	312	< 0.01
Climate	4 levels: boreal, temperate, subtropical, tropical	10.44	3	0.02
Type of vegetation change	4 levels: restoration, plantation, harvest, conversion	3.03	3	0.39
Spatial scale	3 levels: local+catchment, local, catchment	0.95	2	0.62
Metric	3 levels: abundance, biomass, diversity	5.34	2	0.07
Type of substrate	2 levels: detritus, benthic	1.47	1	0.23
D. Omnivores				
All		1180.53	388	< 0.01
Climate	4 levels: boreal, temperate, subtropical, tropical	3.91	3	0.27
Type of vegetation change	4 levels: restoration, plantation, harvest, conversion	3.74	3	0.27
Spatial scale	3 levels: local+catchment, local, catchment	1.81	2	0.40
Metric	3 levels: abundance, biomass, diversity	5.04	2	0.08
Type of omnivores	2 levels: macroinvertebrates, fish + crayfish	0.34	1	0.56

For detritus, type of vegetation change was the most influential moderator. Although harvest and land‐use conversion had the most negative effects, plantations had positive effects, and restoration showed no significant effect (Figure 4a, Table 2, Table S2). While type of detritus was another important moderator, climate, spatial scale, and metric had no overall influence (Figure 4a, Table 2, Table S2). However, there were statistically significant negative effects in tropical sites, at catchment and local + catchment scales, and in biomass. Decomposition showed significant negative responses for total and shredder‐mediated decomposition but not for microbial decomposition (Figure 4a).

For microbes, community metric was the only statistically significant moderator with positive effects of vegetation change on diversity and nonsignificant negative effects on biomass (Figure 4b, Table 2, Table S2).

For shredders, climate was the primary moderator, with significantly negative effects in the tropics and subtropics and nonsignificant negative effects in boreal and temperate zones (Figure 4c, Table 2, Table S2). Although type and spatial scale of vegetation change were not statistically significant moderators, harvest and land‐use conversion had negative effects, as did changes at catchment and local+catchment scales (Figure 4c). Metric showed no overall effect, although abundance and diversity declined significantly, whereas biomass remained unaffected (Figure 4c).

For omnivores, no moderator was statistically significant (Figure 4d, Table 2, Table S2). However, effects were negative in the subtropics, with plantations, at the local + catchment scale, for diversity and macroinvertebrates (Figure 4d).

### 3.5 Multitrophic Effects

**FIGURE 5:**
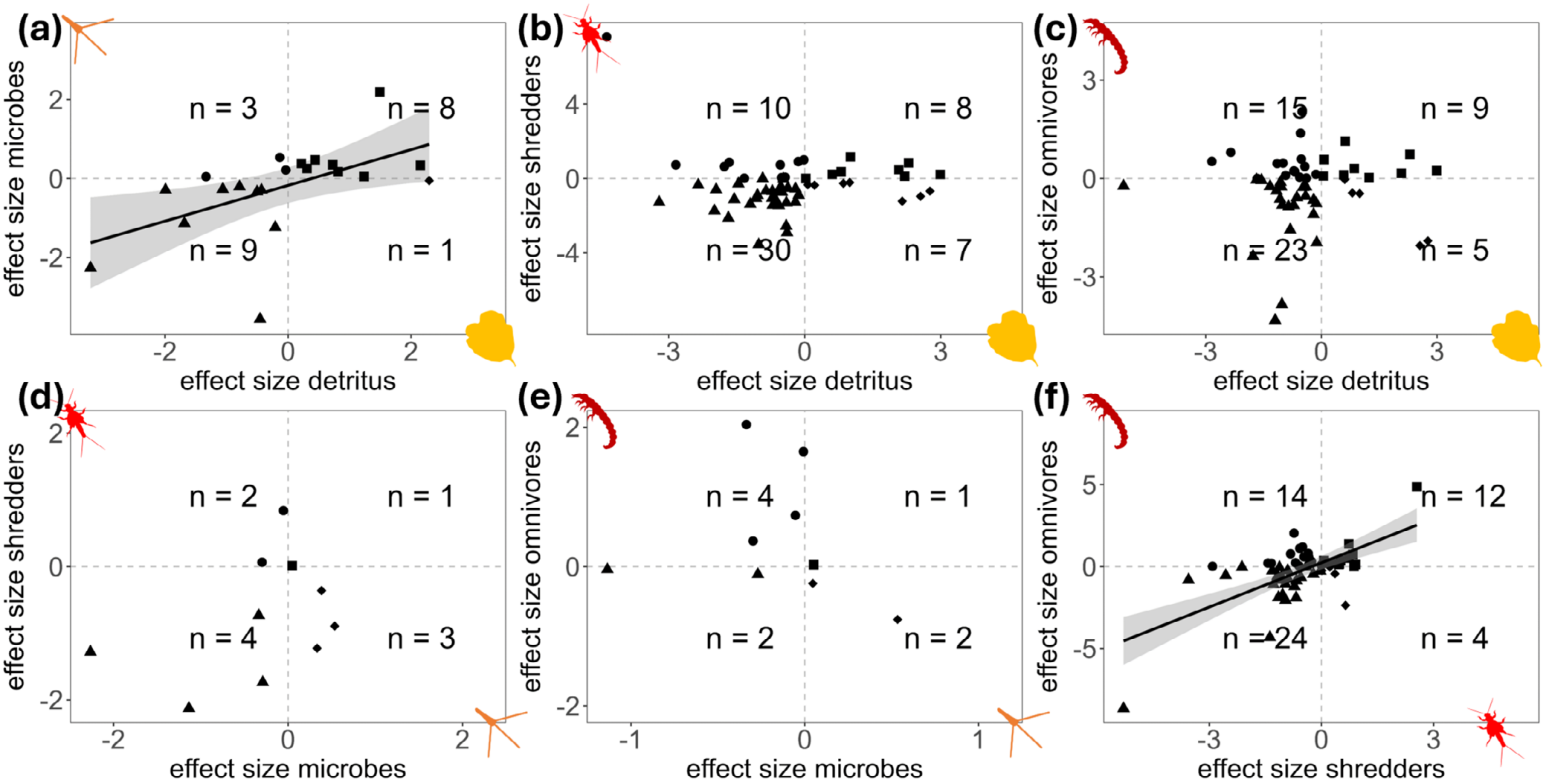
Quadrant graphs of median effect sizes of one trophic level against another in all possible combinations: (a) detritus‐microbes, (b) deritus‐shredders, (c) detritus‐omnivores, (d) microbes‐shredders, (e) microbes‐omnivores, and (f) shredders‐omnivores. Each point represents a study, and the symbols indicate the quadrant in which the effect sizes of the study lie in the *x*/*y*‐axes (triangles (−/−), circles (−/+), diamonds (+/−) and squares (+/+)). The quadrants are separated by a dashed grey line going through 0 on both axes. Negative effects sizes (standardised mean differences Hedges' *g* between altered and reference vegetation conditions < 0) indicate that the values in the altered conditions were lower compared to the reference conditions. For panels (a) and (f) we found statistically significant positive relationships indicated by the regression with the grey shaded area representing the confidence area around the linear regression.

Most studies reported simultaneous negative effects across trophic levels (Figure 5). Forty‐three percent reported negative effects for both detritus and microbes (Figure 5a), 55% for detritus and shredders (Figure 5b), and 44% for detritus and omnivores (Figure 5c). Likewise, 40% observed negative effects for microbes and shredders (Figure 5d), 22% for microbes and omnivores (Figure 5e), and 44% for shredders and omnivores (Figure 5f). We found statistically significant correlations between effect sizes of the trophic combinations: detritus–microbes (intercept = −0.18, slope = 0.45, SE = 0.16, rho = 0.66, *p* value < 0.01) and shredders–omnivores (intercept = 0.23, slope = 0.90, SE = 0.15, rho = 0.48, *p* value < 0.001).

### 3.6 Sensitivity Analyses

Repeating the analyses without estimated values (i.e., without values extracted from figures or imputed values) did not change our results and interpretations much (Figures S6 and S7, Tables S3 and S4). The overall response to land‐cover changes in the watershed on freshwater detrital food webs remained significantly negative (Figure S1, Figure S6). Only considering reported values did also not affect the effect significance or direction for climate, type of vegetation change, or metric. The only changes occurred in local effects that became significantly negative and effects on microbes that became nonsignificant (Figure S6, Table S3). For the individual trophic levels, the significance level changed only in 9 out of 72 cases, most often with significant effects becoming nonsignificant except for the negative effect of changes at local scales on shredders that became significant; whenever effect sizes changed in directionality, these changes never resulted in statistically significant effects (Figure S7).

## 4 Discussion

Our meta‐analysis revealed the widespread negative impacts of human‐induced land‐cover changes on freshwater detrital food webs. The variation in effect size across types and spatial scales of vegetation change, metrics, and trophic levels underscores the complexity of the cascading consequences of these impacts. Notably, different trophic levels responded to distinct moderators, yet studies reporting negative effects of one trophic level most often observed negative effects in others as well. This simultaneous decrease suggests that land‐cover changes often negatively affect multiple detrital food‐web components in concert, potentially destabilizing these trophic networks. These comprehensive insights reinforce the urgency of preserving naturally vegetated watersheds and riparian zones, and of integrating cross‐ecosystem considerations into land‐use and conservation strategies.

### 4.1 Overall Effect of Vegetation Change on Freshwater Detrital Food Webs

Freshwater detrital food webs consistently showed negative responses to human‐induced land‐cover changes. This robust evidence confirms that such changes can cascade across ecosystem boundaries, reducing biodiversity and disrupting ecosystem processes in adjacent ecosystems on a global scale (Allan 2004; Hanna et al. 2020; IPBES 2019; Tiegs et al. 2024). While our synthesis focused on overall detrital food web responses, the variability in effect sizes likely reflects not only changes in resource quantity but also differences in how land‐cover change affects the quality of basal resources. Vegetation change can alter multiple biotic and abiotic factors, leading to context‐dependent and potentially nonlinear effects on food webs (Campanyà‐Llovet et al. 2017; Frainer and McKie 2021; Jyväsjärvi et al. 2022; Turunen et al. 2017). Nevertheless, our findings not only reinforce but also expand and refine our current understanding of the global consequences of tree harvesting (Richardson and Béraud 2014), exotic tree plantations (Ferreira et al. 2016, 2019), and other land‐cover changes on freshwater biodiversity (Camana et al. 2024; Petsch et al. 2021) and ecosystem processes (Brauns et al. 2022; Tiegs et al. 2024). Despite the broad latitudinal coverage of our dataset, we observed a significant underrepresentation of studies from Africa and Asia, highlighting a geographical bias in the existing literature and a prioritization area for future research. Geographical bias may also arise from potential dissemination biases. Gray and non‐English literature could have potentially filled some of these gaps and created a more comprehensive picture (O'Dea et al. 2021) of our observed negative effects of land‐cover changes on freshwater detrital food webs.

### 4.2 Moderator Effects on Food Webs

As expected, we found negative effect sizes of land‐cover changes on freshwater detrital food webs, especially in the tropics and subtropics (da Costa et al. 2020; Ebling et al. 2024; Suga and Tanaka 2013; Valente‐Neto et al. 2015). Yet, the lack of significant effects in boreal regions suggests that variability in these responses may stem from factors beyond vegetation change alone or from qualitative responses, which we could not systematically include. In forest ecosystems, stand age appears to play a crucial role (Ishikawa et al. 2016), leading to hump‐shaped responses in adjacent detrital food webs, characterized by peak decomposition at intermediate stand ages and increasingly variable outcomes along the age gradient (Frainer and McKie 2021). Additionally, the effects of harvesting might also depend on whether the local riparian forest consisted of deciduous or coniferous species (Kominoski et al. 2013) or the width of riparian buffers (Jyväsjärvi et al. 2020).

While the overall effects of harvest and land‐use conversions on freshwater detrital food webs were negative, we found no statistically significant effects of plantations and restorations. The strong negative effects of land‐use conversions emerged likely due to the extensive alteration of riparian and catchment vegetation, which could have affected habitat structure, resource availability, and abiotic conditions simultaneously, ultimately reducing biodiversity and ecosystem functioning (Allan 2004; Camana et al. 2024; Mckie and Malmqvist 2009; Petsch et al. 2021). For plantations, overall effects were nonsignificant because of the opposing responses of different trophic levels, with a decrease for omnivores and increases for microbes and detritus. In contrast, the weak responses to restoration likely stem from differences in goals and approaches such as study design, reference conditions, and age of restoration. For example, some studies assessed the effects of replanting native riparian vegetation (Giling et al. 2015), whereas others focused on removing invasive species (McNeish et al. 2017). These findings highlight the context‐dependency of restoration outcomes in our dataset (Nilsson et al. 2015) and, along with the low number of studies, emphasize the need for more research on the effects of vegetation restoration on aquatic food webs using clear before‐after or control‐intervention designs (Bellmore et al. 2017; Naiman et al. 2012; Weber et al. 2018; see Loch et al. 2020 for a review on food web restoration). Since we excluded studies that did not assess stream food webs, riparian restoration studies that focused solely on terrestrial improvements were not included in our dataset. We argue that incorporating in‐stream effects of riparian restoration is important as it provides a more holistic understanding of restoration outcomes. Moreover, our findings reinforce the need for a broader research focus that goes beyond species‐level responses to consider multiple trophic levels, ecosystem processes, and entire interaction networks (Bellmore et al. 2017; Naiman et al. 2012; Nilsson et al. 2015; Palmer 2009). Regular, long‐term monitoring is essential to capture ecosystem dynamics and assess the full impact of restoration efforts (Palmer 2009; Weber et al. 2018).

With respect to the spatial scale of vegetation change, freshwater detrital food webs were most affected when land‐cover change occurred at catchment and local + catchment scales in line with previous studies (Ferreira et al. 2016). In contrast, the weaker effects at local scales may reflect the relatively lower magnitude of vegetation changes (e.g., riparian buffers might partially remain intact or replaced with exotic trees). Although such changes can alter local inputs of organic matter, they may not be as disruptive as large‐scale alterations at the catchment level, which can influence hydrology, water quality, and resource availability more extensively. Our findings also emphasize the importance of local intactness of riparian vegetation for many aquatic organisms, as demonstrated in numerous studies (e.g., Casotti et al. 2015; Encalada et al. 2010; Giling et al. 2015; Kominoski et al. 2011; Masese et al. 2014; Oester et al. 2023). Therefore, preserving or implementing natural riparian zones wide enough to buffer freshwater ecosystems from human activities at local and regional scales remains key to maintaining and protecting aquatic processes and biodiversity.

Our findings highlight that changes in watershed vegetation negatively affect both community structure and ecosystem processes within freshwater detrital food webs, as reflected by declines across all assessed metrics and trophic levels. The strongest declines in decomposition occurred when assessing total and shredder‐mediated decomposition, which are closely linked to the activity and feeding interactions of detritivores (Cummins et al. 1989; Elosegi et al. 2018; Ferreira et al. 2015; Gessner et al. 2010; Handa et al. 2014). In line with these results, we found the most negative overall effect sizes for microbes and shredders. These negative effects are not surprising, as these organism groups are known to be highly specialized and dependent on detritus (Cummins et al. 1989; Wallace and Webster 1996). Moreover, many shredders and other macroinvertebrate groups also depend on the terrestrial environment during their terrestrial adult stages, when they use local and upstream riparian vegetation for shelter, refuge, courtship, and mating (Reinhart and VandeVoort 2006; Vilela and Sanmartín‐Villar 2019; Yoshimura 2012). Hence, to protect these sensitive taxa, natural riparian vegetation is crucial (Cummins et al. 1989; Tolkkinen et al. 2020).

### 4.3 Moderator Effects on Individual Trophic Levels

Deconstructing the response to land‐cover changes in detrital food webs by trophic level revealed that each trophic level was shaped by distinct moderators. For detritus, the effects varied depending on the type of vegetation change: negative for harvested and converted vegetation but positive for plantations. These differences likely reflect shifts in quantity, quality, and timing of detritus inputs and its subsequent decomposition (Ferreira et al. 2019; Houghton et al. 2011; Larrañaga et al. 2023; Link et al. 2022; Mlambo et al. 2019; Tonin et al. 2017). However, faster decomposition does not always linearly scale with nor represent the ecological state of an ecosystem (Erdozain et al. 2018, 2021; Frainer et al. 2021; Hagen et al. 2006; Masese et al. 2014; Truchy et al. 2022). Plantations, for instance, might provide detritus that is more nutritious, palatable, or available for the detrital food web than detritus from the pristine vegetation (de Castro et al. 2018; Collier and Halliday 2000; Tank et al. 2010). While non‐native eucalyptus plantations often have negative impacts on detrital food webs (Ferreira et al. 2016, 2019), the direction of the effects of conifer (Martínez et al. 2013; Riipinen et al. 2009; Sakai et al. 2013), palm oil (Chellaiah and Yule 2018), citrus (Dézerald et al. 2014), coffee and rubber (Walsh et al. 2002), sugarcane (dos Santos et al. 2015), or banana plantations (Casotti et al. 2015; Kiffer Jr. et al. 2018) varies depending on the reference conditions, resource environment, and metrics considered.

Consumer responses also differed, with microbial and shredder communities influenced by distinct moderators. Shredders in tropical and subtropical regions, where deforestation and land‐cover changes are most severe, showed the strongest negative impacts. Given that tropical and subtropical freshwaters are global biodiversity hotspots but remain poorly understood in terms of ecosystem functioning, research efforts should prioritize these regions. Understanding the cross‐ecosystem consequences of ongoing land‐cover changes in these areas is essential for conservation and management strategies (Boyero et al. 2012; Encalada et al. 2010).

### 4.4 Multitrophic Effects

We observed negative effects due to land‐cover changes in watershed vegetation within a single trophic level of the detrital food web and often also simultaneously for multiple trophic levels. Although we may be missing studies reporting positive effects on omnivores, the datasets for other trophic groups were robust and suggest two possibilities: Either the land‐cover change was so severe that multiple trophic levels reacted negatively independently, or the decrease in one trophic level cascaded through the food web affecting other trophic levels (Barnes et al. 2017; Bruder et al. 2019; Knight et al. 2005; Ono et al. 2020). Regardless, shifts in the freshwater detrital food web—whether through community changes or alterations in trophic interactions and ecosystem processes—can have severe consequences for trophic pathways to higher order consumers in both aquatic and terrestrial environments (Albertson et al. 2018; Ballinger and Lake 2006; Nakano and Murakami 2001).

## 5 Conclusion

Our findings reveal the overall negative impact of land‐cover changes related to watershed vegetation on freshwater detrital food webs, with no indication that restoration measures were able to revert these, at least over the time and space covered by the respective studies. Moreover, while different trophic levels responded to distinct moderators, studies that reported negative effects on one trophic level often reported declines also in others. These findings thus highlight the urgent need for conserving and more effectively restoring watersheds. Effective management should integrate both terrestrial and freshwater biodiversity and functioning, using multiple metrics and considering various trophic levels when assessing the impacts of vegetation change. Hence, land‐cover management must account for cross‐ecosystem consequences at both local and catchment scales. Adopting holistic conservation strategies that account for ecological complexity, including multiple trophic levels, trophic dependencies, and ecosystem processes is crucial for preserving and restoring freshwater ecosystems in landscapes with increasing human pressure (Bellmore et al. 2017; Harrison et al. 2018; Naiman et al. 2012).

## Author Contributions

**Rebecca Oester:** conceptualization, data curation, formal analysis, investigation, methodology, project administration, resources, software, validation, visualization, writing – original draft, writing – review and editing. **François Keck:** conceptualization, formal analysis, investigation, methodology, software, validation, visualization, writing – review and editing. **Marcelo S. Moretti:** conceptualization, funding acquisition, project administration, resources, supervision, writing – review and editing. **Florian Altermatt:** conceptualization, funding acquisition, project administration, resources, supervision, writing – review and editing. **Andreas Bruder:** conceptualization, funding acquisition, project administration, resources, supervision, writing – review and editing. **Verónica Ferreira:** conceptualization, data curation, formal analysis, funding acquisition, investigation, methodology, project administration, resources, software, supervision, validation, writing – review and editing.

## Conflicts of Interest

The authors declare no conflicts of interest.

## Supporting information


**Data S1:** gcb70380‐sup‐0001‐Supinfo.pdf.

## Data Availability

The data that support the findings of this study are openly available on Dryad https://doi.org/10.5061/dryad.c866t1ghg and all code to reproduce the analysis can be found on Zenodo: https://doi.org/10.5281/zenodo.14289859.
