# Supplementary material for: A Global Synthesis on Land‐Cover Changes in Watersheds Shaping Freshwater Detrital Food Webs
Source: Glob Chang Biol. 2025 Aug 4;31(8):e70380. doi: 10.1111/gcb.70380 (PMC12319666; doi:10.1111/gcb.70380)
Supplement: Supplementary file 1 — Data S1: gcb70380‐sup‐0001‐Supinfo.pdf. [file GCB-31-e70380-s001.pdf]

Supplementary Material

A global synthesis on land-cover changes in watersheds shaping freshwater detrital food webs

List of authors: Rebecca Oester [1],[1,2,3,*,] François Keck [2,3], Marcelo S. Moretti [4], Florian Altermatt [2,3], Andreas Bruder [1], Verónica Ferreira [5]

Afiliations:
1 Institute of Microbiology, University of Applied Sciences and Arts of Southern Switzerland, via Flora Ruchat Roncati 15, CH-6850 Mendrisio, Switzerland

2 Department of Evolutionary Biology and Environmental Studies, University of Zurich, Winterthurerstr. 190, CH-8057 Zurich, Switzerland

3 Eawag: Swiss Federal Institute of Aquatic Science and Technology, Department of Aquatic Ecology, Überlandstr. 133, CH-8600 Dübendorf, Switzerland

4 Laboratory of Aquatic Insect Ecology, Universidade Vila Velha, Av. Comissário José Dantas de Melo 21, 29102-920 Vila Velha, ES, Brazil

5 MARE – Marine and Environmental Sciences Centre, ARNET – Aquatic Research Network, Department of Life Sciences, University of Coimbra, Calçada Martim de Freitas, 3000–456 Coimbra, Portugal

* Corresponding author: rebecca.oester@eawag.ch; +41 58 765 5312

[a] Present address: Animal Network Ecology, Department of Biology, University of Hamburg, Ohnhorststr. 18, 20148 Hamburg, Germany

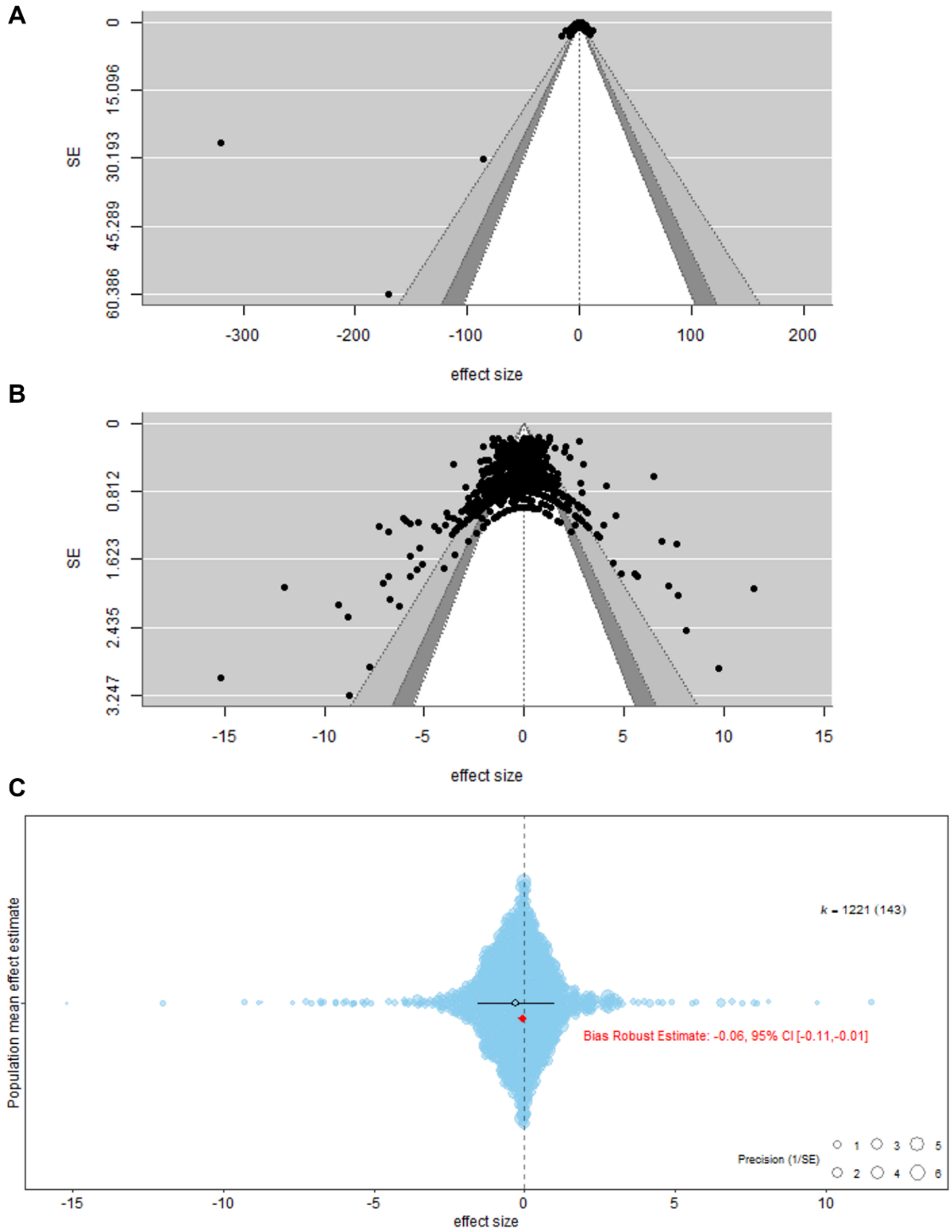

Figure S1. Funnel plots for the **whole dataset** with (A) and without (B) three outlier values with extreme effect sizes (all three outlier comparisons stem from shredder-mediated decomposition rates calculated as k_c/k_f from tropical leaves reported directly from the authors or in the text from the studies 19 and 84 (see Table S3 for whole reference)) and publication bias represented as an orchard plot of the whole dataset without outliers (C). This orchard plot illustrates the distribution of standardised mean differences (Hedges' g) from 1221 comparisons (from 143 studies) evaluating the population mean effect estimate. Each blue circle corresponds to an effect size, with the size of the circle reflecting the precision (1/SE) of the estimate. The black horizontal lines show the overall mean effect size with a 95 % (thick) and 99 % (thin) line as confidence intervals (CI). The bias robust estimate is highlighted in red, showing Hedges' g of -0.06 with a 95 % CI ranging from -0.11 to -0.01. This suggests a small but statistically significant negative effect, accounting for potential bias in the data and that there is no evidence of publication bias in our data because the interpretation of results does not change after accounting for potential bias in the data.

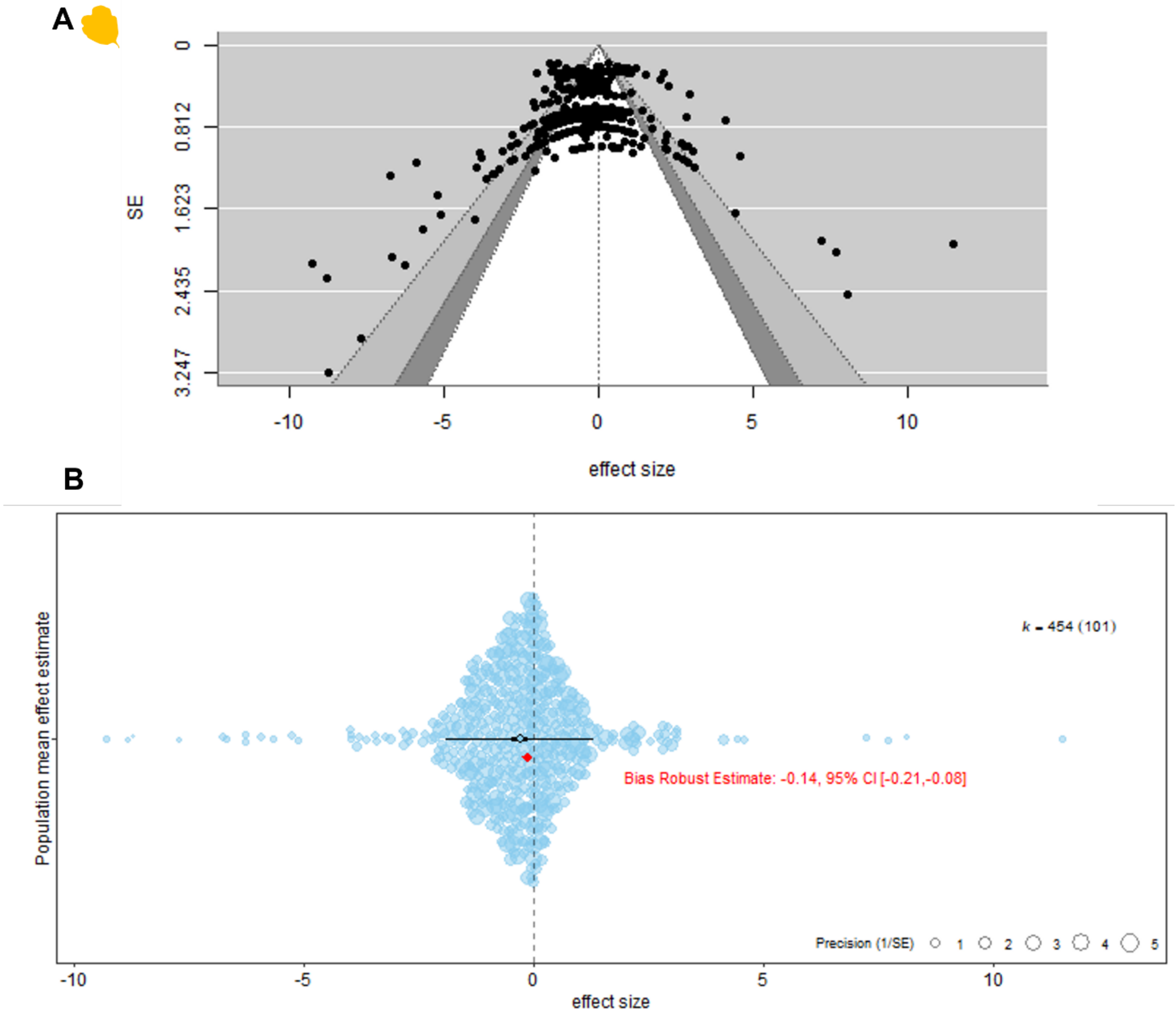

Figure S2. Funnel plots for the **detritus** (A) and publication bias represented as an orchard plot of this dataset (B). This orchard plot illustrates the distribution of standardised mean differences (Hedges' g) from 454 comparisons (from 101 studies) evaluating the population mean effect estimate. Each blue circle corresponds to an effect size, with the size of the circle reflecting the precision (1/SE) of the estimate. The black horizontal lines show the overall mean effect size with a 95 % (thick) and 99 % (thin) line as confidence intervals (CI). The bias robust estimate is highlighted in red, showing Hedges' g of -0.14 with a 95 % CI ranging from -0.21 to -0.08. This suggests a small but statistically significant negative effect, accounting for potential bias in the data and that there is no evidence of publication bias in our data because the interpretation of results does not change after accounting for potential bias in the data.

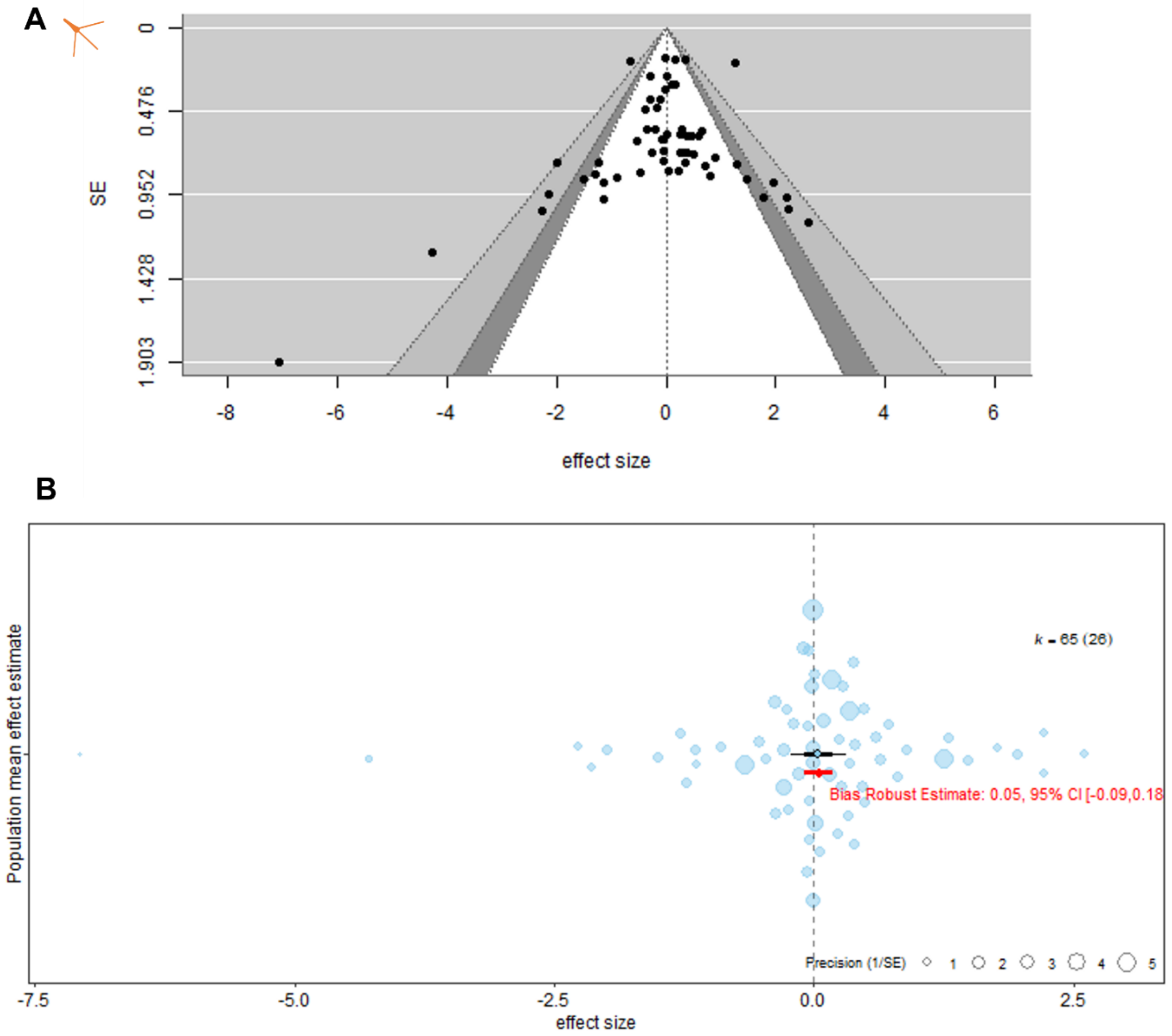

Figure S3. Funnel plots for the (A) and publication bias represented as an orchard plot of this dataset (B). This orchard plot illustrates the distribution of standardised mean differences (Hedges' g) from 65 comparisons (from 26 studies) evaluating the population mean effect estimate. Each blue circle corresponds to an effect size, with the size of the circle reflecting the precision (1/SE) of the estimate. The black horizontal lines show the overall mean effect size with a 95 % (thick) and 99 % (thin) line as confidence intervals (CI). The bias robust estimate is highlighted in red, showing Hedges' g of 0.05 with a 95 % CI ranging from -0.09 to 0.18. This suggests statistically non-significant effects, accounting for potential bias in the data and that there is no evidence of publication bias in our data because the interpretation of results does not change after accounting for potential bias in the data.

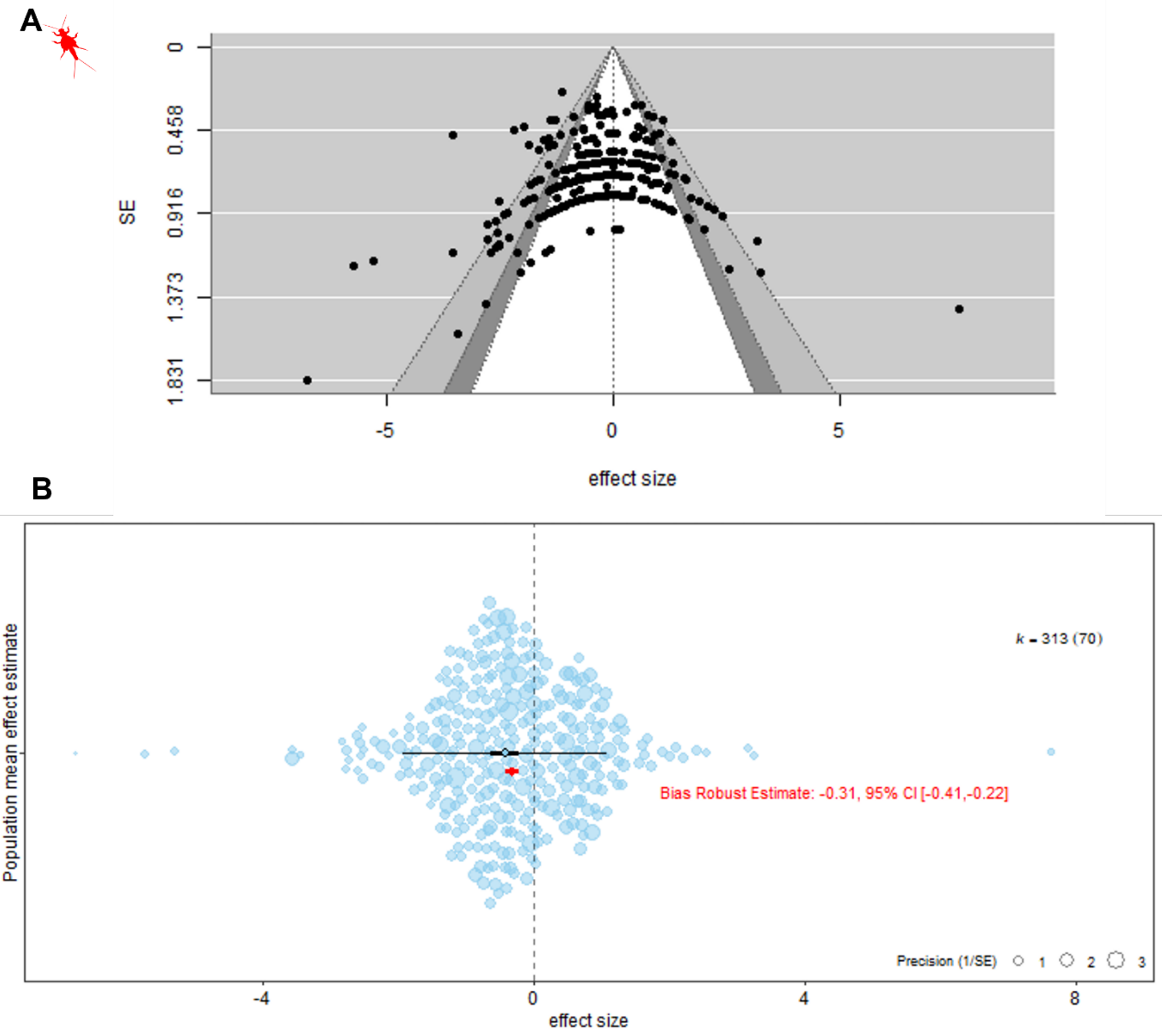

Figure S4. Funnel plots for the **shredders** (A) and publication bias represented as an orchard plot of this dataset (B). This orchard plot illustrates the distribution of standardised mean differences (Hedges' g) from 313 comparisons (from 70 studies) evaluating the population mean effect estimate. Each blue circle corresponds to an effect size, with the size of the circle reflecting the precision (1/SE) of the estimate. The black horizontal lines show the overall mean effect size with a 95 % (thick) and 99 % (thin) line as confidence intervals (CI). The bias robust estimate is highlighted in red, showing Hedges' g of -0.31 with a 95 % CI ranging from -0.41 to -0.22. This suggests statistically significant negative effect, accounting for potential bias in the data and that there is no evidence of publication bias in our data because the interpretation of results does not change after accounting for potential bias in the data.

.

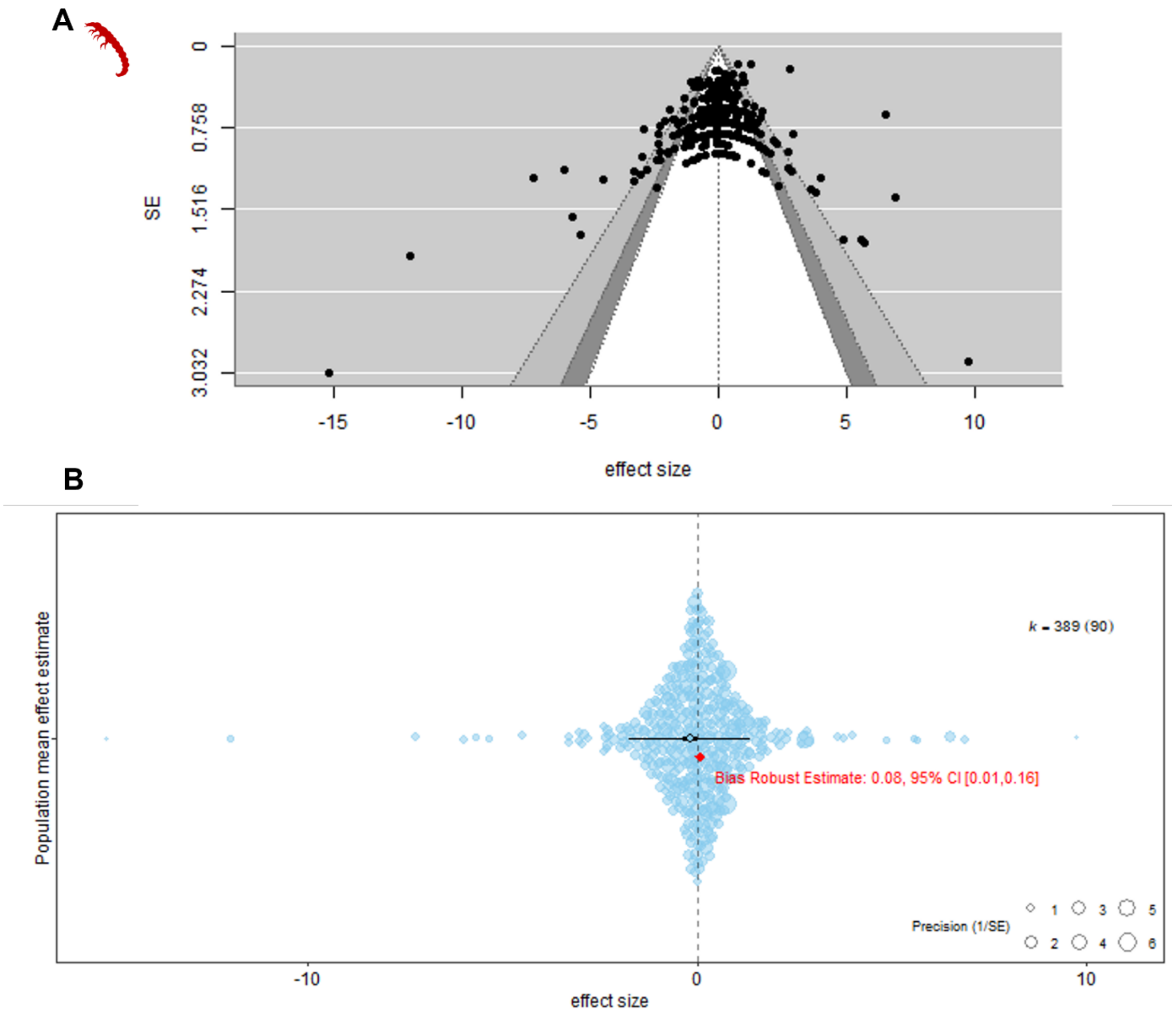

Figure S5. Funnel plots for the **omnivores** (A) and publication bias represented as an orchard plot of this dataset (B). This orchard plot illustrates the distribution of standardised mean differences (Hedges' g) from 389 comparisons (from 90 studies) evaluating the population mean effect estimate. Each blue circle corresponds to an effect size, with the size of the circle reflecting the precision (1/SE) of the estimate. The black horizontal lines show the overall mean effect size with a 95 % (thick) and 99 % (thin) line as confidence intervals (CI). The bias robust estimate is highlighted in red, showing a Hedges' g of 0.08 with a 95 % CI ranging from 0.01 to 0.16. This suggests that there might be bias in the data.

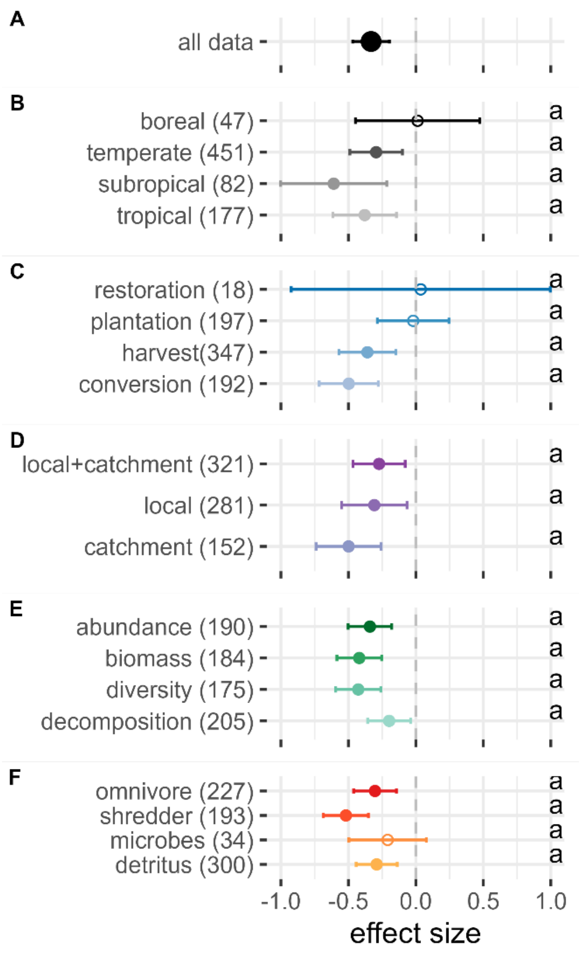

Figure S6. Impacts of human-induced alterations in the watershed vegetation on freshwater detrital food webs from only reported comparions (i.e., excluding imputed and estimated values). The global response (all data) is shown on the first row A) and is separated by moderators in the following rows for B) climate, C) type of vegetation change, D) spatial scale, E) metric and F) trophic level. The numbers in brackets after each moderator level represent the number of comparisons. For each moderator level, the circle represents the mean effect size with 95 % confidence intervals (CIs) computed from the random effects model. Filled circles indicate statistically significant effects, whereas empty circles have CIs that cross the 0-line and are thus statistically non-significant. Negative effects sizes (standardised mean differences Hedges' g between altered and reference vegetation conditions < 0) indicate that the values in the altered conditions were lower compared to the reference conditions. Groups sharing the same letter (e.g., 'a', 'ab', 'b') are not significantly different from each other as their CIs overlap.

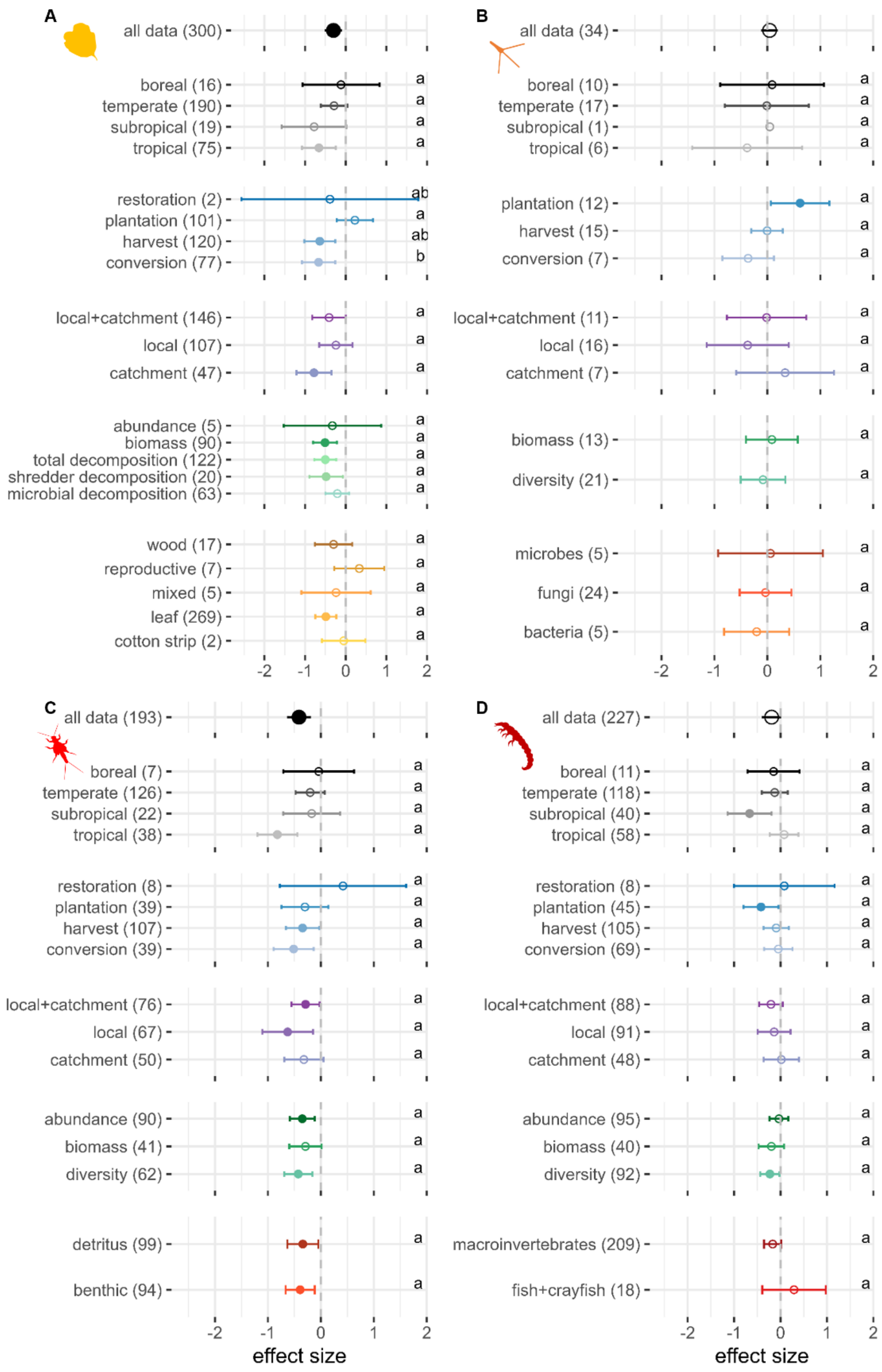

Figure S7. Moderator analysis separated for the subsets of each trophic level from only reported comparions (i.e., excluding imputed and estimated values) with A) detritus, B) microbes, C) shredders and D) omnivores. For each moderator level, the circle represents the mean effect size with 95 % confidence intervals (CIs) computed from the random effects model. Filled circles indicate statistically significant effects, whereas empty circles have CIs that cross the 0-line and are thus statistically non-significant. Negative effects sizes (standardised mean differences Hedges' g between altered and reference vegetation conditions < 0) indicate that the values in the altered conditions were lower compared to the reference conditions. Groups sharing the same letter (e.g., 'a', 'ab', 'b') are not significantly different from each other as their CIs overlap.

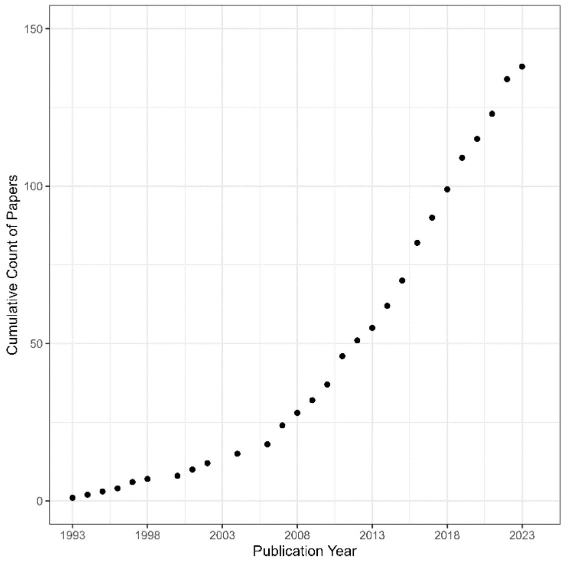

Figure S8. Cumulative number of publications (n = 144).

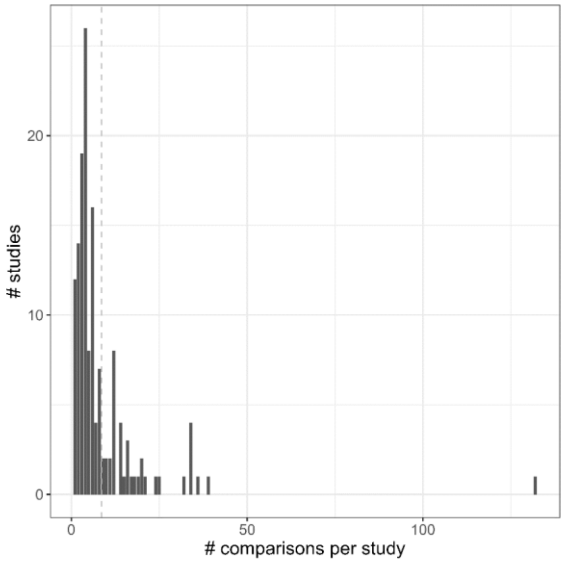

Figure S9. Histogram of data distribution with number of observations per study. The mean of 8.6 observations per study is indicated with the grey dashed line.

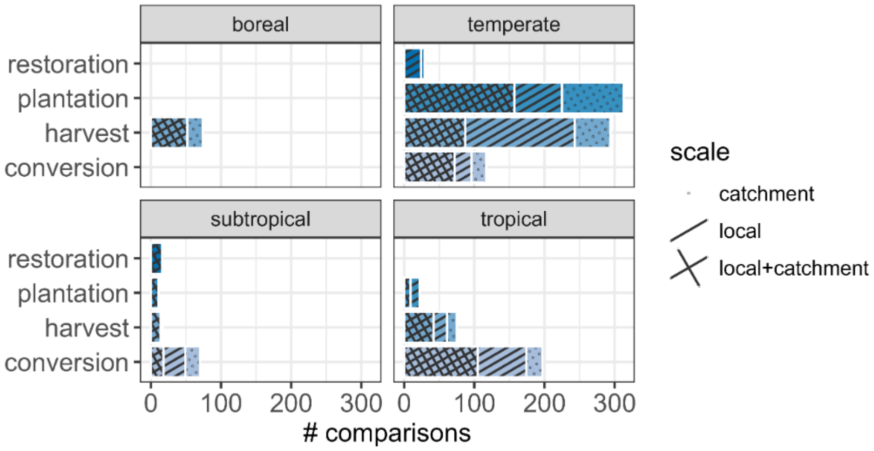

Figure S10. Distribution of comparisons between different climate (panels), types of vegetation change (bars) and spatial scales of vegetation change (pattern) with dotted bars indicating vegetation change at a catchment scale, striped bars indicating local changes and criss-crossed bars depicting local+catchment scale changes.

Table S1. Identity, levels and definitions of moderators used in this review.

Moderator	Levels	Definition
study ID	several	Full references are given in Table S5
year	continuous	Publication year
latitude	continuous	Latitude in decimal degrees
longitude	continuous	Longitude in decimal degrees
water body	several	Water body as described in the primary study, e.g., stream, river, bromeliad
climate	boreal	Boreal climate zone
	temperate	Temperate climate zone
	sub-tropical	Sub-tropical climate zone
	tropical	Tropical climate zone
scale	local	Forest change only occurs in the riparian area, not at the catchment level (e.g., Kennedy & Hobbie, 2004).
	catchment	Forest change occurs at the catchment level but not in the riparian area, where native vegetation is still present (e.g., Martínez et al., 2013).
	local+catchment	Forest change occurs at the catchment and riparian level (e.g., Lecerf et al., 2005).
type of vegetation change	restoration	Direct anthropogenic alteration through restoration measures aiming at restoring the native, natural riparian vegetation (e.g., Giling et al., 2016; McNeish et al., 2016)
	harvest	Direct anthropogenic alteration through harvesting or reducing the volume (e.g., logging or clear-cutting) of the natural riparian vegetation in the form of forestry management (e.g., Kominoski et al., 2011; Musetta-Lambert et al. 2017)
	land use conversion	Direct anthropogenic alteration through changes in the vegetation type (e.g., from forest to pasture) resulting in overall degradation, reduction in width and cover of natural riparian vegetation (e.g., Ferreira et al., 2015; Ono et al., 2020)
	plantation	Direct anthropogenic alteration through replacement of native forests by other types of riparian vegetation such as *Eucalyptus* or conifers (e.g., Hladyz et al., 2011, Martínez et al., 2013).
reference condition	several	Description of reference condition of the riparian vegetation as reported in the primary study
altered condition	several	Description of altered condition of the riparian vegetation as reported in the primary study
trophy	1	Detritus (e.g., leaf litter, wood)
	2	Microbial community (e.g., aquatic fungi, bacteria)
	3	Shredder community
	4	Omnivore community
type of detritus	leaf	Detritus in the form of leaves from riparian tree species (e.g., Oester et al. 2023)
	wood	Detritus in the form of wood, including bark, branches and twigs (e.g., Valente-Neto et al., 2015)
	mixed	Detritus in the form of leaves and wood mixed together (e.g., Ono et al., 2020)
	cotton strip	Detritus in the form of cotton strips (e.g., Mancuso et al., 2023)
	reproductive	Detritus in the form of reproductive material such as fruit, seeds, flowers, cones (e.g., Kadeka et al. 2021)
taxon of detritus	several	Detritus taxa as reported in the primary study
type of microbes	bacteria	Microbial community of bacteria
	fungi	Microbial community of fungi
	microbe	Microbial community of microbes (not specified or a mix of bacteria, fungi and algae)
type of consumers	macroinvertebrates	Macroinvertebrate community (i.e., insect larva, molluscs, small crustaceans, etc.)
	shredders	Detritivorous macroinvertebrates based on the functional guild of shredders (i.e., macroinvertebrate preferentially shredding on coarse organic matter)
	fish+crayfish	Detritivorous fish and crayfish community
type of substrate	CPOM	Location of sample collection based on CPOM (coarse particulate organic matter)
	benthic	Location of sample collection based on the benthic habitat
	water column	Location of sample collection based on the water column
taxon of substrate	several	Taxon name of the CPOM (e.g. species name of the leaf litter origin)
metric	a-diversity	α-diversity provided by the primary study (preferentially richness, but if not provided also other metrics were considered)
	abundance	Abundance of microbes or invertebrates provided in the primary study
	biomass	Biomass of microbes or invertebrates provided in the primary study
	Total decompositon	Total decomposition of detritus

	Shredder-mediated decomposition	Part of total decomposition mediated by shredders and omnivores
	Microbial decomposition	Part of total decomposition provided by microbes
unit	several	Unit of α-diversity, abundance, biomass or decomposition as reported in the primary study (the only exception was that decomposition reported as "% mass remaining" was converted to "% mass lost", by subtracting "%mass remaining" from 100 %, to be consistent with the other metrics such as decomposition rates and g mass lost)
data origin	provided	Data derived from either text, tables or authors directly
	estimated	Data derived from figures and extracted through WebPlotDigitizer
imputation	original	Standard deviation derived from study
	imputed	Standard deviation imputed based on similar studies (in terms of fw_category and metric) following Koricheva et al. (2013).

Table S2. Datasets, moderators and levels tested in the analyses, sample size (n), Rosenberg's fail-safe number (N_{fs}) (dataset is robust to publication bias if $N_{fs} > 5 \times n + 10$, with n= number of effect sizes), estimated mean effect size Hedges' g (standardized mean differences between altered and reference riparian vegetation conditions) with 95 % confidence intervals (CI) computed from the random effect model (negative effects sizes indicate that the values in the altered conditions were lower compared to the reference conditions), p-values (significant effects exist if $p < 0.05$), percentage of total variation that is due to between-study variation (I^2) and test for heterogeneity between levels within moderators (Q_M).

Data	Moderator	Levels	n	N_{fs}	Estimate [95% CI]	p-value	I^2 (%)
Detritus	Entire dataset		468	4675	-0.30 [-0.49,-0.12]	< 0.01	94.61
	Climate	Boreal	32		-0.28 [-0.87,0.31]	0.35	88.31
		Temperate	285		-0.17 [-0.40,0.06]	0.14	95.10
		Sub-Tropical	25		-0.29 [-0.94,0.36]	0.37	72.12
		Tropical	126		-0.65 [-1.01,-0.29]	< 0.01	93.08
	Metric	Abundance	10		-0.08 [-0.90,0.74]	0.84	72.89
		Biomass	124		-0.38 [-0.61,-0.15]	< 0.01	85.85
		Microbial decomposition	103		-0.16 [-0.38,0.05]	0.14	82.83
		Shredder-mediated decomposition	35		-0.41 [-0.72,-0.11]	< 0.01	51.82
		Total decomposition	196		-0.33 [-0.52,-0.13]	< 0.01	89.72
	Type	Leaf	416		-0.30 [-0.49,-0.11]	< 0.01	94.11
		Cotton strip	5		0.16 [-0.31,0.63]	0.50	36.77
		Mixed	11		-0.91 [-1.58,-0.24]	< 0.01	45.35
		Reproductive	7		0.31 [-0.24,0.86]	0.27	27.60
		Wood	29		-0.28 [-0.65,0.09]	0.14	83.99
	Vegetation Change	Conversion	152		-0.48 [-0.78,-0.19]	< 0.01	91.05
		Harvest	160		-0.55 [-0.84,-0.26]	< 0.01	93.19
		Plantation	140		0.34 [0.01,0.66]	0.04	95.81
		Restoration	16		-0.62 [-1.39,0.15]	0.11	78.50
	Scale	Catchment	70		-0.51 [-0.85,-0.18]	< 0.01	90.67
		Local	145		-0.08 [-0.39,0.23]	0.61	85.62
		Local+Catchment	253		-0.35 [-0.64,-0.07]	0.01	96.62
Microbes	Entire dataset		65		0.04 [-0.09,0.18]	0.54	37.23
	Climate	Boreal	15		0.09 [-0.22,0.40]	0.59	10.49
		Temperate	40		0.08 [-0.08,0.24]	0.32	42.25
		Sub-Tropical	1		0.05 [-1.57,1.66]	0.96	1.82
		Tropical	9		-0.33 [-0.78,0.11]	0.14	8.35
	Metric	Biomass	36		-0.15 [-0.35,0.05]	0.15	48.93
		Diversity	29		0.23 [0.01,0.45]	0.04	44.91
	Type	Microbe	7		0.07 [-0.41,0.54]	0.78	12.02
		Bacteria	9		0.04 [-0.20,0.28]	0.74	31.31
		Fungi	49		0.03 [-0.13,0.20]	0.71	33.26
	Vegetation Change	Conversion	17		-0.11 [-0.41,0.19]	0.48	68.64
		Harvest	29		-0.04 [-0.27,0.20]	0.77	55.50
		Plantation	19		0.42 [0.01,0.81]	0.05	21.88
	Scale	Catchment	27		0.14 [-0.05,0.32]	0.15	45.04
		Local	20		-0.09 [-0.37,0.19]	0.53	26.33
		Local+Catchment	18		-0.04 [-0.34,0.25]	0.77	10.36
Shredders	Entire dataset		313	4661	-0.41 [-0.62,-0.2]	< 0.01	88.54
	Climate	Boreal	12		-0.09 [-0.72,0.54]	0.79	76.27
		Temperate	197		-0.14 [-0.42,0.13]	0.3	89.02
		Sub-Tropical	33		-0.80 [-1.28,-0.33]	< 0.01	79.24
		Tropical	71		-0.74 [-1.11,-0.38]	< 0.01	84.32
	Metric	Abundance	151		-0.38 [-0.60,-0.16]	< 0.01	80.02
		Biomass	64		-0.27 [-0.55,0.01]	0.06	65.69
		Diversity	98		-0.55 [-0.79,-0.30]	< 0.01	70.94
	Type	Benthic	147		-0.49 [-0.74,-0.24]	< 0.01	82.48
		CPOM	166		-0.34 [-0.58,-0.09]	0.01	84.15
	Vegetation Change	Conversion	84		-0.59 [-0.91,-0.27]	< 0.01	82.34
		Harvest	119		-0.41 [-0.74,-0.07]	0.02	87.96
		Plantation	99		-0.15 [-0.55,0.25]	0.45	89.63
		Restoration	11		-0.15 [-1.15,0.84]	0.76	88.95
	Scale	Catchment	65		-0.56 [-0.94,-0.19]	< 0.01	84.79
		Local	98		-0.30 [-0.72,0.11]	0.15	88.57
		Local+Catchment	150		-0.41 [-0.69,-0.13]	< 0.01	87.22
Omnivores	Entire dataset		389	1936	-0.19 [-0.38,0.00]	0.04	69.32
	Climate	Boreal	15		-0.20 [-0.87,0.46]	0.55	80.27
		Temperate	231		-0.20 [-0.46,0.07]	0.14	92.26
		Sub-Tropical	55		-0.60 [-1.1,-0.09]	0.02	84.17
		Tropical	88		0.02 [-0.33,0.38]	0.90	88.68
	Metric	Abundance	175		-0.12 [-0.32,0.08]	0.25	79.08

	Biomass	55	-0.12 [-0.39,0.15]	0.37	62.05
	Diversity	159	-0.29 [-0.49,-0.08]	0.01	82.96
Type	Fish+crayfish	23	0.06 [-0.81,0.92]	0.90	90.00
	Macroinvertebrates	366	-0.21 [-0.40,-0.01]	0.04	90.34
Vegetation Change	Conversion	131	-0.20 [-0.50,0.09]	0.17	83.35
	Harvest	152	-0.01 [-0.31,0.30]	0.97	92.41
	Plantation	88	-0.45 [-0.83,-0.07]	0.02	89.47
	Restoration	18	-0.28 [-1.06,0.50]	0.48	88.50
Scale	Catchment	83	-0.25 [-0.59,0.09]	0.15	90.37
	Local	144	-0.02 [-0.34,0.31]	0.93	88.54
	Local+Catchment	162	-0.30 [-0.56,-0.04]	0.02	87.54

Table S3. Effects of moderators tested in the analyses based on the dataset with only reported comparions (i.e., excluding imputed and estimated values). Test for heterogeneity between levels within moderators (Q_M), degrees of freedom (df) and p-values (significant differences among moderator levels exist if p-values < 0.05; see Figure S6) are shown.

Moderator	Levels	Q_M	df	p-value
All data		2550.84	747	< 0.01
Climate	4 levels: boreal, temperate, subtropical, tropical	4.36	3	0.22
Type of vegetation change	4 levels: restoration, plantation, harvest, conversion	8.67	3	0.03
Spatial scale	3 levels: local+catchment, local, catchment	2.65	2	0.27
Metric	4 levels: abundance, biomass, diversity, decomposition	14.84	3	< 0.01
Trophic level	4 levels: omnivores, shredders, microbes, detritus	15.33	3	< 0.01

Table S4. Effects of moderators tested for different trophic levels for the subset of only reported comparisons (i.e., excluding imputed and estimated values). Test for heterogeneity between levels within moderators (Q_M), degrees of freedom (df) and p-values (significant differences among moderator levels exist if p-values < 0.05; see Figure 7) are shown.

Moderators	Levels	Q_M	df	p-value
A. Detritus				
All		1646.94	299	< 0.01
Climate	4 levels: boreal, temperate, subtropical, tropical	3.29	3	0.35
Type of vegetation change	4 levels: restoration, plantation, harvest, conversion	13.30	3	0.004
Spatial scale	3 levels: local+catchment, local, catchment	5.68	2	0.06
Metric	5 levels: abundance, biomass, total decomposition, shredder decomposition, microbial decomposition	12.65	4	0.01
Type of detritus	5 levels: wood, reproductive, mixed, leaf, cotton strip	11.39	4	0.02
B. Microbes				
All		64.18	33	< 0.01
Climate	4 levels: boreal, temperate, subtropical, tropical	0.48	3	0.92
Type of vegetation change	3 levels: restoration, plantation, harvest	6.92	2	0.03
Spatial scale	3 levels: local+catchment, local, catchment	1.34	2	0.51
Metric	2 levels: biomass, diversity	0.37	1	0.55
Type of microbes	3 levels: microbes, fungi, bacteria	0.53	2	0.77
C. Shredders				
All		383.28	192	< 0.01
Climate	4 levels: boreal, temperate, subtropical, tropical	8.37	3	0.04
Type of vegetation change	4 levels: restoration, plantation, harvest, conversion	2.33	3	0.51
Spatial scale	3 levels: local+catchment, local, catchment	1.48	2	0.48
Metric	3 levels: abundance, biomass, diversity	0.80	2	0.67
Type of substrate	2 levels: detritus, benthic	0.08	1	0.78
D. Omnivores				
All		447.74	226	< 0.01
Climate	4 levels: boreal, temperate, subtropical, tropical	6.69	3	0.08
Type of vegetation change	4 levels: restoration, plantation, harvest, conversion	2.89	3	0.41
Spatial scale	3 levels: local+catchment, local, catchment	0.93	2	0.63
Metric	3 levels: abundance, biomass, diversity	4.75	2	0.09
Type of omnivores	2 levels: macroinvertebrates, fish+crayfish	1.61	1	0.20

Table S5. Source, full reference, information on data extracted and excluded from the studies selected for inclusion, and main moderators.

Study ID	Source	Full reference	Data extracted Reference	Altered	Data excluded	Climate	Type of vegetation change	Scale of vegetation change	Trophic levels	Metrics
1	Abelho & Graça, 1996	Abelho, M., & Graça, M. A. S. (1996). Effects of eucalyptus afforestation on leaf litter dynamics and macroinvertebrate community structure of streams in Central Portugal. *Hydrobiologia*, 324(3), 195–204. https://doi.org/10.1007/BF00016391	Deciduous	Eucalyptus	Mixed land use	temperate	plantation	local	detritus, omnivore	biomass, decomposition, abundance, diversity
2	Aguiar et al., 2018	Aguiar, A. C. F., Neres-Lima, V., & Moulton, T. P. (2018). Relationships of shredders, leaf processing and organic matter along a canopy cover gradient in tropical streams. *Journal of Limnology*, 77(1), Article 1. https://doi.org/10.4081/jlimnol.2017.1684	VAL	CAP	Other intermediate sites	tropical	conversion	local	detritus, shredder	biomass, decomposition
3	Albertson et al., 2018	Albertson, L. K., Ouellet, V., & Daniels, M. D. (2018). Impacts of stream riparian buffer land use on water temperature and food availability for fish. *Journal of Freshwater Ecology*, 33(1), 195–210. https://doi.org/10.1080/02705060.2017.1422558	ChesLen	Taylor Run	Other intermediate sites	temperate	conversion	local	omnivore	abundance, biomass, diversity
4	Astudillo et al., 2016	Astudillo, M. R., Novelo-Gutiérrez, R., Vázquez, G., García-Franco, J. G., & Ramírez, A. (2016). Relationships between land cover, riparian vegetation, stream characteristics, and aquatic insects in cloud forest streams, Mexico. *Hydrobiologia*, 768(1), 167–181. https://doi.org/10.1007/s10750-015-2545-1	Chivizcoyo	El Chorrito	Other intermediate sites	tropical	harvest	local+catchment	omnivore, shredder	abundance, diversity
5	Bacca et al., 2023	Bacca, J. C., Rampanelli Cararo, E., Alves Lima-Rezende, C., Tavares Martins, R., Macedo-Reis, L. E., Dal Magro, J., & De Souza Rezende, R. (2023). Land-use effects on aquatic macroinvertebrate diversity in subtropical highland grasslands streams. *Limnetica*, 42(2), 1. https://doi.org/10.23818/limn.42.16	Chivizcoyo	GNRV; Silviculture	Grassland with riparian buffer	subtropical	harvest, conversion	local	detritus, omnivore	biomass, abundance, diversity
6	Bärlocher et al., 2010	Bärlocher, F., Helson, J. E., & Williams, D. D. (2010). Aquatic hyphomycete communities across a land-use gradient of Panamanian streams. *Fundamental and Applied Limnology*, 177(3), 209–221. https://doi.org/10.1127/1863-9135/2010/0177-0209	Pristine	Rural	Urban	tropical	conversion	local	microbes	diversity
8	Barrios et al., 2022	Barrios, M., Burwood, M., Kröger, A., Calvo, C., Ríos-Touma, B., & Teixeira-de-Mello, F. (2022). Riparian cover buffers the effects of abiotic and biotic predictors of leaf decomposition in subtropical streams. *Aquatic Sciences*, 84(4), 55. https://doi.org/10.1007/s00027-022-00886-z	RFS	OCS		subtropical	conversion	local	omnivore, shredder, detritus	abundance, decomposition
9	Basaguren & Pozo, 1994	Basaguren, A., & Pozo, J. (1994). Leaf litter processing of alder and eucalyptus in the Agüera stream system (Northern Spain). II: Macroinvertebrates associated. Leaf*Archiv für Hidrobiologie*, 132(1), 57–68.	S1	S7	SA, S9	temperate	plantation	catchment	detritus	decomposition
10	Benfield et al., 2001	Benfield, E. F., Webster, J. R., Tank, J. L., & Hutchens, J. J. (2001). Long-Term Patterns in Leaf Breakdown in	Big Hurricane	Big Hurricane	Hugh White Creek and later	temperate	harvest	catchment	detritus	decomposition

		Streams in Response to Watershed Logging. *International Review of Hydrobiology*, 86(4–5), 467–474. https://doi.org/10.1002/1522-2632(200107)86:4/5<467::AID-IROH467>3.0.CO;2-1	Branch before logging	Branche during logging	measurements with different leaf litter species					
11	Bertaso et al., 2015	Bertaso, T. R. N., Spies, M. R., Kotzian, C. B., & Flores, M. L. T. (2015). Effects of forest conversion on the assemblages' structure of aquatic insects in subtropical regions. *Revista Brasileira de Entomologia*, 59(1), 43–49. https://doi.org/10.1016/j.rbe.2015.02.005	Forested	Converted		subtropical	conversion	catchment	omnivore, shredder	abundance, diversity
13	Burrows et al., 2014	Burrows, R. M., Magierowski, R. H., Fellman, J. B., Clapcott, J. E., Munks, S. A., Roberts, S., Davies, P. E., & Barmuta, L. A. (2014). Variation in stream organic matter processing among years and benthic habitats in response to forest clearfelling. *Forest Ecology and Management*, *327*, 136–147. https://doi.org/10.1016/j.foreco.2014.04.041	D, E, F, H, in April 2008	A and B in April 2008	Other dates, fine sediment sites	temperate	harvest	local+catchment	detritus	decomposition
14	Burrows et al., 2012	Burrows, R. M., Magierowski, R. H., Fellman, J. B., & Barmuta, L. A. (2012). Woody debris input and function in old-growth and clear-felled headwater streams. *Forest Ecology and Management*, 286, 73–80. https://doi.org/10.1016/j.foreco.2012.08.038	Old growth forest	CBS-affected		temperate	harvest	local+catchment	detritus	decomposition, abundance
15	Burwood et al., 2021	Burwood, M., Clemente, J., Meerhoff, M., Iglesias, C., Goyenola, G., Fosalba, C., Pacheco, J. P., & Teixeira De Mello, F. (2021). Macroinvertebrate communities and macrophyte decomposition could be affected by land use intensification in subtropical lowland streams. *Limnetica*, 40(2), 343–357. https://doi.org/10.23818/limn.40.23	Extensive	Intensive		subtropical	conversion	local+catchment	detritus, omnivore, shredder	decomposition, abundance, biomass, diversity
16	Bylak et al., 2022	Bylak, A., Kukuła, K., Ortyl, B., Hałoń, E., Demczyk, A., Janora-Hołyszko, K., Maternia, J., Szczurowski, Ł., & Ziobro, J. (2022). Small stream catchments in a developing city context: The importance of land cover changes on the ecological status of streams and the possibilities for providing ecosystem services. *Science of the Total Environment*, 815, 151974. https://doi.org/10.1016/j.scitotenv.2021.151974	1L	3L	2L	temperate	conversion	local+catchment	omnivore	diversity
17	Carroll & Jackson, 2009	Carroll, D., & Jackson, G. R. (2009). Observed relationships between urbanization and riparian cover, shredder abundance, and stream leaf litter standing crops. *Fundamental and Applied Limnology*, 213–225. https://doi.org/10.1127/1863-9135/2008/0173-0213	Most covered	Least covered	Urban	temperate	conversion	local+catchment	shredder, detritus	abundance, diversity, biomass
18	Casas et al., 2013	Casas, J. J., Larrañaga, A., Menéndez, M., Pozo, J., Basaguren, A., Martínez, A., Pérez, J., González, J. M., Mollá, S., Casado, C., Descals, E., Roblas, N., López-González, J. A., & Valenzuela, J. L. (2013). Leaf litter decomposition of native and introduced tree species of contrasting quality in headwater streams: How does the regional setting matter? *Science of the Total Environment*, 458–460, 197–208. https://doi.org/10.1016/j.scitotenv.2013.04.004	CC	SN	CLC, SG	temperate	plantation	local+catchment	shredder, detritus	abundance, decomposition, biomass

19	Casotti et al., 2015	Casotti, C. G., Kiffer, W. P., Costa, L. C., Rangel, J. V., Casagrande, L. C., & Moretti, M. S. (2015). Assessing the importance of riparian zones conservation for leaf decomposition in streams. *Natureza & Conservação*, 13(2), 178–182. https://doi.org/10.1016/j.ncon.2015.11.011	Banana	Norte	Luxemburgo and Macucuo	tropical	plantation	local	detritus, omnivore, shredder	decomposition, diversity, abundance
21	de Mello Cionek et al., 2021	de Mello Cionek, V., Fogaça, F. N. O., Moulton, T. P., Pazianoto, L. H. R., Landgraf, G. O., & Benedito, E. (2021). Influence of leaf miners and environmental quality on litter breakdown in tropical headwater streams. *Hydrobiologia*, 848(6), 1311–1331. https://doi.org/10.1007/s10750-021-04529-6	21	13; 24		tropical	conversion	local+catchment	detritus, microbes	decomposition, biomass
22	Collier et al., 2004	Collier, K. J., Smith, B. J., & Halliday, N. J. (2004). Colonization and use of pine wood versus native wood in New Zealand plantation forest streams: Implications for riparian management. *Aquatic Conservation: Marine and Freshwater Ecosystems*, 14(2), 179–199. https://doi.org/10.1002/aqc.599	PE4	PE2		temperate	harvest	catchment	microbes, omnivore	biomass, diversity, abundance
23	Collier & Halliday, 2000	Collier, K.J. & Halliday, J.N. (2000). Macroinvertebrate-wood associations during decay of plantation pine in New Zealand pumice-bed streams: stable habitat or trophic subsidy? J. North Am. Benthol. Soc., 19, 94–111.	13	2	Other sites	temperate	restoration	catchment	omnivore	diversity, abundance
24	Cordero–Rivera et al., 2017	Cordero–Rivera, A., Álvarez, A. M., & Álvarez, M. (2017). Eucalypt plantations reduce the diversity of macroinvertebrates in small forested streams. *Animal Biodiversity and Conservation*, 40(1), Article 1. https://doi.org/10.32800/abc.2017.40.0087	Least Eucalyptus cover	Most Eucalyptus cover	Other sites	temperate	plantation	catchment	omnivore	diversity
25	Cornejo et al., 2020	Cornejo, A., Pérez, J., López-Rojo, N., Tonin, A. M., Rovira, D., Checa, B., Jaramillo, N., Correa, K., Villarreal, A., Villarreal, V., García, G., Pérez, E., Ríos González, T. A., Aguirre, Y., Correa-Araneda, F., & Boyero, L. (2020). Agriculture impairs stream ecosystem functioning in a tropical catchment. *Science of the Total Environment*, 745, 140950. https://doi.org/10.1016/j.scitotenv.2020.140950	PA	BA	AA	tropical	conversion	local+catchment	detritus, omnivore, shredder	abundance, decomposition, diversity, biomass
26	Chellaiah & Yule, 2019	Chellaiah, D., & Yule, C. M. (2019). Litter quality influences bacterial communities more strongly than changes in riparian buffer quality in oil palm streams. *Aquatic Microbial Ecology*, 83(2), 167–179. https://doi.org/10.3354/ame01909	NF	OPNB	OPF, OPOP	tropical	plantation	local+catchment	detritus, microbes	decomposition, diversity
27	Chellaiah & Yule, 2018	Chellaiah, D., & Yule, C. M. (2018). Litter decomposition is driven by microbes and is more influenced by litter quality than environmental conditions in oil palm streams with different riparian types. *Aquatic Sciences*, 80(4), 43. https://doi.org/10.1007/s00027-018-0595-y	NF	OPNB	OPF, OPOP	tropical	plantation	local+catchment	detritus	decomposition
28	da Costa et al., 2020	da Costa, I. D., Petry, A. C., & Mazzoni, R. (2020). Fish assemblages respond to forest cover in small Amazonian basins. *Limnologica*, 81, 125757. https://doi.org/10.1016/j.limno.2020.125757	Forested	Deforested		tropical	harvest	catchment	omnivore, detritus	diversity, abundance

29	Lemes da Silva et al., 2020	Lemes da Silva, A. L., Lemes, W. P., Andriotti, J., Petrucio, M. M., & Feio, M. J. (2020). Recent land-use changes affect stream ecosystem processes in a subtropical island in Brazil. *Austral Ecology*, 45(5), 644–658. https://doi.org/10.1111/aec.12879	Forested	Rural	Urban	subtropical	conversion	catchment	detritus, shredder, omnivore	decomposition, abundance
30	Danger & Robson, 2004	Danger, A. R., & Robson, B. J. (2004). The effects of land use on leaf-litter processing by macroinvertebrates in an Australian temperate coastal stream. *Aquatic Sciences*, 66(3), 296–304. https://doi.org/10.1007/s00027-004-0718-5	Forested	Pasture	Boundary	temperate	conversion	local+catchment	detritus, shredder	decomposition, abundance
31	Daoust et al., 2019	Daoust, K., Kreutzweiser, D. P., Guo, J., Creed, I. F., & Sibley, P. K. (2019). Climate-influenced catchment hydrology overrides forest management effects on stream benthic macroinvertebrates in a northern hardwood forest. *Forest Ecology and Management*, 452, 117540. https://doi.org/10.1016/j.foreco.2019.117540	Forested	Harvested		temperate	harvest	catchment	omnivore	diversity
33	de Nadaï-Monoury et al., 2014	de Nadaï-Monoury, E., Gilbert, F., & Lecerf, A. (2014). Forest canopy cover determines invertebrate diversity and ecosystem process rates in depositional zones of headwater streams. *Freshwater Biology*, 59(7), 1532–1545. https://doi.org/10.1111/fwb.12364	Forested	Logged		temperate	harvest	local+catchment	detritus, omnivore, shredder	decomposition, diversity, abundance
34	Delong & Brusven, 1994	Delong, M. D., & Brusven, M. A. (1994). Allochthonous input of organic matter from different riparian habitats of an agriculturally impacted stream. *Environmental Management*, 18(1), 59–71. https://doi.org/10.1007/BF02393750	1,6	3,7	Other sites	temperate	conversion	local+catchment	detritus	biomass
35	Dézerald et al., 2014	Dézerald, O., Talaga, S., Leroy, C., Carrias, J.-F., Corbara, B., Dejean, A., & Céréghino, R. (2014). Environmental determinants of macroinvertebrate diversity in small water bodies: Insights from tank-bromeliads. *Hydrobiologia*, 723(1), 77–86. https://doi.org/10.1007/s10750-013-1464-2	Primary forest	Citrus plantation	Other sites	tropical	plantation	local	omnivore, shredder	diversity
36	Díez et al., 2002	Díez, J., Elosegi, A., Chauvet, E., & Pozo, J. (2002). Breakdown of wood in the Agüera stream. *Freshwater Biology*, 47(11), 2205–2215. https://doi.org/10.1046/j.1365-2427.2002.00965.x	D1	E1	Other sites	temperate	plantation	local+catchment	detritus, microbes	decomposition, biomass
37	dos Santos et al., 2015	dos Santos, F. B., Ferreira, F. C., & Esteves, K. E. (2015). Assessing the importance of the riparian zone for stream fish communities in a sugarcane dominated landscape (Piracicaba River Basin, Southeast Brazil). *Environmental Biology of Fishes*, 98(8), 1895–1912. https://doi.org/10.1007/s10641-015-0406-4	Forest	Sugarcane		subtropical	conversion	local+catchment	omnivore	abundance, diversity, biomass
38	Dudgeon, 2006	Dudgeon, D. (2006). The impacts of human disturbance on stream benthic invertebrates and their drift in North Sulawesi, Indonesia. *Freshwater Biology*, 51(9), 1710–1729. https://doi.org/10.1111/j.1365-2427.2006.01596.x	WS	AS	Other sites	tropical	conversion	local	omnivore	diversity, abundance
39	Dudley et al., 2021	Dudley, M. P., Solomon, K., Wenger, S., Jackson, C. R., Freeman, M., Elliott, K. J., Miniat, C. F., & Pringle, C. M. (2021). Do crayfish affect stream ecosystem response to	Cut+Burn PreYR 2014	Cut+Burn Burn YR 2016	Other sites and timepoints	temperate	restoration	local	omnivore, detritus	abundance, decomposition

		riparian vegetation removal? *Freshwater Biology*, 66(7), 1423–1435. https://doi.org/10.1111/fwb.13728								
40	Ebling et al., 2024	Ebling, L. A., Pastore, B. L., Biasi, C., Hepp, L. U., & Restello, R. M. (2024). Does environmental variability in Atlantic Forest streams affect aquatic hyphomycete and invertebrate assemblages associated with leaf litter? *Hydrobiologia*, 851(7), 1761–1777. https://doi.org/10.1007/s10750-023-05415-z	S3	S4	Other sites	subtropical	conversion	catchment	detritus, microbes, omnivore, shredder	decomposition, diversity, abundance
41	Elosegi et al., 2018	Elosegi, A., Nicolás, A., & Richardson, J. S. (2018). Priming of leaf litter decomposition by algae seems of minor importance in natural streams during autumn. *PLOS ONE*, 13(9), e0200180. https://doi.org/10.1371/journal.pone.0200180	Closed canopy; Forest	Open canopy; Agriculture		temperate	conversion	local, catchment	detritus	decomposition
42	Elosegi et al., 2007	Elosegi, A., Díez, J., & Pozo, J. (2007). Contribution of dead wood to the carbon flux in forested streams. *Earth Surface Processes and Landforms*, 32(8), 1219–1228. https://doi.org/10.1002/esp.1549	Salderrey	Jergueron		temperate	plantation	catchment	detritus	decomposition
43	Ely & Wallace, 2010	Ely, D. T., & Wallace, J. B. (2010). Long-term functional group recovery of lotic macroinvertebrates from logging disturbance. *Canadian Journal of Fisheries and Aquatic Sciences*, 67(7), 1126–1134. https://doi.org/10.1139/F10-045	Reference	Disturbed		temperate	harvest	catchment	shredder, omnivore, detritus	abundance, biomass
44	Emilson et al., 2016	Emilson, C. E., Kreutzweiser, D. P., Gunn, J. M., & Mykytczuk, N. C. S. (2016). Effects of land use on the structure and function of leaf-litter microbial communities in boreal streams. *Freshwater Biology*, 61(7), 1049–1061. https://doi.org/10.1111/fwb.12765	Undisturbed	Logged	Sites with other types of disturbances	boreal	harvest	catchment	detritus, microbes	decomposition, biomass, diversity
45	Encalada et al., 2010	Encalada, A. C., Calles, J., Ferreira, V., Canhoto, C. M., & Graça, M. A. S. (2010). Riparian land use and the relationship between the benthos and litter decomposition in tropical montane streams. *Freshwater Biology*, 55(8), 1719–1733. https://doi.org/10.1111/j.1365-2427.2010.02406.x	Forest	Pasture		tropical	conversion	local	detritus, microbes, omnivore, shredder	decomposition, biomass, diversity, abundance
46	Erdozain et al., 2021	Erdozain, M., Kidd, K. A., Emilson, E. J. S., Capell, S. S., Luu, T., Kreutzweiser, D. P., & Gray, M. A. (2021). Forest management impacts on stream integrity at varying intensities and spatial scales: Do biological effects accumulate spatially? *Science of the Total Environment*, 763, 144043. https://doi.org/10.1016/j.scitotenv.2020.144043	NBR	NBI		temperate	harvest	local+catchment	omnivore, shredder, detritus	abundance, diversity, decomposition
47	Erdozain et al., 2018	Erdozain, M., Kidd, K., Kreutzweiser, D., & Sibley, P. (2018). Linking stream ecosystem integrity to catchment and reach conditions in an intensively managed forest landscape. *Ecosphere*, 9(5), e02278. https://doi.org/10.1002/ecs2.2278	Reference	Harvested		temperate	harvest	local+catchment	detritus, omnivore, shredder	decomposition, abundance, diversity
49	Espinoza-Toledo et al., 2021	Espinoza-Toledo, A., Mendoza-Carranza, M., Castillo, M. M., Barba-Macías, E., & Capps, K. A. (2021). Taxonomic and functional responses of macroinvertebrates to riparian forest conversion in tropical streams. *Science of the Total*	Forest	Agriculture		tropical	conversion	local+catchment	detritus, omnivore	biomass, abundance, diversity

		Environment, 757, 143972. https://doi.org/10.1016/j.scitotenv.2020.143972								
50	Evangelista et al., 2014	Evangelista, C., Boiche, A., Lecerf, A., & Cucherousset, J. (2014). Ecological opportunities and intraspecific competition alter trophic niche specialization in an opportunistic stream predator. *Journal of Animal Ecology*, 83(5), 1025–1034. https://doi.org/10.1111/1365-2656.12208	Most covered	Least covered	Other sites	temperate	conversion	local	omnivore, shredder	biomass
51	Fargen et al., 2015	Fargen, C., Emery, S. M., & Carreiro, M. M. (2015). Influence of *Lonicera maackii* invasion on leaf litter decomposition and macroinvertebrate communities in an urban stream. *Natural Areas Journal*, 35(3), 392–403. https://doi.org/10.3375/043.035.0303	Removed	Invaded		temperate	restoration	local	detritus, shredder, omnivore	decomposition, abundance
52	Fasching et al., 2020	Fasching, C., Akotoye, C., Bižić, M., Fonvielle, J., Ionescu, D., Mathavarajah, S., Zoccarato, L., Walsh, D. A., Grossart, H.-P., & Xenopoulos, M. A. (2020). Linking stream microbial community functional genes to dissolved organic matter and inorganic nutrients. *Limnology and Oceanography*, 65(S1), S71–S87. https://doi.org/10.1002/lno.11356	Forest	Agriculture		temperate	conversion	local+catchment	microbes	diversity
53	Ferreira et al., 2019	Ferreira, V., Boyero, L., Calvo, C., Correa, F., Figueroa, R., Gonçalves, J. F., Goyenola, G., Graça, M. A. S., Hepp, L. U., Kariuki, S., López-Rodríguez, A., Mazzeo, N., M'Erimba, C., Monroy, S., Peil, A., Pozo, J., Rezende, R., & Teixeira-de-Mello, F. (2019). A global assessment of the effects of *Eucalyptus* plantations on stream ecosystem functioning. *Ecosystems*, 22(3), 629–642. https://doi.org/10.1007/s10021-018-0292-7	Forest	Plantation		temperate, tropical	plantation	local	detritus	decomposition
54	Ferreira et al., 2017	Ferreira, V., Faustino, H., Raposeiro, P. M., & Gonçalves, V. (2017). Replacement of native forests by conifer plantations affects fungal decomposer community structure but not litter decomposition in Atlantic island streams. *Forest Ecology and Management*, 389, 323–330. https://doi.org/10.1016/j.foreco.2017.01.004	Forest	Plantation		temperate	plantation	local	detritus, microbes	decomposition, diversity
55	Ferreira et al., 2015	Ferreira, V., Larrañaga, A., Gulis, V., Basaguren, A., Elosegi, A., Graça, M. A. S., & Pozo, J. (2015). The effects of eucalypt plantations on plant litter decomposition and macroinvertebrate communities in Iberian streams. *Forest Ecology and Management*, 335, 129–138. https://doi.org/10.1016/j.foreco.2014.09.013	Forest	Plantation		temperate	plantation	local	detritus, omnivore, shredder	decomposition, diversity, abundance
56	Ferreira et al., 2015	Ferreira, W. R., Ligeiro, R., Macedo, D. R., Hughes, R. M., Kaufmann, P. R., Oliveira, L. G., & Callisto, M. (2015). Is the diet of a typical shredder related to the physical habitat of headwater streams in the Brazilian Cerrado? *Annales de Limnologie*, 51(2), 115–127. https://doi.org/10.1051/limn/2015004	Upper Araguari River Basin	Upper Sao Francisco River Basin		tropical	conversion	local+catchment	omnivore, shredder	abundance, diversity
57	Ferreira et al., 2006	Ferreira, V., Elosegi, A., Gulis, V., Pozo, J., & Graça, M. A. S. (2006). Eucalyptus plantations affect fungal communities associated with leaf-litter decomposition in	Forest	Plantation		temperate	plantation	local	microbes	biomass, diversity

		Iberian streams. *Archiv für Hydrobiologie*, 166, 467–490. https://doi.org/10.1127/0003-9136/2006/0166-0467								
59	Valente-Neto et al., 2015	Valente-Neto, F., Koroiva, R., Fonseca-Gessner, A. A., & Roque, F. de O. (2015). The effect of riparian deforestation on macroinvertebrates associated with submerged woody debris. *Aquatic Ecology*, *49*(1), 115–125. https://doi.org/10.1007/s10452-015-9510-y	Forest	Pasture	Agriculture	tropical	conversion	local+catchment	omnivore	abundance, diversity
60	Four et al., 2017	Four, B., Arce, E., Danger, M., Gaillard, J., Thomas, M., & Banas, D. (2017). Catchment land use-dependent effects of barrage fishponds on the functioning of headwater streams. *Environmental Science and Pollution Research*, 24(6), 5452–5468. https://doi.org/10.1007/s11356-016-8273-x	Forest	Agriculture	Downstream of fish pond sites	temperate	conversion	local+catchment	detritus, microbes, shredder, omnivore	decomposition, biomass, abundance, diversity
61	Fu et al., 2016	Fu, L., Jiang, Y., Ding, J., Liu, Q., Peng, Q.-Z., & Kang, M.-Y. (2016). Impacts of land use and environmental factors on macroinvertebrate functional feeding groups in the Dongjiang River basin, southeast China. *Journal of Freshwater Ecology*, 31(1), 21–35. https://doi.org/10.1080/02705060.2015.1017847	Forest	Agriculture		subtropical	conversion	catchment	shredder	abundance, diversity
62	Fugère et al., 2018	Fugère, V., Jacobsen, D., Finestone, E. H., & Chapman, L. J. (2018). Ecosystem structure and function of afrotropical streams with contrasting land use. *Freshwater Biology*, 63(12), 1498–1513. https://doi.org/10.1111/fwb.13178	Forest	Farmland		tropical	conversion	local+catchment	detritus, shredder	decomposition, biomass, abundance
63	Giling et al., 2015	Giling, D. P., Nally, R. M., & Thompson, R. M. (2015). How sensitive are invertebrates to riparian-zone replanting in stream ecosystems? *Marine and Freshwater Research*, 67(10), 1500–1511. https://doi.org/10.1071/MF14360	Replanted	Untreated	Reference	subtropical	restoration	local+catchment	omnivore, shredder	abundance, diversity
64	Godoy et al., 2016	Godoy, B. S., Simião-Ferreira, J., Lodi, S., & Oliveira, L. G. (2016). Functional process zones characterizing aquatic insect communities in streams of the Brazilian Cerrado. *Neotropical Entomology*, 45(2), 159–169. https://doi.org/10.1007/s13744-015-0352-z	FPZ3	FPZ2	Other sites	tropical	harvest	local	omnivore, shredder	diversity, abundance
66	Goodman et al., 2006	Goodman, K. J., Hershey, A. E., & Fortino, K. (2006). The effect of forest type on benthic macroinvertebrate structure and ecological function in a pine plantation in the North Carolina Piedmont. *Hydrobiologia*, 559(1), 305–318. https://doi.org/10.1007/s10750-005-0990-y	Natural hardwood forest	Clea-cut; Pine forestry		temperate	harvest, plantation	local+catchment	detritus, omnivore	biomass, decomposition, diversity
67	Goss et al., 2014	Goss, C. W., Goebel, P. C., & Sullivan, S. M. P. (2014). Shifts in attributes along agriculture-forest transitions of two streams in central Ohio, USA. *Agriculture, Ecosystems & Environment*, 197, 106–117. https://doi.org/10.1016/j.agee.2014.07.026	Inside forest	Outside forest		temperate	conversion	local	shredder, detritus	abundance, decomposition
68	Göthe et al., 2009	Göthe, E., Lepori, F., & Malmqvist, B. (2009). Forestry affects food webs in northern Swedish coastal streams. *Fundamental and Applied Limnology*, 175(4), 281–294. https://doi.org/10.1127/1863-9135/2009/0175-0281	Old-growth forest	Clear-cut forest		boreal	harvest	local+catchment	omnivore, detritus, shredder	biomass, abundance, diversity
69	Guevara et al., 2018	Guevara, G., Godoy, R., & Franco, M. (2018). Linking riparian forest harvest to benthic macroinvertebrate communities in Andean headwater streams in southern	Unthinned forest	Thinned forest		tropical	harvest	local+catchment	detritus, shredder, omnivore	biomass, decomposition, abundance, diversity

		Chile. *Limnologica*, 68, 105–114. https://doi.org/10.1016/j.limno.2017.07.007								
70	Guevara et al., 2015	Guevara, G., Godoy, R., Boeckx, P., Jara, C., & Oyarzún, C. (2015). Effects of riparian forest management on Chilean mountain in-stream characteristics. *Ecohydrology & Hydrobiology*, 15(3), 160–170. https://doi.org/10.1016/j.ecohyd.2015.07.003	Forest	Harvested		tropical	harvest	catchment	detritus	biomass, decomposition
71	Hagen et al., 2006	Hagen, E. M., Webster, J. R., & Benfield, E. F. (2006). Are leaf breakdown rates a useful measure of stream integrity along an agricultural landuse gradient? *Journal of the North American Benthological Society*, 25(2), 330–343. https://doi.org/10.1899/0887-3593(2006)25[330:ALBRAU]2.0.CO;2	Forest	Agriculture	Intermediate sites	temperate	conversion	local+catchment	detritus, omnivore, shredder	decomposition, abundance, diversity
72	Hebert et al., 2023	Hebert, T. A., Kuehn, K. A., & Halvorson, H. M. (2023). Land use differentially alters microbial interactions and detritivore feeding during leaf decomposition in headwater streams. *Freshwater Biology*, 68(8), 1386–1399. https://doi.org/10.1111/fwb.14111	Forest	Agriculture	Sites with nutrient additions	temperate	conversion	catchment	microbes, detritus	biomass, decomposition
73	Hisabae et al., 2011	Hisabae, M., Sone, S., & Inoue, M. (2011). Breakdown and macroinvertebrate colonization of needle and leaf litter in conifer plantation streams in Shikoku, southwestern Japan. *Journal of Forest Research*, 16(2), 108–115. https://doi.org/10.1007/s10310-010-0210-0	Mixed deciduous forest	Conifer plantations		temperate	plantation	local	detritus, omnivore	decomposition, abundance
74	Hladyz et al., 2010	Hladyz, S., Tiegs, S. D., Gessner, M. O., Giller, P. S., Rîşnoveanu, G., Preda, E., Nistorescu, M., Schindler, M., & Woodward, G. (2010). Leaf-litter breakdown in pasture and deciduous woodland streams: A comparison among three European regions. *Freshwater Biology*, 55(9), 1916–1929. https://doi.org/10.1111/j.1365-2427.2010.02426.x	Woodland	Pasture		temperate	conversion	local+catchment	detritus	decomposition
75	Houghton et al., 2011	Houghton, D. C., Berry, E. A., Gilchrist, A., Thompson, J., & Nussbaum, M. A. (2011). Biological changes along the continuum of an agricultural stream: Influence of a small terrestrial preserve and use of adult caddisflies in biomonitoring. *Journal of Freshwater Ecology*, 26(3), 381–397. https://doi.org/10.1080/02705060.2011.563513	Site 4	Site 5	Other sites	temperate	conversion	local	omnivore	abundance, diversity
76	Huryn et al., 2002	Huryn, A. D., Huryn, V. M. B., Arbuckle, C. J., & Tsomides, L. (2002). Catchment land-use, macroinvertebrates and detritus processing in headwater streams: Taxonomic richness versus function. *Freshwater Biology*, 47(3), 401–415. https://doi.org/10.1046/j.1365-2427.2002.00812.x	Forested	Agriculture	Urban and Wetland sites	temperate	conversion	local+catchment	detritus, omnivore, shredder	decomposition, diversity, biomass
77	Illyová et al., 2011	Illyová, M., Beracko, P., & Krno, I. (2011). Influence of land use on hyporheos in catchment streams of the Velka Fatra Mts. *Biologia*, 66(2), 320–327. https://doi.org/10.2478/s11756-011-0018-1	Forest	Agriculture		temperate	conversion	catchment	omnivore	abundance
79	Iñiguez-Armijos et al., 2018	Iñiguez-Armijos, C., Hampel, H., & Breuer, L. (2018). Land-use effects on structural and functional composition of benthic and leaf-associated macroinvertebrates in four	Forest	Pasture		tropical	conversion	local+catchment	omnivore, shredder	abundance, diversity

		Andean streams. *Aquatic Ecology*, 52(1), 77–92. https://doi.org/10.1007/s10452-017-9646-z								
80	Iñiguez-Armijos et al., 2016	Iñiguez-Armijos, C., Rausche, S., Cueva, A., Sánchez-Rodríguez, A., Espinosa, C., & Breuer, L. (2016). Shifts in leaf litter breakdown along a forest–pasture–urban gradient in Andean streams. *Ecology and Evolution*, 6(14), 4849–4865. https://doi.org/10.1002/ece3.2257	Forest	Pasture	Urban	tropical	conversion	local	detritus, microbes	decomposition, biomass
81	Zúñiga-Sarango et al., 2020	Zúñiga-Sarango, W., Gaona, F. P., Reyes-Castillo, V., & Iñiguez-Armijos, C. (2020). Disrupting the biodiversity–ecosystem function relationship: Response of shredders and leaf breakdown to urbanization in Andean streams. *Frontiers in Ecology and Evolution*, 8. https://doi.org/10.3389/fevo.2020.592404	Forest	Pasture	Urban and mixed sites	tropical	conversion	local+catchment	shredder, detritus	abundance, diversity, decomposition
82	Inoue et al., 2012	Inoue, M., Shinotou, S., Maruo, Y., & Miyake, Y. (2012). Input, retention, and invertebrate colonization of allochthonous litter in streams bordered by deciduous broadleaved forest, a conifer plantation, and a clear-cut site in southwestern Japan. *Limnology*, 13(2), 207–219. https://doi.org/10.1007/s10201-011-0369-x	Forest	Plantation, Clear-cut		temperate	plantation, harvest	local+catchment	detritus, omnivore, shredder	biomass, abundance
83	Ishikawa et al., 2016	Ishikawa, N. F., Togashi, H., Kato, Y., Yoshimura, M., Kohmatsu, Y., Yoshimizu, C., Ogawa, N. O., Ohte, N., Tokuchi, N., Ohkouchi, N., & Tayasu, I. (2016). Terrestrial-aquatic linkage in stream food webs along a forest chronosequence: Multi-isotopic evidence. *Ecology*, 97(5), 1146–1158. https://doi.org/10.1890/15-1133.1	KU	S34	other intermediate sites	subtropical	harvest	local+catchment	omnivore, shredder	abundance, diversity, biomass
84	Jinggut et al., 2012	Jinggut, T., Yule, C. M., & Boyero, L. (2012). Stream ecosystem integrity is impaired by logging and shifting agriculture in a global megadiversity center (Sarawak, Borneo). *Science of the Total Environment*, 437, 83–90. https://doi.org/10.1016/j.scitotenv.2012.07.062	Pristine	Farmed, Logged		tropical	conversion, harvest	local	detritus, shredder	decomposition, abundance, diversity, biomass
85	Jyväsjärvi et al., 2020	Jyväsjärvi, J., Koivunen, I., & Muotka, T. (2020). Does the buffer width matter: Testing the effectiveness of forest certificates in the protection of headwater stream ecosystems. *Forest Ecology and Management*, 478, 118532. https://doi.org/10.1016/j.foreco.2020.118532	Reference	Narrow Buffer	Wide Buffer	boreal	harvest	local+catchment	detritus, omnivore	biomass, decomposition, diversity
86	Kadeka et al., 2021	Kadeka, E. C., Masese, F. O., Lusega, D. M., Sitati, A., Kondowe, B. N., & Chirwa, E. R. (2021). No Difference in instream decomposition among upland agricultural and forested streams in Kenya. *Frontiers in Environmental Science*, 9. https://doi.org/10.3389/fenvs.2021.794525	Forest	Agriculture		tropical	conversion	catchment	detritus, omnivore, shredder	biomass, abundance, diversity, decomposition
87	Kaylor & Warren, 2018	Kaylor, M. J., & Warren, D. R. (2018). Canopy closure after four decades of postlogging riparian forest regeneration reduces cutthroat trout biomass in headwater streams through bottom-up pathways. *Canadian Journal of Fisheries and Aquatic Sciences*, 75(4), 513–524. https://doi.org/10.1139/cjfas-2016-0519	Forest	Harvested		temperate	harvest	local	detritus	biomass
88	Kiffer Jr et al., 2018	Kiffer Jr, W., Giuberti, T., Victor Serpa, K., Mendes, F., & Moretti, M. (2018). Do changes in riparian zones affect periphyton growth and invertebrate colonization on rocky	Pau Amarelo	Luxemburgo	Macuco	tropical	plantation	local+catchment	omnivore	abundance, biomass, diversity

		substrates in Atlantic Forest streams? *Iheringia Série Zoologia*, 108, 1–10. https://doi.org/10.1590/1678-4766e2018014								
89	Kiffney & Richardson, 2010	Kiffney, P. M., & Richardson, J. S. (2010). Organic matter inputs into headwater streams of southwestern British Columbia as a function of riparian reserves and time since harvesting. *Forest Ecology and Management*, 260(11), 1931–1942. https://doi.org/10.1016/j.foreco.2010.08.016	Control	Clear-cut		temperate	harvest	local+catchment	detritus	biomass
90	Kominoski et al., 2011	Kominoski, J. S., Marczak, L. B., & Richardson, J. S. (2011). Riparian forest composition affects stream litter decomposition despite similar microbial and invertebrate communities. *Ecology*, 92(1), 151–159. https://doi.org/10.1890/10-0028.1	Deciduous forest	Conifer plantation	Mixed	temperate	harvest	local	detritus, omnivore, microbes	decomposition, biomass, abundance, diversity
91	Kreutzweiser et al., 2008	Kreutzweiser, D. P., Good, K. P., Capell, S. S., & Holmes, S. B. (2008). Leaf-litter decomposition and macroinvertebrate communities in boreal forest streams linked to upland logging disturbance. *Journal of the North American Benthological Society*, 27(1), 1–15. https://doi.org/10.1899/07-034R.1	Forested	Logged		boreal	harvest	local+catchment	detritus, omnivore, shredder	decomposition, abundance, diversity
92	Laćan et al., 2010	Laćan, I., Resh, V. H., & McBRIDE, J. R. (2010). Similar breakdown rates and benthic macroinvertebrate assemblages on native and *Eucalyptus globulus* leaf litter in Californian streams. *Freshwater Biology*, 55(4), 739–752. https://doi.org/10.1111/j.1365-2427.2009.02312.x	Native forest	Eucalyptus plantation		temperate	plantation	local	detritus, omnivore	biomass, abundance, diversity
93	Lamberti & Berg, 1995	Lamberti, G. A., & Berg, M. B. (1995). Invertebrates and other benthic features as indicators of environmental change in Juday Creek, Indiana. *Natural Areas Journal*, *15*(3), 249–258.	Woodland	Agriculture	Urban	temperate	conversion	local+catchment	detritus	biomass, abundance
94	Larrañaga et al., 2023	Larrañaga, A., Perkins, D. M., Basaguren, A., Larrañaga, S., Pozo, J., & Montoya, J. M. (2023). Land use drives detritivore size structure and decomposition through shifts in resource quality and quantity. *Science of the Total Environment*, *892*, 164552. https://doi.org/10.1016/j.scitotenv.2023.164552	Native Forest	Eucalyptus plantation		temperate	plantation	local+catchment	omnivore, shredder, detritus	abundance, biomass, diversity, decomposition
98	Lecerf et al., 2012	Lecerf, A., Baudoin, J.-M., Besson, A. A., Lamothe, S., & Lagrue, C. (2012). Is smaller necessarily better? Effects of small-scale forest harvesting on stream ecosystems. *Annales de Limnologie*, *48*(4), 401–409. https://doi.org/10.1051/limn/2012028	Mature forest	Harvested		temperate	harvest	local+catchment	omnivore, detritus	abundance, decomposition
99	Lecerf & Richardson, 2010	Lecerf, A., & Richardson, J. S. (2010). Litter decomposition can detect effects of high and moderate levels of forest disturbance on stream condition. *Forest Ecology and Management*, *259*(12), 2433–2443. https://doi.org/10.1016/j.foreco.2010.03.022	Forest	No reserve	Intermediate sites	temperate	harvest	local+catchment	detritus, shredder, microbes	decomposition, abundance, diversity, biomass
100	Lecerf & Chauvet, 2008	Lecerf, A., & Chauvet, E. (2008). Diversity and functions of leaf-decaying fungi in human-altered streams. *Freshwater Biology*, 53(8), 1658–1672. https://doi.org/10.1111/j.1365-2427.2008.01986.x	Control	Impact	Eutrophication and Mine pollution sites	temperate	harvest	catchment	detritus, microbes	decomposition, biomass, diversity

102	Link et al., 2022	Link, M., Schreiner, V. C., Graf, N., Szöcs, E., Bundschuh, M., Battes, K. P., Cîmpean, M., Sures, B., Grabner, D., Buse, J., & Schäfer, R. B. (2022). Pesticide effects on macroinvertebrates and leaf litter decomposition in areas with traditional agriculture. *Science of the Total Environment*, 828, 154549. https://doi.org/10.1016/j.scitotenv.2022.154549	With forest upstream	Without forest upstream		temperate	conversion	local+catchment	omnivore, shredder, detritus	diversity, abundance, decomposition
103	Lopes et al., 2015	Lopes, M. P., Martins, R. T., Silveira, L. S., & Alves, R. G. (2015). The leaf breakdown of *Picramnia sellowii* (Picramniales: Picramniaceae) as index of anthropic disturbances in tropical streams. *Brazilian Journal of Biology*, *75*, 846–853. https://doi.org/10.1590/1519-6984.00414	Forest	Impaired		tropical	harvest	local	detritus, microbes, shredder	decomposition, biomass, abundance
104	Lorion & Kennedy, 2009	Lorion, C. M., & Kennedy, B. P. (2009). Riparian forest buffers mitigate the effects of deforestation on fish assemblages in tropical headwater streams. *Ecological Applications*, *19*(2), 468–479. https://doi.org/10.1890/08-0050.1	Forest	Pasture	Intermediate sites	tropical	harvest	local+catchment	omnivore	abundance, biomass, diversity
105	Lubanga et al., 2021	Lubanga, H. L., Manyala, J. O., Sitati, A., Yegon, M. J., & Masese, F. O. (2021). Spatial variability in water quality and macroinvertebrate assemblages across a disturbance gradient in the Mara River Basin, Kenya. *Ecohydrology & Hydrobiology*, *21*(21), 718–730. https://doi.org/10.1016/j.ecohyd.2021.03.001	Undisturbed	Disturbed	Moderately Disturbed	tropical	conversion	local+catchment	omnivore, shredder	diversity, abundance
106	Mancuso et al., 2023	Mancuso, J., Tank, J. L., Mahl, U. H., Vincent, A., & Tiegs, S. D. (2023). Monthly variation in organic-matter decomposition in agricultural stream and riparian ecosystems. *Aquatic Sciences*, *85*(3), 83. https://doi.org/10.1007/s00027-023-00975-7	Forested	Agriculture	Intermediate sites	temperate	conversion	catchment	detritus	decomposition
110	Maridet et al., 1998	Maridet, L., Wasson, J.-G., Philippe, M., Amoros, C., & Naiman, R. (1998). Trophic structure of three streams with contrasting riparian vegetation and geomorphology. *Archiv für Hydrobiologie*, 61–85. https://doi.org/10.1127/archiv-hydrobiol/144/1998/61	Vianon	Ozange, Triouzone		temperate	harvest	catchment, local+catchment	omnivore, shredder	diversity, abundance, biomass
111	Márquez et al., 2017	Márquez, J. A., Principe, R. E., Cibils Martina, L., & Albariño, R. J. (2017). Pine needle litter acts as habitat but not as food source for stream invertebrates. *International Review of Hydrobiology*, 102(1–2), 29–37. https://doi.org/10.1002/iroh.201601856	Grassland	Afforested		subtropical	plantation	local+catchment	detritus, omnivore, shredder	decomposition, diversity, abundance
112	Martínez et al., 2013	Martínez, A., Larrañaga, A., Pérez, J., Descals, E., Basaguren, A., & Pozo, J. (2013). Effects of pine plantations on structural and functional attributes of forested streams. *Forest Ecology and Management*, 310, 147–155. https://doi.org/10.1016/j.foreco.2013.08.024	Forest	Plantation		temperate	plantation	catchment	detritus, omnivore, shredder, microbes	decomposition, biomass, diversity, abundance
113	Masese et al., 2014	Masese, F. O., Kitaka, N., Kipkemboi, J., Gettel, G. M., Irvine, K., & McClain, M. E. (2014). Litter processing and shredder distribution as indicators of riparian and catchment influences on ecological health of tropical	Forest	Agriculture	Mixed	tropical	conversion	local+catchment	detritus, omnivore, shredder	biomass, diversity, abundance, decomposition

		streams. *Ecological Indicators*, 46, 23–37. https://doi.org/10.1016/j.ecolind.2014.05.032							
114	Mckie & Malmqvist, 2009	Mckie, B. G., & Malmqvist, B. (2009). Assessing ecosystem functioning in streams affected by forest management: Increased leaf decomposition occurs without changes to the composition of benthic assemblages. *Freshwater Biology*, *54*(10), 2086–2100. https://doi.org/10.1111/j.1365-2427.2008.02150.x	Forest	Clear-cut	Mixed	boreal	harvest	local+catchment	detritus, omnivore, shredder	decomposition, abundance, diversity, biomass
115	McNeish et al., 2017	McNeish, R. E., Benbow, M. E., & McEwan, R. W. (2017). Removal of the invasive shrub, *Lonicera maackii* (Amur Honeysuckle), from a headwater stream riparian zone shifts taxonomic and functional composition of the aquatic biota. *Invasive Plant Science and Management*, 10(3), 232–246. https://doi.org/10.1017/inp.2017.22	Invasive species removed sites	Invaded sites		temperate	restoration	local	omnivore, shredder	abundance, diversity
116	McNeish et al., 2015	McNeish, R. E., Moore, E. M., Benbow, M. E., & McEwan, R. W. (2015). Removal of the Invasive Shrub, *Lonicera maackii*, from Riparian Forests Influences Headwater Stream Biota and Ecosystem Function. *River Research and Applications*, 31(9), 1131–1139. https://doi.org/10.1002/rra.2808	Invasive species removed sites	Invaded sites		temperate	restoration	local	detritus	biomass
117	Mctammany et al., 2008	Mctammany, M. E., Benfield, E. F., & Webster, J. R. (2008). Effects of agriculture on wood breakdown and microbial biofilm respiration in southern Appalachian streams. *Freshwater Biology*, 53(4), 842–854. https://doi.org/10.1111/j.1365-2427.2007.01936.x	Forest; Recovered	AG-L, AG-H	Other sites	temperate	conversion, restoration	local, local+catchment, catchment	detritus	decomposition
118	Menéndez et al., 2013	Menéndez, M., Descals, E., Riera, T., & Moya, O. (2013). Do non-native *Platanus hybrida* riparian plantations affect leaf litter decomposition in streams? *Hydrobiologia*, *716*(1), 5–20. https://doi.org/10.1007/s10750-013-1539-0	Forest	Plantation		temperate	plantation	local	detritus, omnivore, shredder, microbes	decomposition, abundance, diversity
119	Menninger & Palmer, 2007	Menninger, H. L., & Palmer, M. A. (2007). Herbs and grasses as an allochthonous resource in open-canopy headwater streams. *Freshwater Biology*, 52(9), 1689–1699. https://doi.org/10.1111/j.1365-2427.2007.01797.x	RB	FCQ	CC	temperate	conversion	catchment	detritus, omnivore, shredder	biomass, abundance
120	Mesa et al., 2013	Mesa, L. M., Reynaga, M. C., Correa, M. del V., & Sirombra, M. G. (2013). Effects of anthropogenic impacts on benthic macroinvertebrates assemblages in subtropical mountain streams. *Iheringia. Série Zoologia*, 103, 342–349. https://doi.org/10.1590/S0073-47212013000400002	Unimpacted	Impacted		subtropical	conversion	local	detritus, shredder	biomass, abundance, diversity
121	Mlambo et al., 2019	Mlambo, M. C., Paavola, R., Fritze, H., Louhi, P., & Muotka, T. (2019). Leaf litter decomposition and decomposer communities in streams affected by intensive forest biomass removal. *Ecological Indicators*, *101*, 364–372. https://doi.org/10.1016/j.ecolind.2019.01.035	Natural	Logged with stumps removed	Logged without stumps removed	boreal	harvest	catchment	detritus, shredder, microbes	decomposition, abundance, diversity, biomass
122	Molinero et al., 1996	Molinero, J., Pozo, J., & Gonzalez, E. (1996). Litter breakdown in streams of the Agüera catchment: Influence of dissolved nutrients and land use. Freshwater Biology, 36(3), 745–756. https://doi.org/10.1046/j.1365-2427.1996.00125.x	Forest	Plantation		temperate	plantation	local+catchment	detritus	decomposition, biomass

123	Monroy et al., 2017	Monroy, S., Martínez, A., López-Rojo, N., Pérez-Calpe, A. V., Basaguren, A., & Pozo, J. (2017). Structural and functional recovery of macroinvertebrate communities and leaf litter decomposition after a marked drought: Does vegetation type matter? *Science of the Total Environment*, *599–600*, 1241–1250. https://doi.org/10.1016/j.scitotenv.2017.05.093	Deciduous forest	Eucalyptus and Pine plantation		temperate	plantation	local+catchment	omnivore, shredder, detritus	abundance, diversity, biomass, decomposition
124	Morabowen et al., 2019	Morabowen, A., Crespo-Pérez, V., & Ríos-Touma, B. (2019). Effects of agricultural landscapes and land uses in highly biodiverse tropical streams of the Ecuadorian Choco. *Inland Waters*, *9*(3), 289–300. https://doi.org/10.1080/20442041.2018.1527597	Forest	Organic farming; Monoculture		tropical	conversion	catchment, local+catchment	omnivore, detritus	diversity, biomass
125	Murphy & Giller, 2001	Murphy, J. F., & Giller, P. S. (2001). Hazel leaf breakdown in two low-order streams differing in the functional efficiency of their detritivore assemblages. *Archiv für Hydrobiologie*, 249–267. https://doi.org/10.1127/archiv-hydrobiol/150/2001/249	Forest	Conifer plantation	Transplantation experiment results	temperate	plantation	local+catchment	detritus, omnivore, shredder	decomposition, abundance
126	Musetta-Lambert et al., 2017	Musetta-Lambert, J., Muto, E., Kreutzweiser, D., & Sibley, P. (2017). Wildfire in boreal forest catchments influences leaf litter subsidies and consumer communities in streams: Implications for riparian management strategies. *Forest Ecology and Management*, *391*, 29–41. https://doi.org/10.1016/j.foreco.2017.01.028	Forest	Harvested	Fire	boreal	harvest	local+catchment	detritus, omnivore, shredder	biomass, decomposition, abundance, diversity
127	Myers et al., 2007	Myers, L., Mihuc, T., & Woodcock, T. (2007). The impacts of forest management on invertebrate communities associated with submerged leaves in forested Adirondack streams. *Journal of Freshwater Ecology*, *22*(2), 325–331. https://doi.org/10.1080/02705060.2007.9665054	Preserve	Logged		temperate	harvest	catchment	omnivore	abundance
128	Oelbermann & Gordon, 2000	Oelbermann, M., & Gordon, A. M. (2000). Quantity and quality of autumnal litterfall into a rehabilitated agricultural stream. *Journal of Environmental Quality*, *29*(2), 603–611. https://doi.org/10.2134/jeq2000.00472425002900020031x	Wide buffer	Narrow buffer	Other sites	temperate	conversion	local	detritus	biomass
129	Oester et al., 2023	Oester, R., dos Reis Oliveira, P. C., Moretti, M. S., Altermatt, F., & Bruder, A. (2023). Leaf-associated macroinvertebrate assemblage and leaf litter breakdown in headwater streams depend on local riparian vegetation. *Hydrobiologia*, *850*(15), 3359–3374. https://doi.org/10.1007/s10750-022-05049-7	Forested	Non-forested		temperate	harvest	local	detritus, omnivore, shredder	decomposition, abundance, diversity, biomass
130	Ono et al., 2020	Ono, E. R., Manoel, P. S., Melo, A. L. U., & Uieda, V. S. (2020). Effects of riparian vegetation removal on the functional feeding group structure of benthic macroinvertebrate assemblages. *Community Ecology*, *21*(2), 145–157. https://doi.org/10.1007/s42974-020-00014-7	Forest	Pasture		tropical	conversion	local	shredder, omnivore, detritus	abundance, diversity
131	Paul et al., 2006	Paul, M. J., Meyer, J. L., & Couch, C. A. (2006). Leaf breakdown in streams differing in catchment land use. *Freshwater Biology*, *51*(9), 1684–1695. https://doi.org/10.1111/j.1365-2427.2006.01612.x	Forest	Agriculture	Suburban and urban sites	temperate	conversion	catchment	detritus, microbes, shredder	decomposition, biomass, abundance

132	Principe et al., 2015	Principe, R. E., Márquez, J. A., Martina, L. C., Jobbágy, E. G., & Albariño, R. J. (2015). Pine afforestation changes more strongly community structure than ecosystem functioning in grassland mountain streams. *Ecological Indicators*, *57*, 366–375. https://doi.org/10.1016/j.ecolind.2015.04.033	Natural grassland	Pine plantation		subtropical	plantation	local+catchment	detritus, omnivore, shredder	decomposition, diversity, abundance
133	Reid et al., 2013	Reid, D. J., Lake, P. S., & Quinn, G. P. (2013). Influences of agricultural landuse and seasonal changes in abiotic conditions on invertebrate colonisation of riparian leaf detritus in intermittent streams. *Aquatic Sciences*, *75*(2), 285–297. https://doi.org/10.1007/s00027-012-0273-4	Reserve	Farmland		subtropical	conversion	local	omnivore	diversity, abundance
134	Rezende et al., 2021	Rezende, R. de S., Cararo, E. R., Bernardi, J. P., Chimello, V., Lima-Rezende, C. A., Albeny-Simões, D., Dal Magro, J., & Gonçalves, J. F. (2021). Land cover affects the breakdown of *Pinus elliottii* needles litter by microorganisms in soil and stream systems of subtropical riparian zones. *Limnologica*, *90*, 125905. https://doi.org/10.1016/j.limno.2021.125905	Forest, grassland with riparian vegetation	Silvicultre, grassland without riparian vegetation		subtropical	harvest, conversion	local+catchment, local	detritus	biomass
135	Riipinen et al., 2009	Riipinen, M. P., Fleituch, T., Hladyz, S., Woodward, G., Giller, P., & Dobson, M. (2009). Invertebrate community structure and ecosystem functioning in European conifer plantation streams. *Freshwater Biology*, 55, 346–359. https://doi.org/10.1111/j.1365-2427.2009.02278.x	Broadleaf forest	Conifer plantation		temperate	plantation	local+catchment	detritus, shredder	decomposition, abundance, diversity, biomass
137	Roberts & Bilby, 2009	Roberts, M. L., & Bilby, R. E. (2009). Urbanization alters litterfall rates and nutrient inputs to small Puget Lowland streams. *Journal of the North American Benthological Society*, *28*(4), 941–954. https://doi.org/10.1899/07-160.1	Reference	Disturbed	Intermediate sites	temperate	conversion	local	detritus	biomass
138	Rossi et al., 2019	Rossi, F., Mallet, C., Portelli, C., Donnadieu, F., Bonnemoy, F., & Artigas, J. (2019). Stimulation or inhibition: Leaf microbial decomposition in streams subjected to complex chemical contamination. *Science of the Total Environment*, *648*, 1371–1383. https://doi.org/10.1016/j.scitotenv.2018.08.197	Forest	Agriculure	Urban	temperate	conversion	catchment	detritus, microbes	decomposition, biomass, diversity
140	Rubio-Ríos et al., 2023	Rubio-Ríos, J., Salinas-Bonillo, M. J., Pérez, J., Fenoy, E., Boyero, L., & Casas, J. J. (2023). Alder stands promote N-cycling but not leaf litter mass loss in Mediterranean streams flowing through pine plantations. *Forest Ecology and Management*, *542*, 121072. https://doi.org/10.1016/j.foreco.2023.121072	With Alder	Without Alder	Mixtures	temperate	restoration	local	detritus	decomposition
141	Ryan & Kelly-Quinn, 2016	Ryan, D. K., & Kelly-Quinn, M. (2016). Riparian vegetation management for water temperature regulation: Implications for the production of macroinvertebrate prey of salmonids. *Fisheries Management and Ecology*, *23*(6), 519–530. https://doi.org/10.1111/fme.12193	Shaded riparian cover	Unshaded riparian cover		temperate	conversion	local	omnivore	abundance
142	Ryder et al., 2011	Ryder, L., de Eyto, E., Gormally, M., Skeffington, M. S., Dillane, M., & Poole, R. (2011). Riparian zone creation in established coniferous forests in Irish upland peat catchments: Physical, chemical and biological	Pre-felling	Post-felling	Other sites	temperate	harvest	local	omnivore	diversity, abundance

		implications. *Biology and Environment: Proceedings of the Royal Irish Academy*, *111B*(1), 41–60.							
143	Sakai et al., 2013	Sakai, M., Natuhara, Y., Fukushima, K., Imanishi, A., Imai, K., & Kato, M. (2013). Ecological functions of persistent Japanese cedar litter in structuring stream macroinvertebrate assemblages. *Journal of Forest Research*, *18*(2), 190–199. https://doi.org/10.1007/s10310-012-0339-0	Forest	Plantation		temperate	plantation	catchment	detritus, omnivore, shredder	biomass, abundance, diversity
145	Sarriquet et al., 2006	Sarriquet, P. E., Delettre, Y. R., & Marmonier, P. (2006). Effects of catchment disturbance on stream invertebrates: Comparison of different habitats (vegetation, benthic and interstitial) using bio-ecological groups. *Annales de Limnologie*, *42*(4), 205–219. https://doi.org/10.1051/limn/2006022	Forest	Agriculture	Intermediate and otherwise heavily disturbed sites	temperate	conversion	local	omnivore	diversity, abundance
146	Shah et al., 2021	Shah, N. W., Nisbet, T. R., & Broadmeadow, S. B. (2021). The impacts of conifer afforestation and climate on water quality and freshwater ecology in a sensitive peaty catchment: A 25 year study in the upper River Halladale in North Scotland. *Forest Ecology and Management*, *502*, 119616. https://doi.org/10.1016/j.foreco.2021.119616	BB	UH	Other sites	temperate	conversion	local+catchment	omnivore	diversity, abundance
147	Silva-Araújo et al., 2020	Silva-Araújo, M., Silva-Junior, E. F., Neres-Lima, V., Feijó-Lima, R., Tromboni, F., Lourenço-Amorim, C., Thomas, S. A., Moulton, T. P., & Zandonà, E. (2020). Effects of riparian deforestation on benthic invertebrate community and leaf processing in Atlantic forest streams. *Perspectives in Ecology and Conservation*, *18*(4), 277–282. https://doi.org/10.1016/j.pecon.2020.09.004	Least deforested	Most deforested	Intermediate sites	tropical	harvest	local	detritus, omnivore, shredder	decomposition, abundance, diversity
150	Suga & Tanaka, 2013	Suga, C. M., & Tanaka, M. O. (2013). Influence of a forest remnant on macroinvertebrate communities in a degraded tropical stream. *Hydrobiologia*, *703*(1), 203–213. https://doi.org/10.1007/s10750-012-1360-1	Downstream forest	Upstream sugarcane		tropical	conversion	local	omnivore, shredder	abundance, diversity
151	Tanaka et al., 2015	Tanaka, M. O., Fernandes, J. de F., Suga, C. M., Hanai, F. Y., & Souza, A. L. T. de. (2015). Abrupt change of a stream ecosystem function along a sugarcane-forest transition: Integrating riparian and in-stream characteristics. *Agriculture, Ecosystems & Environment*, 207, 171–177. https://doi.org/10.1016/j.agee.2015.04.014	Downstream forest	Upstream sugarcane		tropical	conversion	local	detritus	decomposition
152	Tolkkinen et al., 2015	Tolkkinen, M., Mykrä, H., Annala, M., Markkola, A. M., Vuori, K. M., & Muotka, T. (2015). Multi-stressor impacts on fungal diversity and ecosystem functions in streams: Natural vs. anthropogenic stress. *Ecology*, *96*(3), 672–683. https://doi.org/10.1890/14-0743.1	Pristine	disturbed		boreal	harvest	local+catchment	detritus, microbes	decomposition, biomass, diversity
153	Tonello et al., 2021	Tonello, G., Decian, V. S., Restello, R. M., & Hepp, L. U. (2021). The conversion of natural riparian forests into agricultural land affects ecological processes in Atlantic forest streams. *Limnologica*, *91*, 125927. https://doi.org/10.1016/j.limno.2021.125927	Least agriculture	Most agriculture	Intermediate sites	subtropical	conversion	local	detritus, omnivore, shredder	decomposition, abundance

154	Torres & Ramírez, 2014	Torres, P. J., & Ramírez, A. (2014). Land use effects on leaf litter breakdown in low-order streams draining a rapidly developing tropical watershed in Puerto Rico. *Revista de Biología Tropical*, *62*(S2), Article S2. https://doi.org/10.15517/rbt.v62i0.15783	Forest	Agriculture	Urban	tropical	conversion	local+catchment	detritus	decomposition
155	Townsend et al., 2004	Townsend, C. R., Downes, B. J., Peacock, K., & Arbuckle, C. J. (2004). Scale and the detection of land-use effects on morphology, vegetation and macroinvertebrate communities of grassland streams. *Freshwater Biology*, *49*(4), 448–462. https://doi.org/10.1111/j.1365-2427.2004.01192.x	Tussock	Pasture		temperate	conversion	local+catchment	omnivore	diversity, abundance
156	Truchy et al., 2022	Truchy, A., Sponseller, R. A., Ecke, F., Angeler, D. G., Kahlert, M., Bundschuh, M., Johnson, R. K., & McKie, B. G. (2022). Responses of multiple structural and functional indicators along three contrasting disturbance gradients. *Ecological Indicators*, *135*, 108514. https://doi.org/10.1016/j.ecolind.2021.108514	Most forested	Least forested	Intermediate sites	boreal	harvest	catchment	omnivore, shredder, detritus, microbes	abundance, diversity, decomposition, biomass
157	Tupinambás et al., 2007	Tupinambás, T. H., Callisto, M., & Santos, G. B. (2007). Benthic macroinvertebrate assemblages structure in two headwater streams, south-eastern Brazil. *Revista Brasileira de Zoologia*, 24(4), 887–897. https://doi.org/10.1590/S0101-81752007000400005	1B, 2B	1A, 2A ,3A, 3B		tropical	conversion	local	omnivore	abundance, diversity
158	Turunen et al., 2017	Turunen, J., Aroviita, J., Marttila, H., Louhi, P., Laamanen, T., Tolkkinen, M., Luhta, P.-L., Kløve, B., & Muotka, T. (2017). Differential responses by stream and riparian biodiversity to in-stream restoration of forestry-impacted streams. *Journal of Applied Ecology*, *54*(5), 1505–1514. https://doi.org/10.1111/1365-2664.12897	Pristine	Impaired	Restored sites	boreal	harvest	local+catchment	omnivore, detritus, microbes	abundance, diversity, decomposition, biomass
159	Valente-Neto et al., 2015	Valente-Neto, F., Koroiva, R., Fonseca-Gessner, A. A., & Roque, F. de O. (2015). The effect of riparian deforestation on macroinvertebrates associated with submerged woody debris. *Aquatic Ecology*, 49(1), 115–125. https://doi.org/10.1007/s10452-015-9510-y	Most complete riparian zone	Least complete riparian zone	Intermediate sites	tropical	conversion	local	omnivore	abundance, diversity
161	Walsh et al., 2002	Walsh, C. J., Gooderham, J. P. R., Grace, M. R., Sdraulig, S., Rosyidi, M. I., & Lelono, A. (2002). The relative influence of diffuse- and point-source disturbances on a small upland stream in East Java, Indonesia: A preliminary investigation. *Hydrobiologia*, *487*(1), 183–192. https://doi.org/10.1023/A:1022990822198	UF	UV	Intermediate sites	tropical	plantation	local	omnivore	diversity
162	Whiles & Wallace, 1997	Whiles, M. R., & Wallace, J. B. (1997). Leaf litter decomposition and macroinvertebrate communities in headwater streams draining pine and hardwood catchments. *Hydrobiologia*, *353*(1), 107–119. https://doi.org/10.1023/A:1003054827248	Hardwood	Pine plantation		temperate	plantation	catchment	shredder, omnivore, detritus	abundance, biomass, decomposition
163	Yeung et al., 2017	Yeung, A. C. Y., Lecerf, A., & Richardson, J. S. (2017). Assessing the long-term ecological effects of riparian management practices on headwater streams in a coastal temperate rainforest. *Forest Ecology and Management*, *384*, 100–109. https://doi.org/10.1016/j.foreco.2016.10.044	Forest	Clear-cut		temperate	harvest	local+catchment	detritus, shredder	decomposition, abundance, diversity

164	Youngquist et al., 2020	Youngquist, M. B., Wiley, C., Eggert, S. L., D'Amato, A. W., Palik, B. J., & Slesak, R. A. (2020). Foundation species loss affects leaf breakdown and aquatic invertebrate resource use in black ash wetlands. *Wetlands*, *40*(4), 839–852. https://doi.org/10.1007/s13157-019-01221-3	Forest	Clear-cut	temperate	harvest	local	detritus	decomposition
166	Zhang et al., 2009	Zhang, Y., Richardson, J. S., & Pinto, X. (2009). Catchment-scale effects of forestry practices on benthic invertebrate communities in Pacific coastal streams. *Journal of Applied Ecology*, *46*(6), 1292–1303. https://doi.org/10.1111/j.1365-2664.2009.01718.x	Forest	Logged	temperate	harvest	catchment	omnivore, shredder	biomass, abundance

Full reference list

Abelho, M., & Graça, M. A. S. (1996). Effects of eucalyptus afforestation on leaf litter dynamics and macroinvertebrate community structure of streams in Central Portugal. *Hydrobiologia*, *324*(3), 195–204. https://doi.org/10.1007/BF00016391

Aguiar, A. C. F., Neres-Lima, V., & Moulton, T. P. (2018). Relationships of shredders, leaf processing and organic matter along a canopy cover gradient in tropical streams. *Journal of Limnology*, *77*(1). https://doi.org/10.4081/jlimnol.2017.1684

Albertson, L. K., Ouellet, V., & Daniels, M. D. (2018). Impacts of stream riparian buffer land use on water temperature and food availability for fish. *Journal of Freshwater Ecology*, *33*(1), 195–210. https://doi.org/10.1080/02705060.2017.1422558

Astudillo, M. R., Novelo-Gutiérrez, R., Vázquez, G., García-Franco, J. G., & Ramírez, A. (2016). Relationships between land cover, riparian vegetation, stream characteristics, and aquatic insects in cloud forest streams, Mexico. *Hydrobiologia*, *768*(1), 167–181. https://doi.org/10.1007/s10750-015-2545-1

Bacca, J. C., Rampanelli Cararo, E., Alves Lima-Rezende, C., Tavares Martins, R., Macedo-Reis, L. E., Dal Magro, J., & De Souza Rezende, R. (2023). Land-use effects on aquatic macroinvertebrate diversity in subtropical highland grasslands streams. *Limnetica*, *42*(2), 1. https://doi.org/10.23818/limn.42.16

Bärlocher, F., Helson, J. E., & Williams, D. D. (2010). Aquatic hyphomycete communities across a land-use gradient of Panamanian streams. *Fundamental and Applied Limnology*, *177*(3), 209–221. https://doi.org/10.1127/1863-9135/2010/0177-0209

Barrios, M., Burwood, M., Kröger, A., Calvo, C., Ríos-Touma, B., & Teixeira-de-Mello, F. (2022). Riparian cover buffers the effects of abiotic and biotic predictors of leaf decomposition in subtropical streams. *Aquatic Sciences*, *84*(4), 55. https://doi.org/10.1007/s00027-022-00886-z

Basaguren, A., & Pozo, J. (1994). Leaf litter processing of alder and eucalyptus in the Agüera stream system (Northern Spain). II: Macroinvertebrates associated. *Archiv für Hydrobiologie*, *132*(1), 57–68.

Benfield, E. F., Webster, J. R., Tank, J. L., & Hutchens, J. J. (2001). Long-term patterns in leaf breakdown in streams in response to watershed logging. *International Review of Hydrobiology*, *86*(4–5), 467–474. https://doi.org/10.1002/1522-2632(200107)86:4/5<467::AID-IROH467>3.0.CO;2-1

Bertaso, T. R. N., Spies, M. R., Kotzian, C. B., & Flores, M. L. T. (2015). Effects of forest conversion on the assemblages' structure of aquatic insects in subtropical regions. *Revista Brasileira de Entomologia*, *59*(1), 43–49. https://doi.org/10.1016/j.rbe.2015.02.005

Burrows, R. M., Magierowski, R. H., Fellman, J. B., & Barmuta, L. A. (2012). Woody debris input and function in old-growth and clear-felled headwater streams. *Forest Ecology and Management*, *286*, 73–80. https://doi.org/10.1016/j.foreco.2012.08.038

Burrows, R. M., Magierowski, R. H., Fellman, J. B., Clapcott, J. E., Munks, S. A., Roberts, S., Davies, P. E., & Barmuta, L. A. (2014). Variation in stream organic matter processing among years and benthic habitats in response to forest clearfelling. *Forest Ecology and Management*, *327*, 136–147. https://doi.org/10.1016/j.foreco.2014.04.041

Burwood, M., Clemente, J., Meerhoff, M., Iglesias, C., Goyenola, G., Fosalba, C., Pacheco, J. P., & Teixeira De Mello, F. (2021). Macroinvertebrate communities and macrophyte decomposition could be affected by land use intensification in subtropical lowland streams. *Limnetica*, *40*(2), 343–357. https://doi.org/10.23818/limn.40.23

Bylak, A., Kukuła, K., Ortyl, B., Hałoń, E., Demczyk, A., Janora-Hołyszko, K., Maternia, J., Szczurowski, Ł., & Ziobro, J. (2022). Small stream catchments in a developing city context: The importance of land cover changes on the ecological status of streams and the possibilities for providing ecosystem services. *Science of the Total Environment*, *815*, 151974. https://doi.org/10.1016/j.scitotenv.2021.151974

Carroll, D., & Jackson, G. R. (2009). Observed relationships between urbanization and riparian cover, shredder abundance, and stream leaf litter standing crops. *Fundamental and Applied Limnology*, 213–225. https://doi.org/10.1127/1863-9135/2008/0173-0213

Casas, J. J., Larrañaga, A., Menéndez, M., Pozo, J., Basaguren, A., Martínez, A., Pérez, J., González, J. M., Mollá, S., Casado, C., Descals, E., Roblas, N., López-González, J. A., & Valenzuela, J. L. (2013). Leaf litter decomposition of native and introduced tree species of contrasting quality in headwater streams: How does the regional setting matter? *Science of the Total Environment*, *458–460*, 197–208. https://doi.org/10.1016/j.scitotenv.2013.04.004

Casotti, C. G., Kiffer, W. P., Costa, L. C., Rangel, J. V., Casagrande, L. C., & Moretti, M. S. (2015). Assessing the importance of riparian zones conservation for leaf decomposition in streams. *Natureza & Conservação*, *13*(2), 178–182. https://doi.org/10.1016/j.ncon.2015.11.011

Chellaiah, D., & Yule, C. M. (2018). Litter decomposition is driven by microbes and is more influenced by litter quality than environmental conditions in oil palm streams with different riparian types. *Aquatic Sciences*, *80*(4), 43. https://doi.org/10.1007/s00027-018-0595-y

Chellaiah, D., & Yule, C. M. (2019). Litter quality influences bacterial communities more strongly than changes in riparian buffer quality in oil palm streams. *Aquatic Microbial Ecology*, *83*(2), 167–179. https://doi.org/10.3354/ame01909

Collier, K. J., & Halliday, J. N. (2000). Macroinvertebrate-wood associations during decay of plantation pine in New Zealand pumice-bed streams: Stable habitat or trophic subsidy? *Journal of the North American Benthological Society*, *19*(1), 94–111. https://doi.org/10.2307/1468284

Collier, K. J., Smith, B. J., & Halliday, N. J. (2004). Colonization and use of pine wood versus native wood in New Zealand plantation forest streams: Implications for riparian management. *Aquatic Conservation: Marine and Freshwater Ecosystems*, *14*(2), 179–199. https://doi.org/10.1002/aqc.599

Cordero–Rivera, A., Álvarez, A. M., & Álvarez, M. (2017). Eucalypt plantations reduce the diversity of macroinvertebrates in small forested streams. *Animal Biodiversity and Conservation*, *40*(1), Article 1. https://doi.org/10.32800/abc.2017.40.0087

Cornejo, A., Pérez, J., López-Rojo, N., Tonin, A. M., Rovira, D., Checa, B., Jaramillo, N., Correa, K., Villarreal, A., Villarreal, V., García, G., Pérez, E., Ríos González, T. A., Aguirre, Y., Correa-Araneda, F., & Boyero, L. (2020). Agriculture impairs stream ecosystem functioning in a tropical catchment. *Science of the Total Environment*, *745*, 140950. https://doi.org/10.1016/j.scitotenv.2020.140950

da Costa, I. D., Petry, A. C., & Mazzoni, R. (2020). Fish assemblages respond to forest cover in small Amazonian basins. *Limnologica*, *81*, 125757. https://doi.org/10.1016/j.limno.2020.125757

Danger, A. R., & Robson, B. J. (2004). The effects of land use on leaf-litter processing by macroinvertebrates in an Australian temperate coastal stream. *Aquatic Sciences*, *66*(3), 296–304. https://doi.org/10.1007/s00027-004-0718-5

Daoust, K., Kreutzweiser, D. P., Guo, J., Creed, I. F., & Sibley, P. K. (2019). Climate-influenced catchment hydrology overrides forest management effects on stream benthic macroinvertebrates in a northern hardwood forest. *Forest Ecology and Management*, *452*, 117540. https://doi.org/10.1016/j.foreco.2019.117540

de Mello Cionek, V., Fogaça, F. N. O., Moulton, T. P., Pazianoto, L. H. R., Landgraf, G. O., & Benedito, E. (2021). Influence of leaf miners and environmental quality on litter breakdown in tropical headwater streams. *Hydrobiologia*, *848*(6), 1311–1331. https://doi.org/10.1007/s10750-021-04529-6

de Nadaï-Monoury, E., Gilbert, F., & Lecerf, A. (2014). Forest canopy cover determines invertebrate diversity and ecosystem process rates in depositional zones of headwater streams. *Freshwater Biology*, *59*(7), 1532–1545. https://doi.org/10.1111/fwb.12364

Delong, M. D., & Brusven, M. A. (1994). Allochthonous input of organic matter from different riparian habitats of an agriculturally impacted stream. *Environmental Management*, *18*(1), 59–71. https://doi.org/10.1007/BF02393750

Dézerald, O., Talaga, S., Leroy, C., Carrias, J.-F., Corbara, B., Dejean, A., & Céréghino, R. (2014). Environmental determinants of macroinvertebrate diversity in small water bodies: Insights from tank-bromeliads. *Hydrobiologia*, *723*(1), 77–86. https://doi.org/10.1007/s10750-013-1464-2

Díez, J., Elosegi, A., Chauvet, E., & Pozo, J. (2002). Breakdown of wood in the Agüera stream. *Freshwater Biology*, *47*(11), 2205–2215. https://doi.org/10.1046/j.1365-2427.2002.00965.x

dos Santos, F. B., Ferreira, F. C., & Esteves, K. E. (2015). Assessing the importance of the riparian zone for stream fish communities in a sugarcane dominated landscape (Piracicaba River Basin, Southeast Brazil). *Environmental Biology of Fishes*, *98*(8), 1895–1912. https://doi.org/10.1007/s10641-015-0406-4

Dudgeon, D. (2006). The impacts of human disturbance on stream benthic invertebrates and their drift in North Sulawesi, Indonesia. *Freshwater Biology*, *51*(9), 1710–1729. https://doi.org/10.1111/j.1365-2427.2006.01596.x

Dudley, M. P., Solomon, K., Wenger, S., Jackson, C. R., Freeman, M., Elliott, K. J., Miniat, C. F., & Pringle, C. M. (2021). Do crayfish affect stream ecosystem response to riparian vegetation removal? *Freshwater Biology*, *66*(7), 1423–1435. https://doi.org/10.1111/fwb.13728

Ebling, L. A., Pastore, B. L., Biasi, C., Hepp, L. U., & Restello, R. M. (2024). Does environmental variability in Atlantic Forest streams affect aquatic hyphomycete and invertebrate assemblages associated with leaf litter? *Hydrobiologia*, *851*(7), 1761–1777. https://doi.org/10.1007/s10750-023-05415-z

Elosegi, A., Díez, J., & Pozo, J. (2007). Contribution of dead wood to the carbon flux in forested streams. *Earth Surface Processes and Landforms*, *32*(8), 1219–1228. https://doi.org/10.1002/esp.1549

Elosegi, A., Nicolás, A., & Richardson, J. S. (2018). Priming of leaf litter decomposition by algae seems of minor importance in natural streams during autumn. *PLOS ONE*, *13*(9), e0200180. https://doi.org/10.1371/journal.pone.0200180

Ely, D. T., & Wallace, J. B. (2010). Long-term functional group recovery of lotic macroinvertebrates from logging disturbance. *Canadian Journal of Fisheries and Aquatic Sciences*, *67*(7), 1126–1134. https://doi.org/10.1139/F10-045

Emilson, C. E., Kreutzweiser, D. P., Gunn, J. M., & Mykytczuk, N. C. S. (2016). Effects of land use on the structure and function of leaf-litter microbial communities in boreal streams. *Freshwater Biology*, *61*(7), 1049–1061. https://doi.org/10.1111/fwb.12765

Encalada, A. C., Calles, J., Ferreira, V., Canhoto, C. M., & Graça, M. A. S. (2010). Riparian land use and the relationship between the benthos and litter decomposition in tropical montane streams. *Freshwater Biology*, *55*(8), 1719–1733. https://doi.org/10.1111/j.1365-2427.2010.02406.x

Erdozain, M., Kidd, K. A., Emilson, E. J. S., Capell, S. S., Luu, T., Kreutzweiser, D. P., & Gray, M. A. (2021). Forest management impacts on stream integrity at varying intensities and spatial scales: Do biological effects accumulate spatially? *Science of the Total Environment*, *763*, 144043. https://doi.org/10.1016/j.scitotenv.2020.144043

Erdozain, M., Kidd, K., Kreutzweiser, D., & Sibley, P. (2018). Linking stream ecosystem integrity to catchment and reach conditions in an intensively managed forest landscape. *Ecosphere*, *9*(5), e02278. https://doi.org/10.1002/ecs2.2278

Espinoza-Toledo, A., Mendoza-Carranza, M., Castillo, M. M., Barba-Macías, E., & Capps, K. A. (2021). Taxonomic and functional responses of macroinvertebrates to riparian forest conversion in tropical streams. *Science of the Total Environment*, *757*, 143972. https://doi.org/10.1016/j.scitotenv.2020.143972

Evangelista, C., Boiche, A., Lecerf, A., & Cucherousset, J. (2014). Ecological opportunities and intraspecific competition alter trophic niche specialization in an opportunistic stream predator. *Journal of Animal Ecology*, *83*(5), 1025–1034. https://doi.org/10.1111/1365-2656.12208

Fargen, C., Emery, S. M., & Carreiro, M. M. (2015). Influence of *Lonicera maackii* invasion on leaf litter decomposition and macroinvertebrate communities in an urban stream. *Natural Areas Journal*, *35*(3), 392–403. https://doi.org/10.3375/043.035.0303

Fasching, C., Akotoye, C., Bižić, M., Fonvielle, J., Ionescu, D., Mathavarajah, S., Zoccarato, L., Walsh, D. A., Grossart, H.-P., & Xenopoulos, M. A. (2020). Linking stream microbial community functional genes to dissolved organic matter and inorganic nutrients. *Limnology and Oceanography*, *65*(S1), S71–S87. https://doi.org/10.1002/lno.11356

Ferreira, V., Boyero, L., Calvo, C., Correa, F., Figueroa, R., Gonçalves, J. F., Goyenola, G., Graça, M. A. S., Hepp, L. U., Kariuki, S., López-Rodríguez, A., Mazzeo, N., M'Erimba, C., Monroy, S., Peil, A., Pozo, J., Rezende, R., & Teixeira-de-Mello, F. (2019). A Global Assessment of the Effects of Eucalyptus Plantations on Stream Ecosystem Functioning. *Ecosystems*, *22*(3), 629–642. https://doi.org/10.1007/s10021-018-0292-7

Ferreira, V., Elosegi, A., Gulis, V., Pozo, J., & Graça, M. A. S. (2006). Eucalyptus plantations affect fungal communities associated with leaf-litter decomposition in Iberian streams. *Archiv für Hydrobiologie*, *166*, 467–490. https://doi.org/10.1127/0003-9136/2006/0166-0467

Ferreira, V., Faustino, H., Raposeiro, P. M., & Gonçalves, V. (2017). Replacement of native forests by conifer plantations affects fungal decomposer community structure but not litter decomposition in Atlantic island streams. *Forest Ecology and Management*, *389*, 323–330. https://doi.org/10.1016/j.foreco.2017.01.004

Ferreira, V., Larrañaga, A., Gulis, V., Basaguren, A., Elosegi, A., Graça, M. A. S., & Pozo, J. (2015). The effects of eucalypt plantations on plant litter decomposition and macroinvertebrate communities in Iberian streams. *Forest Ecology and Management*, *335*, 129–138. https://doi.org/10.1016/j.foreco.2014.09.013

Ferreira, W. R., Ligeiro, R., Macedo, D. R., Hughes, R. M., Kaufmann, P. R., Oliveira, L. G., & Callisto, M. (2015). Is the diet of a typical shredder related to the physical habitat of headwater streams in the Brazilian Cerrado? *Annales de Limnologie*, *51*(2), 115–127. https://doi.org/10.1051/limn/2015004

Four, B., Arce, E., Danger, M., Gaillard, J., Thomas, M., & Banas, D. (2017). Catchment land use-dependent effects of barrage fishponds on the functioning of headwater streams. *Environmental Science and Pollution Research*, *24*(6), 5452–5468. https://doi.org/10.1007/s11356-016-8273-x

Fu, L., Jiang, Y., Ding, J., Liu, Q., Peng, Q.-Z., & Kang, M.-Y. (2016). Impacts of land use and environmental factors on macroinvertebrate functional feeding groups in the Dongjiang River basin, southeast China. *Journal of Freshwater Ecology*, *31*(1), 21–35. https://doi.org/10.1080/02705060.2015.1017847

Fugère, V., Jacobsen, D., Finestone, E. H., & Chapman, L. J. (2018). Ecosystem structure and function of afrotropical streams with contrasting land use. *Freshwater Biology*, *63*(12), 1498–1513. https://doi.org/10.1111/fwb.13178

Giling, D. P., Nally, R. M., & Thompson, R. M. (2015). How sensitive are invertebrates to riparian-zone replanting in stream ecosystems? *Marine and Freshwater Research*, *67*(10), 1500–1511. https://doi.org/10.1071/MF14360

Godoy, B. S., Simião-Ferreira, J., Lodi, S., & Oliveira, L. G. (2016). Functional process zones characterizing aquatic insect communities in streams of the Brazilian Cerrado. *Neotropical Entomology*, *45*(2), 159–169. https://doi.org/10.1007/s13744-015-0352-z

Goodman, K. J., Hershey, A. E., & Fortino, K. (2006). The effect of forest type on benthic macroinvertebrate structure and ecological function in a pine plantation in the North Carolina Piedmont. *Hydrobiologia*, *559*(1), 305–318. https://doi.org/10.1007/s10750-005-0990-y

Goss, C. W., Goebel, P. C., & Sullivan, S. M. P. (2014). Shifts in attributes along agriculture-forest transitions of two streams in central Ohio, USA. *Agriculture, Ecosystems & Environment*, *197*, 106–117. https://doi.org/10.1016/j.agee.2014.07.026

Göthe, E., Lepori, F., & Malmqvist, B. (2009). Forestry affects food webs in northern Swedish coastal streams. *Fundamental and Applied Limnology*, *175*(4), 281–294. https://doi.org/10.1127/1863-9135/2009/0175-0281

Guevara, G., Godoy, R., Boeckx, P., Jara, C., & Oyarzún, C. (2015). Effects of riparian forest management on Chilean mountain in-stream characteristics. *Ecohydrology & Hydrobiology*, *15*(3), 160–170. https://doi.org/10.1016/j.ecohyd.2015.07.003

Guevara, G., Godoy, R., & Franco, M. (2018). Linking riparian forest harvest to benthic macroinvertebrate communities in Andean headwater streams in southern Chile. *Limnologica*, *68*, 105–114. https://doi.org/10.1016/j.limno.2017.07.007

Hagen, E. M., Webster, J. R., & Benfield, E. F. (2006). Are leaf breakdown rates a useful measure of stream integrity along an agricultural landuse gradient? *Journal of the North American Benthological Society*, *25*(2), 330–343. https://doi.org/10.1899/0887-3593(2006)25[330:ALBRAU]2.0.CO;2

Hebert, T. A., Kuehn, K. A., & Halvorson, H. M. (2023). Land use differentially alters microbial interactions and detritivore feeding during leaf decomposition in headwater streams. *Freshwater Biology*, *68*(8), 1386–1399. https://doi.org/10.1111/fwb.14111

Hisabae, M., Sone, S., & Inoue, M. (2011). Breakdown and macroinvertebrate colonization of needle and leaf litter in conifer plantation streams in Shikoku, southwestern Japan. *Journal of Forest Research*, *16*(2), 108–115. https://doi.org/10.1007/s10310-010-0210-0

Hladyz, S., Tiegs, S. D., Gessner, M. O., Giller, P. S., Rîşnoveanu, G., Preda, E., Nistorescu, M., Schindler, M., & Woodward, G. (2010). Leaf-litter breakdown in pasture and deciduous woodland streams: A comparison among three European regions. *Freshwater Biology*, *55*(9), 1916–1929. https://doi.org/10.1111/j.1365-2427.2010.02426.x

Houghton, D. C., Berry, E. A., Gilchrist, A., Thompson, J., & Nussbaum, M. A. (2011). Biological changes along the continuum of an agricultural stream: Influence of a small terrestrial preserve and use of adult caddisflies in biomonitoring. *Journal of Freshwater Ecology*, *26*(3), 381–397. https://doi.org/10.1080/02705060.2011.563513

Huryn, A. D., Huryn, V. M. B., Arbuckle, C. J., & Tsomides, L. (2002). Catchment land-use, macroinvertebrates and detritus processing in headwater streams: Taxonomic richness versus function. *Freshwater Biology*, *47*(3), 401–415. https://doi.org/10.1046/j.1365-2427.2002.00812.x

Illyová, M., Beracko, P., & Krno, I. (2011). Influence of land use on hyporheos in catchment streams of the Velka Fatra Mts. *Biologia*, *66*(2), 320–327. https://doi.org/10.2478/s11756-011-0018-1

Iñiguez-Armijos, C., Hampel, H., & Breuer, L. (2018). Land-use effects on structural and functional composition of benthic and leaf-associated macroinvertebrates in four Andean streams. *Aquatic Ecology*, *52*(1), 77–92. https://doi.org/10.1007/s10452-017-9646-z

Iñiguez-Armijos, C., Rausche, S., Cueva, A., Sánchez-Rodríguez, A., Espinosa, C., & Breuer, L. (2016). Shifts in leaf litter breakdown along a forest–pasture–urban gradient in Andean streams. *Ecology and Evolution*, *6*(14), 4849–4865. https://doi.org/10.1002/ece3.2257

Inoue, M., Shinotou, S., Maruo, Y., & Miyake, Y. (2012). Input, retention, and invertebrate colonization of allochthonous litter in streams bordered by deciduous broadleaved forest, a conifer plantation, and a clear-cut site in southwestern Japan. *Limnology*, *13*(2), 207–219. https://doi.org/10.1007/s10201-011-0369-x

Ishikawa, N. F., Togashi, H., Kato, Y., Yoshimura, M., Kohmatsu, Y., Yoshimizu, C., Ogawa, N. O., Ohte, N., Tokuchi, N., Ohkouchi, N., & Tayasu, I. (2016). Terrestrial-aquatic linkage in stream food webs along a forest

chronosequence: Multi-isotopic evidence. *Ecology*, *97*(5), 1146–1158. https://doi.org/10.1890/15-1133.1
Jinggut, T., Yule, C. M., & Boyero, L. (2012). Stream ecosystem integrity is impaired by logging and shifting agriculture in a global megadiversity center (Sarawak, Borneo). *Science of the Total Environment*, *437*, 83–90. https://doi.org/10.1016/j.scitotenv.2012.07.062
Jyväsjärvi, J., Koivunen, I., & Muotka, T. (2020). Does the buffer width matter: Testing the effectiveness of forest certificates in the protection of headwater stream ecosystems. *Forest Ecology and Management*, *478*, 118532. https://doi.org/10.1016/j.foreco.2020.118532
Kadeka, E. C., Masese, F. O., Lusega, D. M., Sitati, A., Kondowe, B. N., & Chirwa, E. R. (2021). No difference in instream decomposition among upland agricultural and forested streams in Kenya. *Frontiers in Environmental Science*, *9*. https://doi.org/10.3389/fenvs.2021.794525
Kaylor, M. J., & Warren, D. R. (2018). Canopy closure after four decades of postlogging riparian forest regeneration reduces cutthroat trout biomass in headwater streams through bottom-up pathways. *Canadian Journal of Fisheries and Aquatic Sciences*, *75*(4), 513–524. https://doi.org/10.1139/cjfas-2016-0519
Kiffer Jr, W., Giuberti, T., Victor Serpa, K., Mendes, F., & Moretti, M. (2018). Do changes in riparian zones affect periphyton growth and invertebrate colonization on rocky substrates in Atlantic Forest streams? *Iheringia Série Zoologia*, *108*, 1–10. https://doi.org/10.1590/1678-4766e2018014
Kiffney, P. M., & Richardson, J. S. (2010). Organic matter inputs into headwater streams of southwestern British Columbia as a function of riparian reserves and time since harvesting. *Forest Ecology and Management*, *260*(11), 1931–1942. https://doi.org/10.1016/j.foreco.2010.08.016
Kominoski, J. S., Marczak, L. B., & Richardson, J. S. (2011). Riparian forest composition affects stream litter decomposition despite similar microbial and invertebrate communities. *Ecology*, *92*(1), 151–159. https://doi.org/10.1890/10-0028.1
Kreutzweiser, D. P., Good, K. P., Capell, S. S., & Holmes, S. B. (2008). Leaf-litter decomposition and macroinvertebrate communities in boreal forest streams linked to upland logging disturbance. *Journal of the North American Benthological Society*, *27*(1), 1–15. https://doi.org/10.1899/07-034R.1
Laćan, I., Resh, V. H., & McBRIDE, J. R. (2010). Similar breakdown rates and benthic macroinvertebrate assemblages on native and *Eucalyptus globulus* leaf litter in Californian streams. *Freshwater Biology*, *55*(4), 739–752. https://doi.org/10.1111/j.1365-2427.2009.02312.x
Lamberti, G. A., & Berg, M. B. (1995). Invertebrates and other benthic features as indicators of environmental change in Juday Creek, Indiana. *Natural Areas Journal*, *15*(3), 249–258.
Larrañaga, A., Perkins, D. M., Basaguren, A., Larrañaga, S., Pozo, J., & Montoya, J. M. (2023). Land use drives detritivore size structure and decomposition through shifts in resource quality and quantity. *Science of the Total Environment*, *892*, 164552. https://doi.org/10.1016/j.scitotenv.2023.164552
Lecerf, A., Baudoin, J.-M., Besson, A. A., Lamothe, S., & Lagrue, C. (2012). Is smaller necessarily better? Effects of small-scale forest harvesting on stream ecosystems. *Annales de Limnologie*, *48*(4), 401–409. https://doi.org/10.1051/limn/2012028
Lecerf, A., & Chauvet, E. (2008). Diversity and functions of leaf-decaying fungi in human-altered streams. *Freshwater Biology*, *53*(8), 1658–1672. https://doi.org/10.1111/j.1365-2427.2008.01986.x
Lecerf, A., & Richardson, J. S. (2010). Litter decomposition can detect effects of high and moderate levels of forest disturbance on stream condition. *Forest Ecology and Management*, *259*(12), 2433–2443. https://doi.org/10.1016/j.foreco.2010.03.022
Lemes da Silva, A. L., Lemes, W. P., Andriotti, J., Petrucio, M. M., & Feio, M. J. (2020). Recent land-use changes affect stream ecosystem processes in a subtropical island in Brazil. *Austral Ecology*, *45*(5), 644–658. https://doi.org/10.1111/aec.12879
Link, M., Schreiner, V. C., Graf, N., Szöcs, E., Bundschuh, M., Battes, K. P., Cîmpean, M., Sures, B., Grabner, D., Buse, J., & Schäfer, R. B. (2022). Pesticide effects on macroinvertebrates and leaf litter decomposition in areas with traditional agriculture. *Science of the Total Environment*, *828*, 154549. https://doi.org/10.1016/j.scitotenv.2022.154549
Lopes, M. P., Martins, R. T., Silveira, L. S., & Alves, R. G. (2015). The leaf breakdown of *Picramnia sellowii* (Picramniales: Picramniaceae) as index of anthropic disturbances in tropical streams. *Brazilian Journal of Biology*, *75*, 846–853. https://doi.org/10.1590/1519-6984.00414
Lorion, C. M., & Kennedy, B. P. (2009). Riparian forest buffers mitigate the effects of deforestation on fish assemblages in tropical headwater streams. *Ecological Applications*, *19*(2), 468–479. https://doi.org/10.1890/08-0050.1

Lubanga, H. L., Manyala, J. O., Sitati, A., Yegon, M. J., & Masese, F. O. (2021). Spatial variability in water quality and macroinvertebrate assemblages across a disturbance gradient in the Mara River Basin, Kenya. *Ecohydrology & Hydrobiology*, *21*(21), 718–730. https://doi.org/10.1016/j.ecohyd.2021.03.001

Mancuso, J., Tank, J. L., Mahl, U. H., Vincent, A., & Tiegs, S. D. (2023). Monthly variation in organic-matter decomposition in agricultural stream and riparian ecosystems. *Aquatic Sciences*, *85*(3), 83. https://doi.org/10.1007/s00027-023-00975-7

Maridet, L., Wasson, J.-G., Philippe, M., Amoros, C., & Naiman, R. (1998). Trophic structure of three streams with contrasting riparian vegetation and geomorphology. *Archiv für Hydrobiologie*, 61–85. https://doi.org/10.1127/archiv-hydrobiol/144/1998/61

Márquez, J. A., Principe, R. E., Cibils Martina, L., & Albariño, R. J. (2017). Pine needle litter acts as habitat but not as food source for stream invertebrates. *International Review of Hydrobiology*, *102*(1–2), 29–37. https://doi.org/10.1002/iroh.201601856

Martínez, A., Larrañaga, A., Pérez, J., Descals, E., Basaguren, A., & Pozo, J. (2013). Effects of pine plantations on structural and functional attributes of forested streams. *Forest Ecology and Management*, *310*, 147–155. https://doi.org/10.1016/j.foreco.2013.08.024

Masese, F. O., Kitaka, N., Kipkemboi, J., Gettel, G. M., Irvine, K., & McClain, M. E. (2014). Litter processing and shredder distribution as indicators of riparian and catchment influences on ecological health of tropical streams. *Ecological Indicators*, *46*, 23–37. https://doi.org/10.1016/j.ecolind.2014.05.032

Mckie, B. G., & Malmqvist, B. (2009). Assessing ecosystem functioning in streams affected by forest management: Increased leaf decomposition occurs without changes to the composition of benthic assemblages. *Freshwater Biology*, *54*(10), 2086–2100. https://doi.org/10.1111/j.1365-2427.2008.02150.x

McNeish, R. E., Benbow, M. E., & McEwan, R. W. (2017). Removal of the invasive shrub, *Lonicera maackii* (Amur Honeysuckle), from a headwater stream riparian zone shifts taxonomic and functional composition of the aquatic biota. *Invasive Plant Science and Management*, *10*(3), 232–246. https://doi.org/10.1017/inp.2017.22

McNeish, R. E., Moore, E. M., Benbow, M. E., & McEwan, R. W. (2015). Removal of the invasive shrub, *Lonicera maackii* , from riparian forests influences headwater stream biota and ecosystem function. *River Research and Applications*, *31*(9), 1131–1139. https://doi.org/10.1002/rra.2808

Mctammany, M. E., Benfield, E. F., & Webster, J. R. (2008). Effects of agriculture on wood breakdown and microbial biofilm respiration in southern Appalachian streams. *Freshwater Biology*, *53*(4), 842–854. https://doi.org/10.1111/j.1365-2427.2007.01936.x

Menéndez, M., Descals, E., Riera, T., & Moya, O. (2013). Do non-native *Platanus hybrida* riparian plantations affect leaf litter decomposition in streams? *Hydrobiologia*, *716*(1), 5–20. https://doi.org/10.1007/s10750-013-1539-0

Menninger, H. L., & Palmer, M. A. (2007). Herbs and grasses as an allochthonous resource in open-canopy headwater streams. *Freshwater Biology*, *52*(9), 1689–1699. https://doi.org/10.1111/j.1365-2427.2007.01797.x

Mesa, L. M., Reynaga, M. C., Correa, M. del V., & Sirombra, M. G. (2013). Effects of anthropogenic impacts on benthic macroinvertebrates assemblages in subtropical mountain streams. *Iheringia. Série Zoologia*, *103*, 342–349. https://doi.org/10.1590/S0073-47212013000400002

Mlambo, M. C., Paavola, R., Fritze, H., Louhi, P., & Muotka, T. (2019). Leaf litter decomposition and decomposer communities in streams affected by intensive forest biomass removal. *Ecological Indicators*, *101*, 364–372. https://doi.org/10.1016/j.ecolind.2019.01.035

Molinero, J., Pozo, J., & Gonzalez, E. (1996). Litter breakdown in streams of the Agüera catchment: Influence of dissolved nutrients and land use. *Freshwater Biology*, *36*(3), 745–756. https://doi.org/10.1046/j.1365-2427.1996.00125.x

Monroy, S., Martínez, A., López-Rojo, N., Pérez-Calpe, A. V., Basaguren, A., & Pozo, J. (2017). Structural and functional recovery of macroinvertebrate communities and leaf litter decomposition after a marked drought: Does vegetation type matter? *Science of the Total Environment*, *599–600*, 1241–1250. https://doi.org/10.1016/j.scitotenv.2017.05.093

Morabowen, A., Crespo-Pérez, V., & Ríos-Touma, B. (2019). Effects of agricultural landscapes and land uses in highly biodiverse tropical streams of the Ecuadorian Choco. *Inland Waters*, *9*(3), 289–300. https://doi.org/10.1080/20442041.2018.1527597

Murphy, J. F., & Giller, P. S. (2001). Hazel leaf breakdown in two low-order streams differing in the functional efficiency of their detritivore assemblages. *Archiv für Hydrobiologie*, 249–267. https://doi.org/10.1127/archiv-hydrobiol/150/2001/249

Musetta-Lambert, J., Muto, E., Kreutzweiser, D., & Sibley, P. (2017). Wildfire in boreal forest catchments influences leaf litter subsidies and consumer communities in streams: Implications for riparian management strategies. *Forest Ecology and Management*, *391*, 29–41. https://doi.org/10.1016/j.foreco.2017.01.028

Myers, L., Mihuc, T., & Woodcock, T. (2007). The impacts of forest management on invertebrate communities associated with submerged leaves in forested Adirondack streams. *Journal of Freshwater Ecology*, *22*(2), 325–331. https://doi.org/10.1080/02705060.2007.9665054

Oelbermann, M., & Gordon, A. M. (2000). Quantity and quality of autumnal litterfall into a rehabilitated agricultural stream. *Journal of Environmental Quality*, *29*(2), 603–611. https://doi.org/10.2134/jeq2000.00472425002900020031x

Oester, R., dos Reis Oliveira, P. C., Moretti, M. S., Altermatt, F., & Bruder, A. (2023). Leaf-associated macroinvertebrate assemblage and leaf litter breakdown in headwater streams depend on local riparian vegetation. *Hydrobiologia*, *850*(15), 3359–3374. https://doi.org/10.1007/s10750-022-05049-7

Ono, E. R., Manoel, P. S., Melo, A. L. U., & Uieda, V. S. (2020). Effects of riparian vegetation removal on the functional feeding group structure of benthic macroinvertebrate assemblages. *Community Ecology*, *21*(2), 145–157. https://doi.org/10.1007/s42974-020-00014-7

Paul, M. J., Meyer, J. L., & Couch, C. A. (2006). Leaf breakdown in streams differing in catchment land use. *Freshwater Biology*, *51*(9), 1684–1695. https://doi.org/10.1111/j.1365-2427.2006.01612.x

Principe, R. E., Márquez, J. A., Martina, L. C., Jobbágy, E. G., & Albariño, R. J. (2015). Pine afforestation changes more strongly community structure than ecosystem functioning in grassland mountain streams. *Ecological Indicators*, *57*, 366–375. https://doi.org/10.1016/j.ecolind.2015.04.033

Reid, D. J., Lake, P. S., & Quinn, G. P. (2013). Influences of agricultural landuse and seasonal changes in abiotic conditions on invertebrate colonisation of riparian leaf detritus in intermittent streams. *Aquatic Sciences*, *75*(2), 285–297. https://doi.org/10.1007/s00027-012-0273-4

Rezende, R. de S., Cararo, E. R., Bernardi, J. P., Chimello, V., Lima-Rezende, C. A., Albeny-Simões, D., Dal Magro, J., & Gonçalves, J. F. (2021). Land cover affects the breakdown of *Pinus elliottii* needles litter by microorganisms in soil and stream systems of subtropical riparian zones. *Limnologica*, *90*, 125905. https://doi.org/10.1016/j.limno.2021.125905

Riipinen, M. P., Fleituch, T., Hladyz, S., Woodward, G., Giller, P., & Dobson, M. (2009). Invertebrate community structure and ecosystem functioning in European conifer plantation streams. *Freshwater Biology*, *55*, 346–359. https://doi.org/10.1111/j.1365-2427.2009.02278.x

Roberts, M. L., & Bilby, R. E. (2009). Urbanization alters litterfall rates and nutrient inputs to small Puget Lowland streams. *Journal of the North American Benthological Society*, *28*(4), 941–954. https://doi.org/10.1899/07-160.1

Rossi, F., Mallet, C., Portelli, C., Donnadieu, F., Bonnemoy, F., & Artigas, J. (2019). Stimulation or inhibition: Leaf microbial decomposition in streams subjected to complex chemical contamination. *Science of the Total Environment*, *648*, 1371–1383. https://doi.org/10.1016/j.scitotenv.2018.08.197

Rubio-Ríos, J., Salinas-Bonillo, M. J., Pérez, J., Fenoy, E., Boyero, L., & Casas, J. J. (2023). Alder stands promote N-cycling but not leaf litter mass loss in Mediterranean streams flowing through pine plantations. *Forest Ecology and Management*, *542*, 121072. https://doi.org/10.1016/j.foreco.2023.121072

Ryan, D. K., & Kelly-Quinn, M. (2016). Riparian vegetation management for water temperature regulation: Implications for the production of macroinvertebrate prey of salmonids. *Fisheries Management and Ecology*, *23*(6), 519–530. https://doi.org/10.1111/fme.12193

Ryder, L., de Eyto, E., Gormally, M., Skeffington, M. S., Dillane, M., & Poole, R. (2011). Riparian zone creation in established coniferous forests in Irish upland peat catchments: Physical, chemical and biological Implications. *Biology and Environment: Proceedings of the Royal Irish Academy*, *111B*(1), 41–60.

Sakai, M., Natuhara, Y., Fukushima, K., Imanishi, A., Imai, K., & Kato, M. (2013). Ecological functions of persistent Japanese cedar litter in structuring stream macroinvertebrate assemblages. *Journal of Forest Research*, *18*(2), 190–199. https://doi.org/10.1007/s10310-012-0339-0

Sarriquet, P. E., Delettre, Y. R., & Marmonier, P. (2006). Effects of catchment disturbance on stream invertebrates: Comparison of different habitats (vegetation, benthic and interstitial) using bio-ecological groups. *Annales de Limnologie - International Journal of Limnology*, *42*(4), 205–219. https://doi.org/10.1051/limn/2006022

Shah, N. W., Nisbet, T. R., & Broadmeadow, S. B. (2021). The impacts of conifer afforestation and climate on water quality and freshwater ecology in a sensitive peaty catchment: A 25 year study in the upper River Halladale in North Scotland. *Forest Ecology and Management*, *502*, 119616. https://doi.org/10.1016/j.foreco.2021.119616

Silva-Araújo, M., Silva-Junior, E. F., Neres-Lima, V., Feijó-Lima, R., Tromboni, F., Lourenço-Amorim, C., Thomas, S. A., Moulton, T. P., & Zandonà, E. (2020). Effects of riparian deforestation on benthic invertebrate community and leaf processing in Atlantic forest streams. *Perspectives in Ecology and Conservation*, *18*(4), 277–282. https://doi.org/10.1016/j.pecon.2020.09.004

Suga, C. M., & Tanaka, M. O. (2013). Influence of a forest remnant on macroinvertebrate communities in a degraded tropical stream. *Hydrobiologia*, *703*(1), 203–213. https://doi.org/10.1007/s10750-012-1360-1

Tanaka, M. O., Fernandes, J. de F., Suga, C. M., Hanai, F. Y., & Souza, A. L. T. de. (2015). Abrupt change of a stream ecosystem function along a sugarcane-forest transition: Integrating riparian and in-stream characteristics. *Agriculture, Ecosystems & Environment*, *207*, 171–177. https://doi.org/10.1016/j.agee.2015.04.014

Tolkkinen, M., Mykrä, H., Annala, M., Markkola, A. M., Vuori, K. M., & Muotka, T. (2015). Multi-stressor impacts on fungal diversity and ecosystem functions in streams: Natural vs. anthropogenic stress. *Ecology*, *96*(3), 672–683. https://doi.org/10.1890/14-0743.1

Tonello, G., Decian, V. S., Restello, R. M., & Hepp, L. U. (2021). The conversion of natural riparian forests into agricultural land affects ecological processes in Atlantic forest streams. *Limnologica*, *91*, 125927. https://doi.org/10.1016/j.limno.2021.125927

Torres, P. J., & Ramírez, A. (2014). Land use effects on leaf litter breakdown in low-order streams draining a rapidly developing tropical watershed in Puerto Rico. *Revista de Biología Tropical*, *62*(S2), Article S2. https://doi.org/10.15517/rbt.v62i0.15783

Townsend, C. R., Downes, B. J., Peacock, K., & Arbuckle, C. J. (2004). Scale and the detection of land-use effects on morphology, vegetation and macroinvertebrate communities of grassland streams. *Freshwater Biology*, *49*(4), 448–462. https://doi.org/10.1111/j.1365-2427.2004.01192.x

Truchy, A., Sponseller, R. A., Ecke, F., Angeler, D. G., Kahlert, M., Bundschuh, M., Johnson, R. K., & McKie, B. G. (2022). Responses of multiple structural and functional indicators along three contrasting disturbance gradients. *Ecological Indicators*, *135*, 108514. https://doi.org/10.1016/j.ecolind.2021.108514

Tupinambás, T. H., Callisto, M., & Santos, G. B. (2007). Benthic macroinvertebrate assemblages structure in two headwater streams, south-eastern Brazil. *Revista Brasileira de Zoologia*, *24*(4), 887–897. https://doi.org/10.1590/S0101-81752007000400005

Turunen, J., Aroviita, J., Marttila, H., Louhi, P., Laamanen, T., Tolkkinen, M., Luhta, P.-L., Kløve, B., & Muotka, T. (2017). Differential responses by stream and riparian biodiversity to in-stream restoration of forestry-impacted streams. *Journal of Applied Ecology*, *54*(5), 1505–1514. https://doi.org/10.1111/1365-2664.12897

Valente-Neto, F., Koroiva, R., Fonseca-Gessner, A. A., & Roque, F. de O. (2015). The effect of riparian deforestation on macroinvertebrates associated with submerged woody debris. *Aquatic Ecology*, *49*(1), 115–125. https://doi.org/10.1007/s10452-015-9510-y

Walsh, C. J., Gooderham, J. P. R., Grace, M. R., Sdraulig, S., Rosyidi, M. I., & Lelono, A. (2002). The relative influence of diffuse- and point-source disturbances on a small upland stream in East Java, Indonesia: A preliminary investigation. *Hydrobiologia*, *487*(1), 183–192. https://doi.org/10.1023/A:1022990822198

Whiles, M. R., & Wallace, J. B. (1997). Leaf litter decomposition and macroinvertebrate communities in headwater streams draining pine and hardwood catchments. *Hydrobiologia*, *353*(1), 107–119. https://doi.org/10.1023/A:1003054827248

Yeung, A. C. Y., Lecerf, A., & Richardson, J. S. (2017). Assessing the long-term ecological effects of riparian management practices on headwater streams in a coastal temperate rainforest. *Forest Ecology and Management*, *384*, 100–109. https://doi.org/10.1016/j.foreco.2016.10.044

Youngquist, M. B., Wiley, C., Eggert, S. L., D'Amato, A. W., Palik, B. J., & Slesak, R. A. (2020). Foundation species loss affects leaf breakdown and aquatic invertebrate resource use in black ash wetlands. *Wetlands*, *40*(4), 839–852. https://doi.org/10.1007/s13157-019-01221-3

Zhang, Y., Richardson, J. S., & Pinto, X. (2009). Catchment-scale effects of forestry practices on benthic invertebrate communities in Pacific coastal streams. *Journal of Applied Ecology*, *46*(6), 1292–1303. https://doi.org/10.1111/j.1365-2664.2009.01718.x

Zúñiga-Sarango, W., Gaona, F. P., Reyes-Castillo, V., & Iñiguez-Armijos, C. (2020). Disrupting the biodiversity–ecosystem function relationship: Response of shredders and leaf breakdown to urbanization in Andean streams. *Frontiers in Ecology and Evolution*, *8*. https://doi.org/10.3389/fevo.2020.592404
